# Skin Barrier-Informed Topical and Transdermal Drug Delivery: Excipient-Driven Strategies, Vehicle Transformation, and Translational Challenges

**DOI:** 10.3390/pharmaceutics18091069

**Published:** 2026-08-26

**Authors:** Binaya Sapkota, Susmita Phuyal, Arjun Dhwoj Bamjan, Seung-Sik Cho, Jung-Hyun Shim, Jin Woo Park, Laxman Subedi

**Affiliations:** 1 Department of Biomedicine, Health & Life Convergence Sciences, BK21 Four, Biomedical and Healthcare Research Institute, Mokpo National University, Muan-gun 58554, Jeonnam, Republic of Korea; phr.binaya@gmail.com (B.S.); sushmitaphuyal54@gmail.com (S.P.); arjun.bamjan@gmail.com (A.D.B.); sjason1@naver.com (S.-S.C.); s1004jh@gmail.com (J.-H.S.); 2 College of Pharmacy and Natural Medicine Research Institute, Mokpo National University, Muan-gun 58554, Jeonnam, Republic of Korea; 3 Biomedicine Cutting Edge Formulation Technology Center, Mokpo National University, Muan-gun 58554, Jeonnam, Republic of Korea

**Keywords:** skin barrier, transdermal drug delivery, permeation enhancers, vesicular carriers

## Abstract

Topical and transdermal dosage forms can localize therapy or provide controlled systemic exposure, but translation is limited by the selective stratum corneum (SC) barrier and post-application changes in formulation. Existing reviews often address individual enhancers or carriers without integrating drug properties, vehicle microstructure, post-application transformation, quality of evidence, and product development constraints. This review fills that gap by comparing passive formulations, chemical permeation enhancers, vesicular and lipid-based carriers, supersaturating systems, and physical barrier bypass technologies within a skin barrier-informed framework. Increased permeation alone does not establish translational value. Passive delivery remains most feasible for potent, moderately lipophilic small molecules. Chemical enhancers are scalable but limited by irritation and drug-dependent compatibility; nanocarriers may improve solubilization and cutaneous deposition but often lack human confirmation; and physical methods broaden delivery to macromolecules while adding device, manufacturing, usability, and regulatory burdens. Solvent evaporation, residual film composition, supersaturation, precipitation, and drug–vehicle affinity further determine the effective post-application driving force. Accordingly, we propose an evidence-ranked framework linking payload properties and target compartment to mechanism, safety, clinical readiness, and regulatory complexity. It distinguishes mechanistic promise from clinically demonstrated delivery and identifies the evidence needed to advance reproducible topical and transdermal products.

## 1. Introduction

Topical and transdermal dosage forms provide noninvasive routes for local or systemic therapy and offer patient-friendly alternatives to oral or injectable administration [1,2,3]. For systemic therapy, transdermal delivery can avoid gastrointestinal degradation and hepatic first-pass metabolism. Its clinical value is demonstrated by locally acting dermatologic products and transdermal systems used for cardiovascular, analgesic, hormonal, neurological, and smoking cessation therapies [4,5,6]. Nevertheless, the commercial success of passive transdermal delivery remains restricted to a relatively narrow physicochemical space. Most marketed drugs are potent, chemically stable small molecules with molecular weights below approximately 500 Da, moderate lipophilicity, and daily dose requirements compatible with the limited delivery area and flux of a transdermal system [7,8]. The principal barrier is the stratum corneum (SC), in which protein-rich corneocytes are surrounded by an ordered intercellular lipid matrix. Large, extensively ionized, or highly hydrophilic molecules generally partition poorly into this barrier, whereas excessively lipophilic compounds may be retained within the SC rather than reaching the viable epidermis, dermis, or systemic circulation [4,5,6,7,8]. Delivery therefore depends on more than the nominal drug concentration. Drug solubility in the vehicle, free drug fraction, saturation state, partitioning into SC lipids, diffusivity, target tissue depth, and skin condition collectively determine whether a formulation provides adequate local deposition or systemic exposure.

A further challenge is the fact that topical and transdermal formulations are not compositionally static after application. Volatile solvents may evaporate, water may be absorbed or lost, emulsion phases may redistribute, and the residual film may become progressively enriched in nonvolatile excipients. These transformations can increase drug thermodynamic activity and temporarily enhance permeation, but they may also induce crystallization, reduce molecular mobility, alter occlusion, or increase irritation [9,10,11,12]. Consequently, formulation performance cannot be reliably predicted from the composition in the container alone; the applied and transformed states must also be considered. Formulation strategies can modify this drug–vehicle–skin relationship through different mechanisms. Conventional vehicles and excipients regulate solubilization, drug release, residence time, and deposition within the intended skin compartment [13,14,15]. Vesicular and lipid-based carriers can improve the apparent solubility of poorly water-soluble drugs, protect unstable compounds, and promote epidermal or follicular targeting [16,17]. Chemical permeation enhancers (CPEs) alter SC lipids, keratin interactions, or drug partitioning, whereas microneedles (MNs), iontophoresis, electroporation, ultrasound, and thermal or laser-based approaches physically bypass or transiently perturb the skin barrier [18,19,20]. However, enhanced delivery may be accompanied by irritation, barrier damage, formulation metastability, device dependence, dose loading limitations, manufacturing complexity, or regulatory uncertainty.

**Figure 1:**
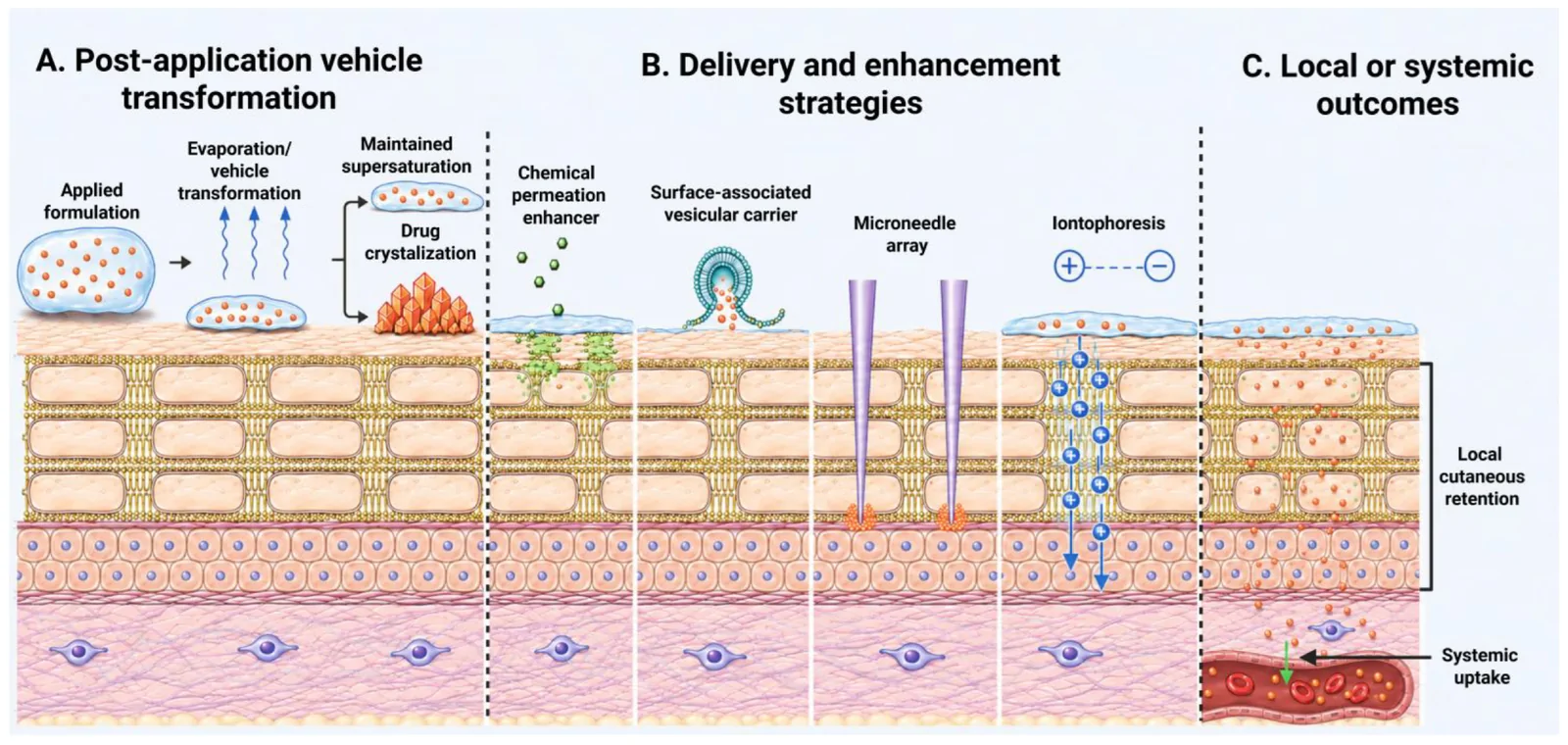
Integrated framework for skin barrier-informed topical and transdermal drug delivery. (**A**) Post-application vehicle transformation may maintain a supersaturated drug-rich film or induce surface crystallization. (**B**) Chemical enhancers, surface-associated vesicular carriers, microneedles, and iontophoresis improve delivery through distinct barrier modulation or bypass mechanisms. (**C**) Released drug may be retained in viable cutaneous tissues or reach the dermal circulation for systemic uptake. Schematic not to scale.

Although these technologies have been extensively reviewed, the literature remains fragmented. Previous reviews have generally focused on skin barrier biology, conventional dosage forms, individual excipient classes, nanocarrier systems, or specific physical enhancement technologies as separate topics. However, research has not yet adequately addressed a unified, cross-platform framework that connects barrier mechanism, excipient function, thermodynamic activity, and post-application vehicle transformation with target tissue requirements, payload properties, strength of evidence, safety, manufacturability, and translational readiness. This distinction is important because many studies demonstrate increased permeation using synthetic membranes, excised animal skin, or short-duration laboratory models, whereas substantially fewer have established reproducible delivery in human skin, clinical benefit, repeated use tolerability, long-term stability, scalable production, or regulatory feasibility [21,22]. An experimental increase in flux therefore does not necessarily indicate that a formulation is clinically or commercially viable. Accordingly, this review has four specific objectives. First, it defines the biological, physicochemical, and formulation determinants governing local skin deposition and systemic transdermal transport. Second, it compares conventional vehicles, excipient-based approaches, vesicular and lipid-based carriers, and physical enhancement technologies according to their mechanisms, suitable payloads, expected benefits, safety trade-offs, and principal failure modes. Third, it critically distinguishes evidence derived from in vitro, ex vivo, animal, human pharmacokinetic, clinical, regulatory, and commercial sources and identifies areas in which findings are consistent, controversial, or conflicting. Fourth, it integrates these dimensions into a strategy selection and translational readiness framework that distinguishes clinically established approaches from technologies approaching translation and those that remain primarily experimental (Figure 1). The purpose is not to identify a universally superior delivery platform but to clarify the evidence and design criteria required to match a delivery strategy to a defined drug, target compartment, and intended product profile.

## 2. Literature Search Strategy and Methodology

**Figure 2:**
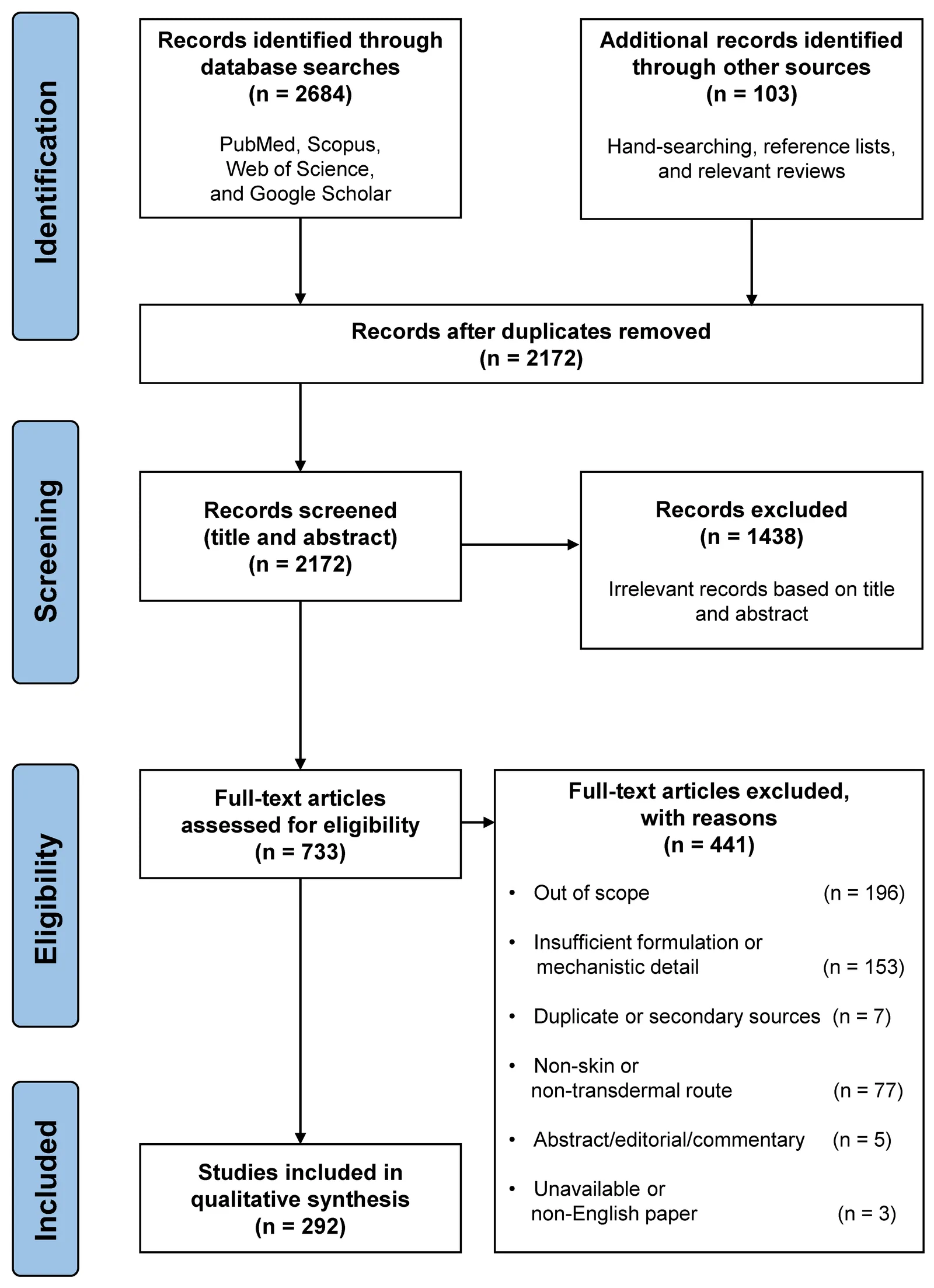
PRISMA flow diagram illustrating the identification, screening, eligibility assessment, and inclusion of studies included in this review.

A structured narrative review was conducted using a workflow informed by the Preferred Reporting Items for Systematic Reviews and Meta-Analyses (PRISMA) framework for literature identification, screening, and study selection. PubMed, Scopus, Web of Science, and Google Scholar were searched for relevant publications from January 2015 through 31 July 2026 (final search date), while earlier seminal studies were retained when necessary to explain skin barrier biology, permeation mechanisms, or transdermal transport theory. The search strategy combined terms related to topical and transdermal drug delivery, skin permeation, CPEs, vesicular and lipid-based carriers, MNs, iontophoresis, electroporation, ultrasound-mediated delivery, supersaturation, thermodynamic activity, and post-application vehicle transformation. Eligible publications were those that investigated topical or transdermal formulations, permeation enhancers, carriers, or physical delivery devices and reported at least one interpretable association among formulation composition, barrier interaction, drug release, skin deposition, permeation, pharmacokinetics, safety, clinical outcomes, manufacturing, or regulatory performance. For the purpose of critical synthesis, “sufficient mechanistic insight” was defined as the availability of adequate formulation or device information, an appropriate comparator, a relevant experimental or clinical model, quantitative delivery or barrier-related data, and sufficient information to evaluate the relationship between the proposed mechanism and the reported findings. Studies reporting only therapeutic outcomes without mechanistic interpretation were generally excluded unless they provided important translational or regulatory insights. Literature screening and study selection were independently performed by two reviewers. Any disagreements regarding study eligibility or evidence classification were resolved through discussion until consensus was reached. For qualitative synthesis, the included evidence was categorized as in vitro, ex vivo, animal in vivo, human pharmacokinetic or skin distribution, controlled clinical, regulatory approval, or commercial evidence. The literature identification and screening process is summarized in Figure 2.

## 3. Skin Barrier and Permeation Pathways

The skin is a multilayered organ comprising the epidermis, dermis, and subcutaneous tissue. For most topically applied molecules, the greatest diffusional resistance is encountered in the SC, the outermost layer of the epidermis. The SC consists of flattened, keratin-rich corneocytes surrounded by a continuous extracellular lipid matrix enriched in ceramides, cholesterol, and free fatty acids [23,24,25]. Its low water content, winding diffusion pathways, and highly ordered lipid lamellae create an effective barrier despite its limited thickness. Barrier function is not constant; anatomical site, age, hydration, temperature, cleansing, occlusion, and prior treatment can alter SC organization and diffusional resistance. These sources of variability should therefore be considered experimental and clinical covariates rather than background factors. Passive permeation is commonly described in terms of intercellular, transcellular, and appendageal pathways. The intercellular route follows the continuous lipid domains surrounding corneocytes and is generally favored by small, moderately lipophilic molecules. In contrast, the transcellular route requires repeated partitioning between keratin-rich corneocytes and extracellular lipid domains, imposing a substantial energetic penalty that limits its contribution for many drugs. These pathways are conceptual rather than mutually exclusive because a molecule may sample multiple microdomains while traversing the heterogeneous SC. Molecular size, ionization, hydrogen bonding capacity, conformational flexibility, and partitioning therefore act together, and no single physicochemical descriptor reliably predicts delivery across all formulations [26,27,28]. Pathway-resolved imaging studies further demonstrate that topically applied compounds may exhibit heterogeneous and spatially distinct penetration patterns within the skin [29].

The empirical 500 Da guideline remains useful for identifying molecules that are unlikely to cross intact skin efficiently by passive diffusion, but it should not be interpreted as an absolute mechanistic threshold [8]. Small molecules may still exhibit inadequate delivery when their dose requirement is high, their aqueous or lipid solubility is insufficient, or their affinity for the vehicle exceeds their affinity for the SC. Conversely, measurable delivery of larger molecules may occur when the barrier is disrupted, deliberately bypassed, or accessed through appendageal pathways. Lipophilicity also exhibits an optimum rather than monotonic relationship with flux; insufficient lipophilicity limits partitioning into SC lipids, whereas excessive lipophilicity can promote retention within the SC and restrict transfer into viable tissue. Ionization introduces an additional trade-off because the unionized fraction generally partitions more readily into lipid domains, while the ionized fraction may provide greater aqueous solubility. After crossing the SC, a drug enters the more hydrated environments of the viable epidermis and dermis. These layers may become rate-limiting for highly lipophilic compounds that leave the SC slowly or bind strongly to viable tissue. Cutaneous phase I and phase II metabolic enzymes may also transform labile drugs or prodrugs, thereby altering local exposure and the fraction available for systemic uptake [30,31]. For locally acting products, retention within the epidermis, dermis, or appendages may be the intended endpoint, and high systemic flux may be unnecessary or undesirable. For systemic transdermal products, the drug must traverse viable tissue and reach the dermal microcirculation at a reproducible rate. Consequently, increased SC uptake may represent successful targeting for a local product, sequestration for a systemic product, or a safety concern when systemic exposure is unintended. Delivery performance must therefore be interpreted according to the intended tissue compartment.

Hair follicles, associated sebaceous glands, and sweat ducts form discontinuous appendageal pathways. Although follicular openings occupy only a small fraction of the skin surface, they can provide relatively low-resistance entry and serve as reservoirs for particles, lipophilic compounds, and formulations targeted to the pilosebaceous unit [32,33]. Sweat ducts provide an additional shunt pathway, although their limited surface area generally restricts their contribution to passive delivery. Their involvement may become more relevant for ionic or hydrophilic compounds under electrically assisted conditions. Preferential appendageal targeting should not be inferred solely from particle size or fluorescence near an appendage. Quantitative recovery from separated compartments, pathway-resolved imaging with appropriate controls, and comparison with non-appendageal transport are required to establish pathway-specific delivery. Barrier-modifying and barrier-bypassing technologies can change the relative importance of these pathways. CPEs modify SC lipid organization, protein interactions, solvent properties, or drug partitioning, whereas hydration and occlusion can expand aqueous domains and increase lipid mobility. MNs and fractional devices create transient microchannels, while iontophoresis provides additional electrical and electroosmotic driving forces [18,19,20,24]. Once a pathway is externally modified or created, device geometry, applied energy, skin recovery, dose loading, and contact between the formulation and the newly accessible pathway become integral components of the delivery mechanism. The relevant comparison is therefore not simply between passive and enhanced delivery; rather, it concerns how each intervention redistributes barrier resistance and which additional safety, reproducibility, or control variables it introduces.

**Figure 3:**
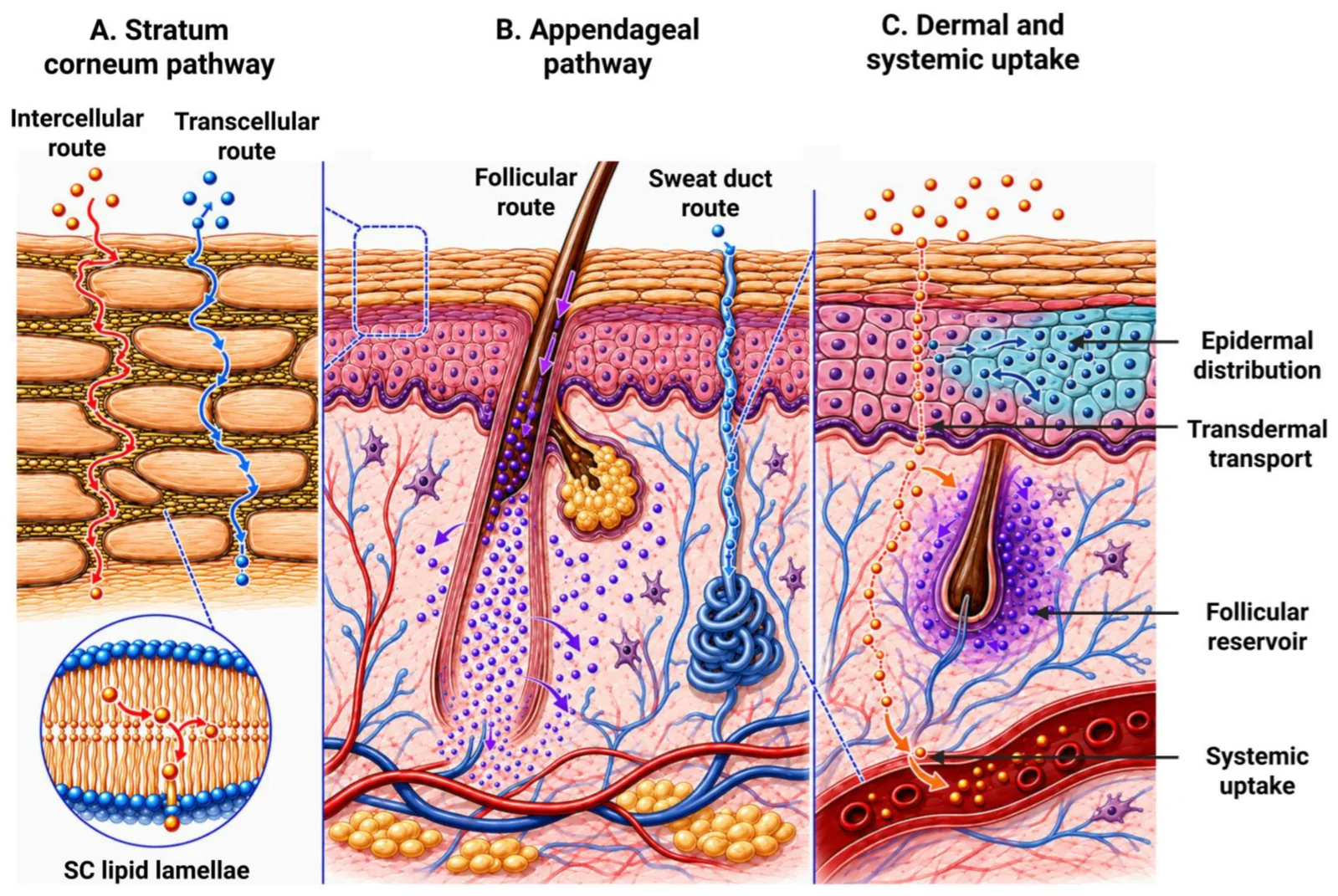
Major cutaneous penetration pathways and drug disposition following topical application. (**A**) Drugs cross the stratum corneum (SC) mainly through intercellular or transcellular pathways. (**B**) Hair follicles and sweat ducts provide appendageal pathways and potential drug reservoirs. (**C**) Penetrated drug may remain within viable skin, accumulate in follicular compartments, or reach the dermal circulation for systemic uptake. The illustration is schematic and not drawn to scale.

These principles establish three formulation questions used throughout this review. First, what is the intended therapeutic destination: the SC, viable epidermis, dermis, follicular compartment, or systemic circulation? Second, which step is rate-limiting: drug release from the dosage form, partitioning into the SC, diffusion across the barrier, transfer into viable tissue, or access to the dermal circulation? Third, does the proposed enhancer improve the rate-limiting step without introducing unacceptable irritation, instability, variability, or device burden? The major cutaneous penetration pathways and the subsequent local or systemic disposition of topically applied drugs are summarized in Figure 3. Their principal characteristics, representative payloads, and relevant molecular size ranges are compared in Table 1.

## 4. Formulation Determinants for Effective Delivery

The formulation determines the concentration and physical state of the drug presented to the skin and thereby mediates the relationship between the intrinsic properties of the drug and its transport across the skin barrier. Its functions extend from maintaining product stability and dose uniformity to controlling drug release, partitioning into the SC, residence at the application site, and the microenvironment established at the formulation–skin interface. These functions are closely interdependent. Increasing drug solubility may reduce the risk of crystallization but also strengthen drug retention within the vehicle, whereas increasing viscosity may improve residence time while restricting drug diffusion and release. Similarly, occlusion may enhance SC hydration but may also alter formulation composition, adhesion, and local tolerability. Formulation optimization should therefore be guided by the intended target compartment and the rate-limiting step in delivery rather than by maximizing an individual property. Drug solubility and saturation state are central determinants of release and skin partitioning. A drug must remain sufficiently soluble to ensure manufacturing feasibility, dose uniformity, and reproducible application; however, a high total drug concentration does not necessarily result in a high permeation flux. The driving force for release and skin transport is more closely related to the chemical potential or thermodynamic activity of the drug than to its nominal concentration. Strong solubilization by surfactants, oils, polymers, or cyclodextrins (CDs) may reduce the freely available drug fraction and its tendency to partition into the SC, even when the total drug concentration is high [9,10]. Saturated formulations generally provide a relatively high and reproducible thermodynamic driving force, whereas supersaturated formulations may temporarily provide greater activity but are susceptible to nucleation and crystal growth. Evaluation of solubility-related performance should therefore include equilibrium solubility in the complete vehicle, saturation ratio, free or unbound drug fraction where measurable, drug release behavior, crystallization induction time, and the physical state of the drug.

Rheological and microstructural properties determine how the formulation is dispensed, spread, retained, and depleted at the application site. Creams, gels, ointments, foams, emulsions, and suspensions are structured systems whose yield stress, shear-thinning behavior, thixotropic recovery, and phase organization may change during storage, dispensing, and rubbing. Increased viscosity or yield stress may reduce runoff and prolong skin contact but may also decrease molecular diffusion and drug release from the vehicle. A single measurement of apparent viscosity is therefore insufficient to predict delivery performance. Rheological characterization should cover shear conditions relevant to storage, dispensing, spreading, and structural recovery and should be interpreted together with measurements of dose uniformity, film formation, drug release, skin retention, and permeation. Vehicle polarity and drug–excipient affinity further regulate the distribution of the drug between the formulation and the skin. Depending on the composition of the vehicle, the drug may remain in the continuous phase, partition into dispersed oil or aqueous domains, associate with surfactant aggregates, or interact with polymers and other excipients. Excessive affinity for the vehicle can limit drug release and transfer into the SC, whereas insufficient affinity can promote phase separation, precipitation, or dose nonuniformity. Logarithm of partition coefficient (LogP) and logarithm of distribution coefficient (logD) provide useful initial information regarding drug lipophilicity, but cannot fully describe partitioning between a multicomponent formulation and the heterogeneous lipid and protein domains of the SC. Co-solvents such as ethanol, propylene glycol (PG), polyethylene glycols (PEG), and diethylene glycol monoethyl ether may simultaneously alter drug solubility, vehicle polarity, drug–skin partitioning, and SC lipid organization. Their net effects must therefore be evaluated in the final formulation rather than inferred from the properties of an individual excipient.

Hydration and occlusion can modify both barrier function and formulation performance. Occlusive ointments, films, and patches reduce transepidermal water loss (TEWL) and increase SC hydration, which can enhance molecular mobility within keratin-rich domains and alter intercellular lipid organization. These changes may increase passive permeation, although the magnitude of enhancement depends on the drug, anatomical site, formulation, and duration of occlusion. Prolonged or excessive occlusion may also cause maceration, impair adhesion, promote microbial growth, or increase the penetration of irritants. Occlusion should therefore be treated as a controlled formulation and use condition, with consideration of wear time, anatomical location, water vapor transmission, barrier recovery, adhesion, and repeated use tolerability. Physical and chemical stability are directly linked to dose reproducibility and delivery performance. Emulsion breakdown, particle or vesicle aggregation, particle growth, polymorphic conversion, drug degradation, adhesive cold flow, and loss or redistribution of volatile excipients can alter the amount of drug applied, released, and transported into the skin. Accelerated stability studies are useful for identifying potential failure mechanisms but cannot replace long-term, in-use, and packaging-specific evaluations. Drug release and skin delivery performance should be compared before and after storage, and critical quality attributes should be selected according to their potential effects on dose, flux, tissue exposure, safety, and clinical performance rather than solely on appearance, nominal viscosity, or mean particle size.

Rational formulation selection should begin by defining the intended target compartment and the exposure required to achieve the desired therapeutic effect. The principal limitation should then be identified as drug solubility, release from the vehicle, partitioning into the SC, diffusion through the barrier, distribution within viable skin, or access to the systemic circulation. Excipients and dosage form characteristics should be selected to address this limitation while maintaining stability, dose reproducibility, tolerability, and practical usability. Formulation performance should subsequently be evaluated using an appropriate skin model, clinically relevant dosing conditions, and comparators capable of distinguishing the drug, vehicle, and enhancement effects. Because the relevant formulation attributes may continue to evolve after application, the following section examines how post-application vehicle transformation, supersaturation, and drug precipitation influence delivery performance.

## 5. Post-Application Vehicle Transformation and Drug Precipitation

### 5.1. Post-Application Vehicle Transformation

**Figure 4:**
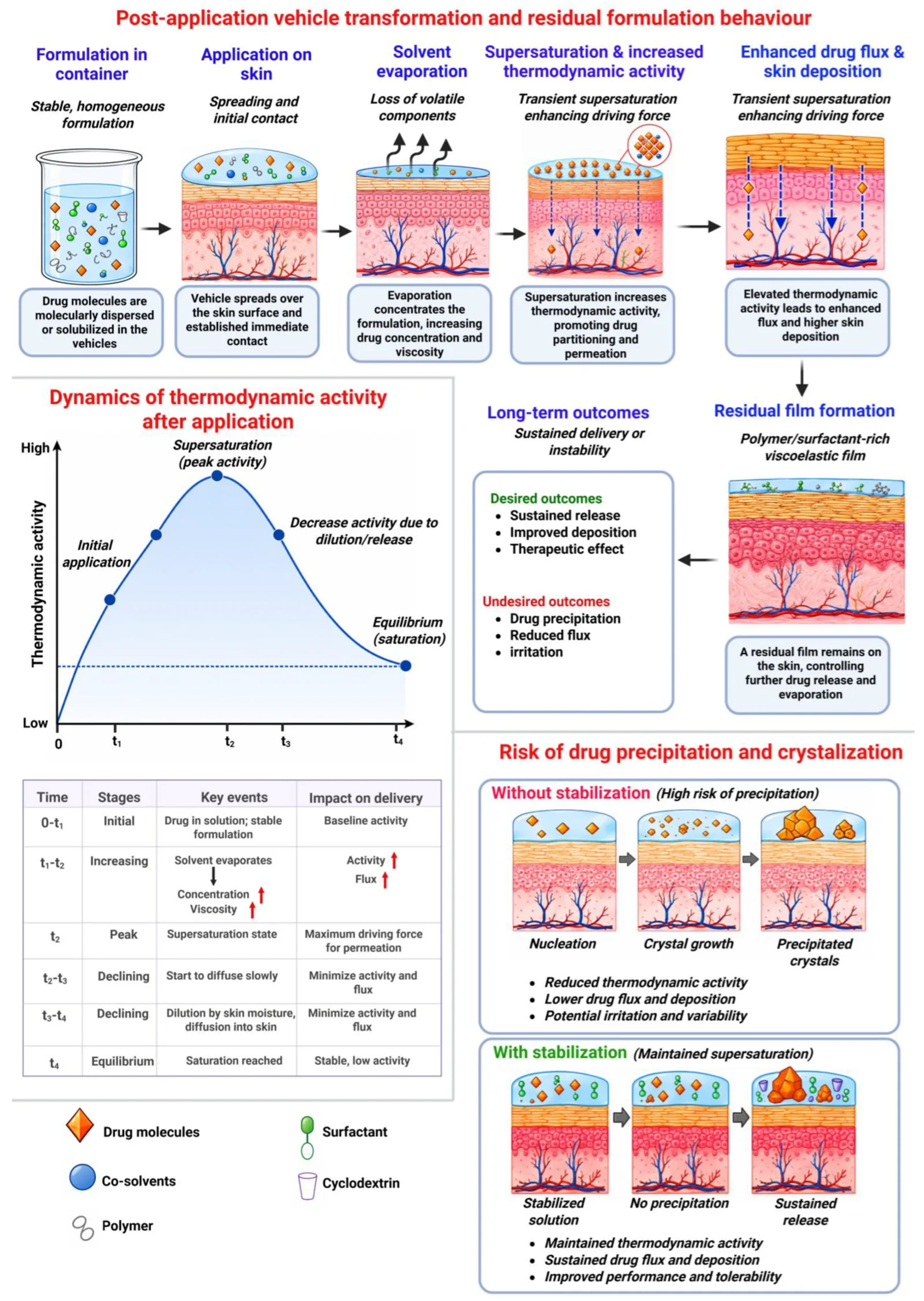
Post-application vehicle transformation and residual formulation behavior in topical and transdermal drug delivery. Following skin application, the evaporation of volatile solvents alters vehicle composition and concentrates the residual formulation, leading to transient supersaturation and increased thermodynamic activity. This metastable state enhances drug partitioning into the stratum corneum, increases transdermal flux, and promotes skin deposition. However, the residual film may either maintain a beneficial supersaturated state for sustained drug delivery or undergo nucleation and crystallization, resulting in reduced thermodynamic activity, decreased permeation, increased inter-subject variability, and potential skin irritation. The balance between supersaturation maintenance and precipitation ultimately determines the effectiveness of topical and transdermal delivery systems.

Post-application vehicle transformation is a critical determinant of topical and transdermal performance because a formulation rarely remains compositionally unchanged after application. The evaporation of volatile components such as water or ethanol progressively concentrates nonvolatile excipients, alters solvent polarity and microstructure, and may induce phase inversion, coalescence, supersaturation, precipitation, or crystallization (Figure 4). Among these changes, supersaturation is particularly important because it occurs when the drug concentration in the residual vehicle exceeds its equilibrium solubility. This metastable state increases the drug chemical potential and strengthens the driving force for partitioning into the SC. Consequently, a supersaturated residual film may produce greater skin deposition or permeation than a saturated or undersaturated formulation, even when the nominal drug dose is similar. The benefit of supersaturation depends on both its magnitude and duration. A supersaturated state that dissipates rapidly may provide insufficient time for drug partitioning into the skin, whereas excessive supersaturation can accelerate nucleation and crystal growth. The formulation objective is therefore not simply to maximize supersaturation but to maintain a sufficiently long metastable window without precipitation. Once the residual vehicle can no longer retain the excess drug in solution, crystallization reduces the relative thermodynamic activity (*a*) from a supersaturated state (*a* > 1) toward unity. Under sink conditions and assuming an approximately constant skin permeability coefficient, this relationship can be expressed as *J* = *K_p_* × *a* × *C_s_*, where *J* is skin flux, *K_p_* is the skin permeability coefficient, *C_s_* is the equilibrium saturation solubility, and *a* is the drug activity relative to saturation. The resulting crystals generally remain on the skin surface rather than partitioning into the SC, thereby reducing delivery and increasing performance variability [43,44].

Nucleation is the rate-limiting kinetic event that initiates precipitation in supersaturated topical systems. Increasing solvent loss and the degree of supersaturation promotes molecular association and facilitates the formation of clusters that may develop into stable crystal nuclei [45]. Homogeneous nucleation requires the spontaneous formation of a new crystal–solution interface and therefore generally occurs only at high supersaturation. Under practical application conditions, heterogeneous nucleation is more likely because SC lipids, desquamated material, suspended particulates, formulation impurities, and surface irregularities reduce the interfacial energy barrier and permit nuclei to form at lower supersaturation levels [43]. For a molecular cluster to persist and grow, it must exceed the critical nucleus radius (*r_c_*), which is proportional to interfacial tension (*γ*) and inversely related to the supersaturation driving force (Δμ). Clusters smaller than *r_c_* tend to redissolve, whereas larger clusters can grow through continued molecular deposition. Increasing supersaturation decreases both *r_c_* and the critical free energy barrier (ΔG_crit_), thereby increasing the nucleation rate exponentially [46]. Precipitation can therefore occur within minutes in rapidly evaporating topical films, followed by crystal growth and coarsening. This process counteracts the permeability advantage generated by supersaturation and highlights the need for formulation strategies that extend the metastable state while suppressing nucleation and crystal growth [47].

### 5.2. Strategies to Maintain Supersaturation and Control Post-Application Drug Precipitation

Controlling post-application drug precipitation is essential for preserving the permeation advantage generated by supersaturation (Figure 4). Because the residual film at the skin surface, rather than the formulation originally contained in the package, ultimately governs drug activity and delivery, stabilization strategies should be designed around the transformed vehicle. Adjusting the continuous phase, lipid content, and relative proportions of volatile and nonvolatile components can reduce excessive drug–vehicle affinity and increase thermodynamic activity. However, rapid solvent loss may abruptly decrease residual drug solubility, trigger crystallization, and reduce the free-drug concentration available for skin partitioning. Formulation performance should therefore be evaluated by monitoring evaporation, residual composition, drug state, and delivery throughout the intended application period.

Antinucleant polymers are widely used to kinetically stabilize supersaturated topical and transdermal formulations. Rather than permanently preventing molecular clustering, these polymers delay nucleation and crystal growth, thereby extending the metastable period during which high drug activity can be maintained. Cellulosic polymers, including hydroxypropyl methylcellulose (HPMC), hydroxypropyl cellulose (HPC), methylcellulose (MC), and sodium carboxymethyl cellulose (NaCMC), can adsorb onto emerging crystal surfaces through hydrogen bonding and steric interactions, reducing further molecular attachment [43]. Polyvinylpyrrolidone (PVP), particularly PVP K30, can similarly occupy crystal growth sites and form a diffusion-limiting polymeric layer around developing nuclei. Polymer effectiveness remains drug- and vehicle-dependent and is influenced by crystal lattice chemistry, drug hydrophobicity, residual solvent composition, polymer–drug affinity, and viscosity. Increased viscosity may provide additional stabilization by reducing molecular diffusion toward crystal surfaces [48,49]. CDs and surfactants provide complementary stabilization by controlling drug solubility and molecular availability in the residual vehicle. CDs such as 2-hydroxypropyl-β-CD form reversible inclusion complexes with hydrophobic drugs, creating a dynamic reservoir that buffers the free drug concentration and reduces the probability of uncontrolled precipitation [50]. Surfactants such as sodium lauryl sulfate (SLS), D-α-tocopheryl polyethylene glycol 1000 succinate (TPGS), poloxamers, and polysorbates can solubilize poorly water-soluble drugs within micellar or mixed micellar domains, reduce interfacial tension, improve the wetting of drug-rich residues, and inhibit heterogeneous nucleation [43,51]. For example, a supersaturated ibuprofen system containing vitamin E TPGS and HPMC delayed crystallization while improving skin permeation, demonstrating the complementary effects of surfactant-mediated solubilization and polymeric antinucleation [52]. Nevertheless, excessive complexation or micellar solubilization may reduce free drug activity and skin partitioning; therefore, CD and surfactant concentrations should be optimized to balance physical stability with permeation efficiency.

Solvent and co-solvent engineering is particularly important in evaporative formulations. Volatile solvents such as ethanol and isopropanol can rapidly generate supersaturation by concentrating the drug as the vehicle dries, but an abrupt loss of solubilization capacity may initiate nucleation and crystallization [53,54,55,56]. In volatile/nonvolatile vehicle studies, isopropanol-containing formulations increased the fluocinolone penetration by approximately 8–10-fold, although further enhancement was restricted by steroid precipitation [53]. Nonvolatile or slowly evaporating components—including propylene glycol, glycerol, polyethylene glycol 400 (PEG 400), Transcutol^®^ (diethylene glycol monoethyl ether, DEGEE), isopropyl myristate (IPM), and medium-chain lipids—can moderate this transition by maintaining residual solvent capacity after drying [53,56,57,58]. These excipients act as solubilizing reservoirs, delay formation of a drug-rich dry phase, and help retain the drug in a molecularly dispersed or amorphous state. Some may also modify drug partitioning into the SC or interfere with crystal packing, further reducing post-application recrystallization [57,58]. Nanoparticle-based strategies can further reduce precipitation by restricting drug mobility and modifying phase behavior within the residual formulation. Polymeric nanoparticles and solid lipid nanoparticles (SLNs) may retain drugs in amorphous, molecularly dispersed, or restricted crystalline states, thereby limiting phase separation, crystal growth, and Ostwald ripening [43]. SLNs may be particularly useful for lipophilic compounds because drug–lipid and lipid–SC interactions can support cutaneous partitioning while reducing uncontrolled crystallization during drying. However, incorporation into a nanocarrier does not guarantee sustained thermodynamic activity or efficient drug release. Particle size, carrier crystallinity, drug loading, release behavior, aggregation, polymorphic conversion, and storage stability must therefore be controlled to ensure reproducible delivery.

Effective precipitation control ultimately requires an integrated strategy rather than reliance on a single excipient. The volatile-to-nonvolatile solvent ratio should regulate the rate of supersaturation generation; antinucleant polymers should delay nucleation and crystal growth; CDs and surfactants should buffer drug solubility without excessively decreasing free-drug activity; and lipidic or nanoparticulate systems should restrict phase separation while maintaining adequate drug release. These mechanisms should be evaluated in the complete transformed formulation because an intervention that improves physical stability may simultaneously reduce the thermodynamic driving force for skin partitioning. A successful formulation should therefore maintain a clinically useful supersaturated window, preserve drug availability, and minimize crystallization-related variability throughout the intended application period [44].

## 6. Strategies to Enhance Skin Permeability

### 6.1. Chemical Permeation Enhancers

#### 6.1.1. Mechanistic Basis of Chemical Enhancement

**Figure 5:**
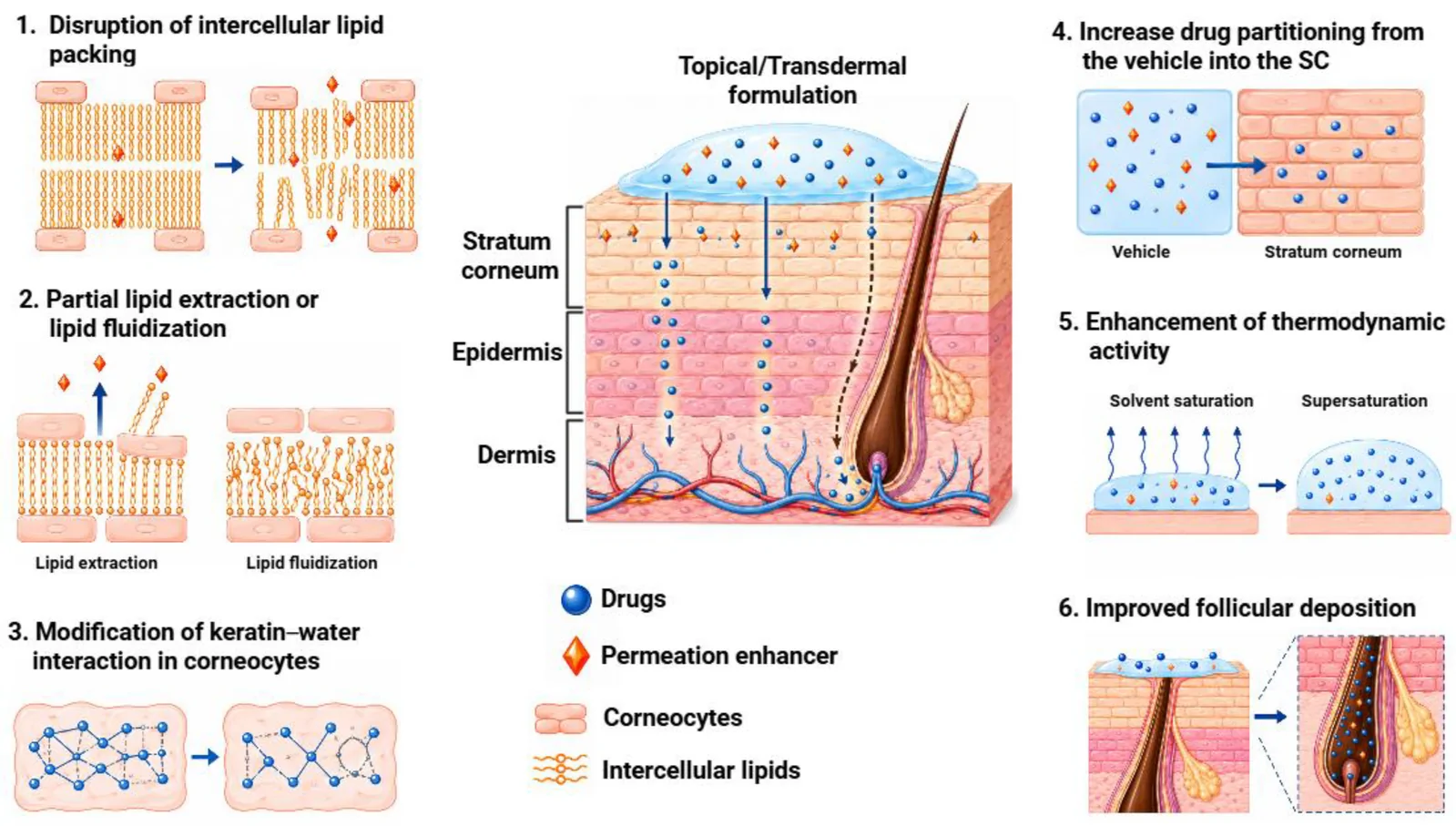
Schematic representation of the chemical permeation enhancement mechanisms in topical and transdermal drug delivery. Chemical permeation enhancers improve skin permeability through multiple mechanisms, including (1) the disruption of intercellular lipid packing in the stratum corneum, (2) partial lipid extraction or lipid fluidization, (3) modification of keratin–water interactions within corneocytes, (4) increased drug partitioning from the vehicle into the stratum corneum, (5) enhancement of thermodynamic activity through solvent evaporation or supersaturation, and (6) improved follicular deposition. These mechanisms may occur individually or synergistically depending on enhancer type, concentration, vehicle composition, and drug physicochemical properties.

Transient modulation of the SC barrier is often required to improve the topical or transdermal delivery of drugs with insufficient passive permeability [50]. CPEs are formulation excipients intended to increase drug transport by producing controlled and preferably reversible changes in the barrier properties of the SC. Their effects occur primarily within the intercellular lipid lamellae and keratin-rich corneocyte domains that collectively determine diffusional resistance (Figure 5). The performance of CPEs is governed by structure–activity relationships, hydrophilic–lipophilic balance, alkyl chain length and branching, degree of saturation, polarity, functional groups, concentration, and exposure duration (Figures S1 and S2) [59]. These properties influence the ability of an enhancer to partition into the SC and interact with its lipid or protein components. CPEs can increase skin permeability through several complementary mechanisms, including disruption of intercellular lipid packing, increased lipid fluidity, partial lipid extraction, modification of keratin–water interactions, and alteration of drug partitioning between the vehicle and the SC [60]. Solvent-type enhancers may additionally modify drug solubility, free drug availability, and thermodynamic activity through drug–vehicle interactions and post-application vehicle transformation. These effects are not uniformly beneficial: excessive drug solubilization within the vehicle may reduce partitioning into the skin, whereas uncontrolled lipid extraction or protein disruption may impair barrier recovery and increase irritation. The net enhancement effect therefore depends on the drug, enhancer concentration, vehicle composition, application conditions, and skin model rather than on enhancer identity alone.

Although CPEs are commonly classified according to chemical structure or predominant mechanism, substantial mechanistic overlap exists among the major classes. Alcohols, glycols, and ether alcohols mainly act as solvents, co-solvents, humectants, and modulators of drug activity, whereas fatty acids, esters, amides, and terpenes interact predominantly with SC lipid domains. Surfactants can affect both lipid and protein organization while also modifying wetting and drug solubilization, and polar aprotic compounds such as pyrrolidones and sulfoxides can alter both drug–vehicle interactions and barrier structure. CPEs are therefore widely incorporated into conventional and advanced topical and transdermal dosage forms, including creams, lotions, gels, suspensions, solutions, emulsions, and vesicular systems such as liposomes, ethosomes, transfersomes, and niosomes (Figure S3). Overall, chemical enhancement generally results from multiple concurrent and formulation-dependent mechanisms rather than from a single mode of action (Figure 5).

#### 6.1.2. Major Enhancer Classes and Applications

Alcohols remain among the most widely used chemical permeation enhancers because they enable drug solubilization while also facilitating SC lipid modulation and post-application vehicle transformation. Ethanol, the most extensively studied representative, can increase drug partitioning into the SC by disrupting lipid organization, increasing lipid mobility, and enhancing thermodynamic activity following solvent evaporation [61,62,63]. Importantly, ethanol-containing systems may lead to transient supersaturation after application, thereby increasing the driving force for skin permeation. Representative examples include bimatoprost formulations showing a 4.6-fold increase in human skin flux and film-forming curcumin systems exhibiting approximately 14-fold higher permeation than conventional formulations [64,65]. However, excessive ethanol concentrations may induce SC dehydration, increased TEWL, irritation, and barrier disruption [63]. Glycols, such as PG and PEG, are frequently employed as co-solvents and humectants. PG improves drug solvation and moderately alters SC lipid organization, particularly benefiting hydrophilic or poorly permeable drugs [66,67,68]. In contrast, PEGs generally function as solubilizers, plasticizers, and release modifiers rather than strong intrinsic enhancers [69,70,71]. Their effectiveness depends strongly on the vehicle composition and drug properties, since excessive drug–vehicle interactions may reduce drug release and subsequent skin partitioning [66,67]. Ether alcohols, Transcutol^®^ (DEGEE), represent a distinct class of enhancer that primarily improves drug solubility and skin partitioning without extensive lipid disruption [72,73,74]. Transcutol^®^ has demonstrated substantial enhancement of drug deposition in the SC, epidermis, and dermis while simultaneously reducing precipitation following the evaporation of volatile solvents [74,75]. Consequently, alcohols, glycols, and ether alcohols are particularly beneficial in formulations where maintaining post-application drug activity and preventing crystallization are critical.

Fatty acids are structurally related to endogenous SC lipids and therefore interact efficiently with intercellular lipid domains [76,77,78]. Unsaturated fatty acids, particularly oleic acid, are generally more effective than saturated analogs because their cis-double bonds disrupt ordered lipid packing and increase lipid fluidity [79]. Oleic acid has been reported to increase olanzapine permeation by approximately 3.3-fold and significantly enhance clozapine flux from matrix-type patches [80,81]. Nevertheless, concentration-dependent irritation, oxidation instability, and variability in enhancement remain important considerations [82,83]. Fatty acid esters such as IPM and isopropyl palmitate enhance permeation by increasing lipid mobility and promoting drug partitioning into SC lipids [84,85]. IPM increased testosterone flux from Carbopol gel by approximately 11-fold when combined with ethanol, illustrating the importance of synergistic enhancement strategies [86]. Likewise, sucrose fatty acid esters improve drug release, interfacial partitioning, and skin wetting through mechanisms resembling those of mild surfactants [87,88,89]. Amide-based enhancers, including Azone and amino-acid-derived derivatives, partition into SC lipid domains and alter lipid organization [90]. Among these, N-acetyl-L-proline dodecyl ester (L-Pro2) demonstrated particularly strong activity, increasing theophylline flux by approximately 40-fold and hydrocortisone permeability by approximately 31-fold through lipid-selective modification of the SC [91]. However, enhancement remains highly dependent on drug properties, formulation composition, and exposure conditions, and excessive concentrations may increase irritation and barrier perturbation [92,93].

Surfactants enhance delivery through a combination of wetting, drug solubilization, lipid fluidization, and protein interaction. Anionic surfactants such as SLS are among the most potent but also the most disruptive to barrier integrity. For example, 5% SLS increased diazepam flux by approximately 9.8-fold across excised rat skin [94]. Nonionic surfactants generally produce milder enhancement while offering improved long-term tolerability and are therefore widely used in topical and transdermal systems [95,96]. Pyrrolidones and sulfoxides are potent polar aprotic enhancers. N-methyl-2-pyrrolidone (NMP) has been reported to increase ibuprofen flux by approximately 16-fold and substantially improve the permeation of several anti-inflammatory drugs through drug–enhancer complex formation and facilitated transport [70,97,98]. Dimethyl sulfoxide (DMSO) remains one of the most powerful chemical enhancers due to its ability to disrupt SC lipids, modify protein structure, and increase drug partitioning [99]. Nevertheless, its odor, systemic absorption, and tendency to cause irritation and burning sensations limit its widespread application. Terpenes are naturally derived enhancers that improve permeation primarily by modifying SC lipid organization and enhancing drug thermodynamic activity [100,101]. Depending on terpene structure, reported enhancement varies widely and may reach approximately 20–90-fold under selected experimental conditions [102,103]. However, volatility, oxidation, sensitization, and formulation variability continue to present translational challenges [104,105,106].

#### 6.1.3. Translational Considerations and Limitations of Chemical Permeation Enhancers

The translational value of a CPE should be determined by whether it provides clinically meaningful drug delivery without causing unacceptable or persistent alteration of the skin barrier, rather than by the enhancement ratio alone. A large fold increase may arise from an extremely low control flux, infinite-dose conditions, excessive occlusion, nonphysiological enhancer exposure, or irreversible barrier damage. The relevant endpoint also depends on the intended product: for a locally acting topical product, the CPE should increase drug availability in the target cutaneous compartment, such as the viable epidermis, dermis, or pilosebaceous unit, while minimizing systemic escape, whereas a systemic transdermal product must provide an absolute and sufficiently sustained delivery rate over a clinically acceptable application area and wear period. CPE candidates should therefore be compared using absolute flux, cumulative permeation, lag time, delivery efficiency, drug retention in individual skin layers, and systemic exposure potential under finite-dose conditions that reproduce the proposed dose, contact time, occlusion, application area, and dosing frequency. Promising candidates should be evaluated in excised human skin from multiple donors before formulation selection is finalized because artificial membranes, reconstructed epidermis, and animal skin cannot fully reproduce human SC lipid organization, appendageal pathways, metabolism, anatomical variability, or barrier recovery. A major translational requirement is to distinguish reversible enhancement from nonspecific injury using complementary measurements such as TEWL, electrical impedance, histology, keratinocyte viability, inflammatory mediators, erythema, edema, and sensitization, obtained before exposure, immediately after formulation removal, and during a predefined recovery period. Repeat-application studies should reproduce the intended dosing interval and determine whether barrier function recovers before the next dose. In ex vivo human skin, 0.75% oleic acid accelerated the early permeation of diclofenac diethylamine but adversely affected TEWL and electrical impedance, whereas oleyl alcohol produced greater epidermal and dermal drug retention with less barrier disruption, demonstrating that the CPE producing the fastest initial permeation was not necessarily the preferable candidate for localized therapy [107]. Selected biodegradable terpene-amino acid esters also substantially enhanced the delivery of theophylline and hydrocortisone through human skin while permitting the recovery of barrier function within 24 h after enhancer removal [108]. More recently, CPE-integrated solid-in-oil dispersions produced transient increases in TEWL without detectable histological damage and enhanced transcutaneous vaccination in mice [109]; however, murine efficacy and short-term histology remain preclinical evidence and cannot replace repeated-dose human-skin or clinical tolerability studies. CPE activity is also specific to the complete drug–CPE–vehicle system because the nominal enhancer concentration does not necessarily reflect its release, thermodynamic activity, or partitioning at the formulation–skin interface. A solvent may increase drug solubility but reduce drug partitioning into the skin, whereas a potent CPE may be ineffective when it is strongly retained in a polymeric or adhesive matrix. In a 2026 study of aminophylline and theophylline transdermal systems, geraniol increased permeation primarily through interaction with SC lipids, whereas Transcutol P increased drug solubility and contributed to cutaneous reservoir formation but showed limited enhancement because of its slow release from the silicone matrix, confirming that matrix thickness, hydration, drug solid state, and release properties can be as important as CPE identity [110]. Similarly, CPE combinations should be regarded as synergistic only when they outperform matched single-enhancer controls at equivalent concentrations and produce an acceptable safety profile; high flux from a multicomponent formulation alone does not demonstrate synergy.

Translation also requires the CPE concentration and exposure to remain controllable during manufacture, storage, and clinical use. Volatile enhancers such as ethanol and some terpenes may be lost during processing, storage, or application, whereas evaporation of the vehicle may progressively concentrate nonvolatile CPEs and alter enhancer partitioning, drug thermodynamic activity, supersaturation, or crystallization. Processing conditions can further modify enhancer retention and release; for example, a 2026 comparison of menthol-containing polyvinyl alcohol films showed that 3D printing, solvent casting, and electrospinning generated different film microstructures, menthol entrapment, six-month retention, release profiles, and epidermal delivery [111]. The final product should therefore be evaluated for CPE content and distribution, residual solvent, drug solid state, release and permeation performance, and batch-to-batch reproducibility throughout stability testing. For transdermal patches, CPE migration into the release liner, backing layer, membrane, or pressure-sensitive adhesive should also be examined because it may alter drug delivery, tack, peel strength, and cohesive stability, while container-closure permeability and package headspace require particular control for volatile enhancers. A history of pharmaceutical or dermal use can support CPE selection but does not establish safety at a higher concentration, over a larger application area, under occlusion, or during chronic administration. Exposure to both the enhancer and the drug should be considered, particularly in pediatric or elderly patients and in atopic dermatitis, psoriasis, wounds, or inflamed skin, where barrier impairment may increase local and systemic absorption relative to healthy-skin models. Clinical experience confirms that CPE translation is possible when enhancement, efficacy, tolerability, and product usability are demonstrated together. A DMSO-containing diclofenac topical solution improved pain and physical function in patients with knee osteoarthritis in a 12-week randomized trial, linking a CPE-containing vehicle to clinical benefit, although application-site dryness and other local reactions demonstrated the tolerability cost of repeated CPE exposure [112]. For systemic delivery, an ethanol/octisalate-based metered-dose estradiol spray produced controllable steady-state estradiol exposure in postmenopausal women and significantly reduced the frequency and severity of vasomotor symptoms in a randomized placebo-controlled trial [113,114]. These examples demonstrate that clinical translation is established by the alignment of CPE concentration, dosage-form design, application area, human exposure, efficacy, local tolerability, and patient use rather than by maximization of ex vivo flux. Accordingly, a CPE should advance only when it (i) achieves the predefined local or systemic delivery target under clinically representative finite-dose conditions; (ii) demonstrates reversible barrier modulation and acceptable repeat-dose tolerability; (iii) produces reproducible performance across human skin donors and relevant application sites; (iv) remains quantitatively and functionally controlled during manufacture, storage, and use; and (v) has an acceptable benefit–risk and excipient-exposure justification for the intended application area, dosing duration, disease condition, and patient population. These CPE-specific criteria connect mechanistic barrier modulation to a clinically developable topical or transdermal product, and the principal mechanisms and translational trade-offs of the major CPE classes are summarized in Table 2.

### 6.2. Vesicular and Lipid-Based Delivery Systems

**Figure 6:**
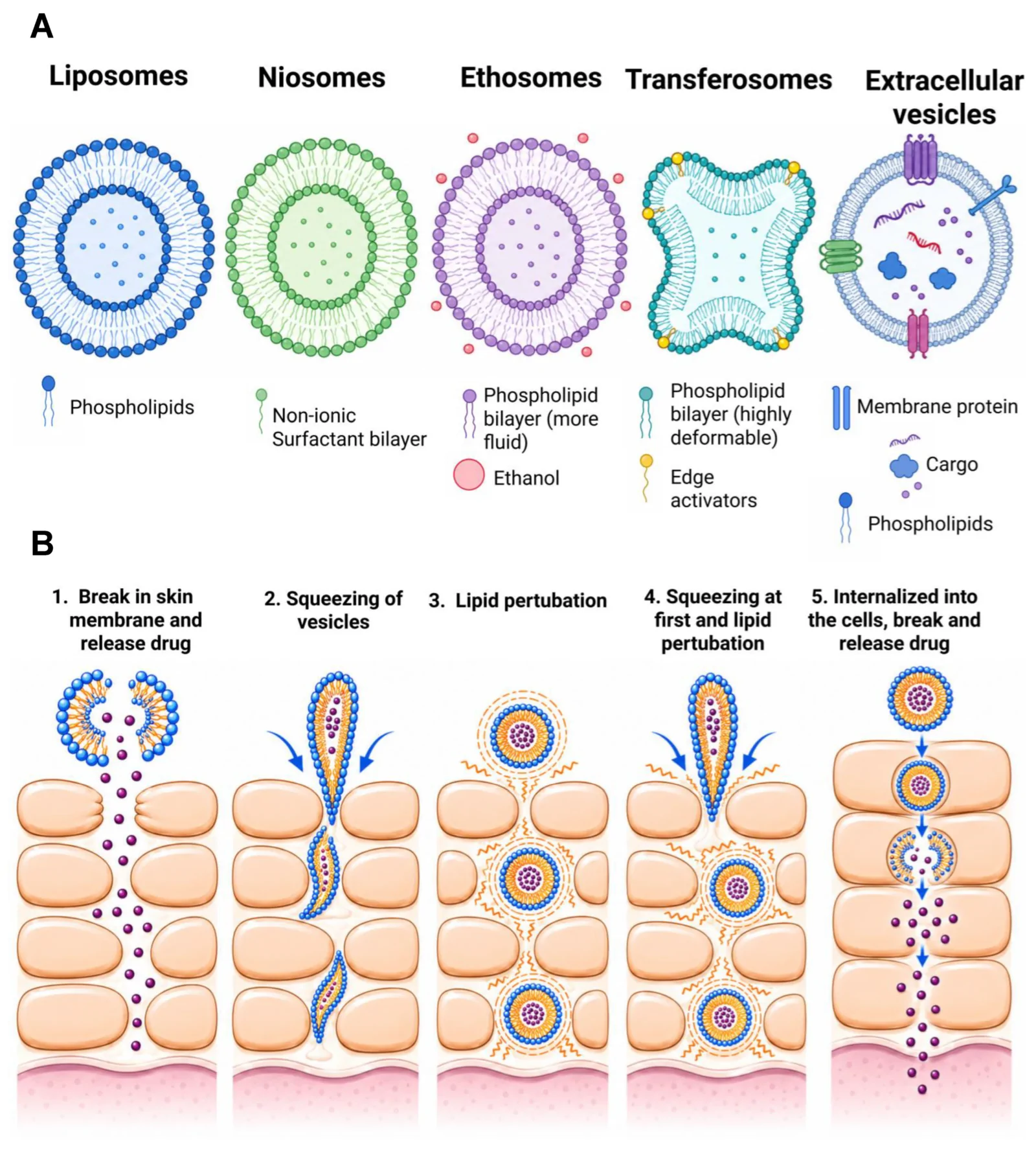
Representative vesicular systems and their potential mechanisms in topical and transdermal drug delivery. (**A**) Representative vesicular carriers, including liposomes, niosomes, ethosomes, transferosomes, and extracellular vesicles, together with their principal structural components. (**B**) Potential delivery mechanisms include drug release near the skin surface, interactions with stratum corneum lipids, deformation of highly flexible vesicles within limited intercellular spaces, and cellular uptake after the payload reaches viable cutaneous tissues. The relative contribution of each mechanism depends on the vesicle composition, payload, formulation, and experimental model.

Vesicular and lipid-based delivery systems are compositionally and mechanistically heterogeneous and should not be interpreted as a single carrier class. In this section, conventional liposomes, transfersomes, ethosomes, ufasomes, niosomes, and glycerosomes are classified as bilayer vesicles; nanoemulsions, SLNs, and nanostructured lipid carriers (NLCs) as non-vesicular lipid dispersions; phytosomes and pharmacosomes as drug–lipid complexes; and extracellular vesicles (EVs) and tiered-release vesicles (TRVs) as specialized biological or engineered vesicles. Depending on their composition, these systems may increase apparent drug solubility, protect unstable payloads, modify drug release and thermodynamic activity, improve formulation–skin contact, hydrate or partially occlude the SC, exchange lipid or surfactant components with SC lipids, and promote follicular or intercellular deposition (Figure 6 and Figure S4) [116,117,118]. Enhanced skin delivery, however, does not demonstrate that intact carriers crossed the SC, because similar results can arise from carrier disruption at the skin surface, release and subsequent partitioning of free drug, excipient-mediated lipid perturbation, or preferential follicular accumulation. The relevant endpoint must therefore be defined by product intent: receptor-phase flux is important for systemic transdermal delivery, whereas epidermal or dermal retention with limited receptor-phase permeation may be preferable for topical therapy. Table 3 consequently includes at least one representative case study for every delivery system discussed in this section, with additional cases retained for selected established systems, and reports the experimental model, comparator, principal outcome, and major evidence limitation.

Representative bilayer-vesicle studies illustrate both the delivery potential and limits of the available evidence. In a 2026 conventional-liposome study, a naringenin formulation produced a steady-state flux of 0.0243 ± 0.004 mg/cm^2^/h across excised rat abdominal skin compared with 0.0002 ± 0.00001 mg/cm^2^/h from a saturated naringenin solution [119]. Although the corresponding enhancement ratio was 121.5, this value was magnified by the near-zero control flux, and the hydroalcoholic receptor phase and animal skin model limit direct clinical extrapolation. C-phycocyanin-loaded enriched transfersomes increased epidermal accumulation relative to non-vesicular C-phycocyanin preparations [120]; a 2026 follow-up study in a 7,12-dimethylbenz[a]anthracene/12-O-tetradecanoylphorbol-13-acetate-induced mouse model further demonstrated reductions in epidermal thickness, rete ridge depth, mast-cell density, mitotic activity, dysplasia severity, and suprabasal Ki-67 expression [121]. This progression from deposition to pharmacodynamic activity strengthens the case for transfersomal delivery but does not establish efficacy in human skin disease. For ethosomes, a cryptotanshinone-loaded gel produced approximately 2.5-fold greater transdermal flux and 2.1-fold greater skin deposition than a compositionally comparable hydroethanolic gel across excised porcine skin and showed improved anti-acne activity with only slight irritation in a rabbit model [122]. Oleic-acid ufasomes increased methotrexate permeation approximately three- to fourfold relative to a plain drug solution or Carbopol gel and retained up to 50% of the administered dose in rat skin, supporting localized delivery but not systemic transdermal exposure [123]. A diclofenac niosomal hydrogel generated an ex vivo rat-skin flux of 0.1017 mg/cm^2^/h, compared with 0.0034 mg/cm^2^/h from a conventional hydrogel and 0.0218 mg/cm^2^/h from Voltaren^®^ Emulgel, and also increased the drug concentrations in rat muscle beneath the application site; nevertheless, the formulation has not been compared using human skin or in a clinical study [124]. Finally, rutin-loaded glycerosomes produced sustained release and higher epidermal and dermal drug concentrations than conventional rutin preparations in excised rat skin, with preferential epidermal retention, antioxidant activity, and measurable sun-protection activity [125]. Because that study did not include a composition-matched conventional-liposome control or a clinical photoprotection endpoint, the individual contributions of glycerol, phospholipid vesicles, and the gel vehicle cannot be distinguished.

The performance of non-vesicular lipid carriers, drug–lipid complexes, and specialized vesicles is similarly dependent on the intended skin compartment and experimental design. A 5-fluorouracil nanoemulsion gel produced a rat-skin steady-state flux of 12.0244 ± 1.12 μg/cm^2^/h compared with 1.350 ± 0.55 μg/cm^2^/h from an aqueous drug solution, and showed greater cytotoxicity against SK-MEL-5 melanoma cells; however, the substantial differences observed among rat, goat, and cow skin demonstrate the species dependence of the result [126]. A piroxicam SLN patch generated a flux of 17.16 μg/cm^2^/h across rat skin versus 4.6 μg/cm^2^/h from a non-SLN patch, despite releasing less drug over 24 h (66.6% versus 88.01%), indicating that release rate alone does not determine skin transport [127]. In a direct comparison of resveratrol-loaded SLNs and NLCs, the NLCs were smaller and exhibited 18% higher entrapment efficiency, while skin recovery was 1.99 ± 0.17 μg/cm^2^ for the NLCs and 1.55 ± 0.13 μg/cm^2^ for the SLNs; NLCs favored deeper dermal deposition, but neither carrier produced detectable receptor-phase transport, demonstrating improved dermal targeting rather than systemic delivery [128]. An olive-oil-containing quercetin phytosomal nanocomplex increased ex vivo permeation across rabbit skin approximately 1.3-fold relative to an olive-oil/surfactant-free formulation and approximately 1.9-fold relative to the control [129]. Because the optimized formulation simultaneously contained a phospholipid complex, olive oil, and surfactant, the contribution of phytosomal complexation could not be isolated from the effects of the other excipients. An optimized simvastatin–phospholipid pharmacosome showed 86.88% in vitro release, a particle size of 151.6 nm, and acceptable physical characteristics after incorporation into a transdermal patch; however, the absence of a matched ex vivo skin-flux or in vivo pharmacokinetic comparison means that the study demonstrates formulation feasibility rather than proven transdermal superiority [130]. Among the biological carriers, tacrolimus-loaded fibroblast-derived small EVs produced approximately 25% greater dermal drug recovery than free tacrolimus in porcine skin while exhibiting lower receptor-phase flux, a profile consistent with controlled release and dermal targeting rather than maximized systemic passage [131]. These findings were based on triplicate ex vivo experiments and cell-based cytokine assays and therefore require confirmation in an in vivo disease model and human skin. TRVs delivered a fluorescently labeled large peptide approximately two- to fivefold more efficiently into ex vivo human skin than optimized liposomes and increased the penetration of two hyaluronic-acid species approximately three- to thirteenfold relative to a simple gel [132]. These results were based primarily on fluorescence intensity and histological cosmetic endpoints and do not yet establish absolute drug bioavailability or therapeutic efficacy. Collectively, these case studies show that vesicular or lipid-based formulations should be compared using a composition-matched control, a clinically relevant finite dose, an appropriate human skin model, and an endpoint aligned with the intended target compartment. Translation additionally requires the control of particle-size distribution, free versus carrier-associated drug, leakage, aggregation, lipid oxidation or hydrolysis, residual solvent, sterilization, rheology, packaging compatibility, manufacturing scale-up, batch reproducibility, repeated-application safety, and storage stability. A formulation should therefore advance because it reproducibly delivers the required free-drug concentration to the intended human skin compartment—not merely because it produces a high experimental enhancement ratio [133,134].

**Table 3:** Representative case studies of vesicular and lipid-based delivery systems for topical and transdermal applications.

Delivery System	Drug or Payload	Representative Case Study, Principal Outcome, and Evidence Limitation	Reference
Conventional liposomes	Naringenin	Excised rat abdominal skin; saturated naringenin solution as comparator. Steady-state flux was 0.0243 ± 0.004 versus 0.0002 ± 0.00001 mg/cm^2^/h, giving an ER_flux_ of 121.5. The ratio was magnified by the near-zero control flux; a hydroalcoholic receptor phase and animal skin were used.	[119]
Conventional liposomes	Dithranol	Mouse abdominal skin; cream base as comparator. Liposomal dithranol produced a flux of 23.13 μg/cm^2^/h versus 4.10 μg/cm^2^/h from the cream. The vesicles were micrometer-sized, and no in vivo efficacy or repeat-application tolerability was established.	[135]
Transfersomes	C-phycocyanin	Enriched transfersomes increased epidermal C-phycocyanin accumulation ex vivo relative to non-vesicular preparations. In a subsequent 2026 DMBA/TPA mouse study, topical treatment reduced epidermal hyperplasia, rete ridge depth, mast-cell density, mitotic activity, dysplasia severity, and suprabasal Ki-67 expression. Human clinical efficacy remains untested.	[120,121]
Keratin-functionalized transfersomes	Podophyllotoxin	Excised porcine ear skin; podophyllotoxin tincture and unmodified transfersomes as comparators. Skin deposition was 2.31 μg/cm^2^ versus 1.07 and 1.28 μg/cm^2^, respectively, while receptor-phase permeation was reduced, supporting epidermal targeting. No in vivo wart model or clinical study was included.	[136]
Ethosomes	Cryptotanshinone	Excised porcine skin and a rabbit acne model; conventional hydroethanolic gel as comparator. The ethosomal gel increased flux approximately 2.5-fold and skin deposition 2.1-fold and improved anti-acne activity, with slight irritation. Human efficacy and chronic tolerability were not evaluated.	[122]
Ethosomes	Huperzine A	Full-thickness mouse abdominal skin in Franz cells; ordinary gel and cream as comparators. The 24-h cumulative permeated amount was 40.99 ± 4.83 μg/cm^2^ versus 21.49 ± 1.99 and 16.80 ± 1.57 μg/cm^2^, respectively. Evidence was limited to ex vivo animal skin.	[137]
Ufasomes	Methotrexate	Rat skin; plain methotrexate solution and Carbopol gel as comparators. Oleic-acid vesicles increased permeation approximately three- to fourfold and retained up to 50% of the administered dose in the skin. No diseased-skin, human-skin, or in vivo efficacy study was performed.	[123]
Niosomes	Diclofenac	Ex vivo and in vivo rat studies; conventional diclofenac hydrogel and Voltaren^®^ Emulgel as comparators. Niosomal-gel flux was 0.1017 mg/cm^2^/h versus 0.0034 and 0.0218 mg/cm^2^/h, respectively, and underlying muscle exposure increased. Human-skin and clinical confirmation are unavailable.	[124]
Glycerosomes	Rutin hydrate	Excised rat skin; conventional rutin preparation as comparator. The glycerosomal gel produced sustained release, greater epidermal and dermal exposure, preferential epidermal retention, antioxidant activity, and measurable SPF activity. A composition-matched conventional-liposome control and clinical photoprotection endpoint were absent.	[125]
Nanoemulsion gel	5-Fluorouracil	Excised rat, goat, and cow skin; aqueous 5-fluorouracil solution as comparator. Rat-skin flux was 12.0244 ± 1.12 versus 1.350 ± 0.55 μg/cm^2^/h, and SK-MEL-5 cytotoxicity increased. Large interspecies differences and the absence of human-skin or in vivo tumor data limit translation.	[126]
SLNs	Piroxicam	Excised rat skin; non-SLN piroxicam patch as comparator. Flux was 17.16 versus 4.6 μg/cm^2^/h, although 24-h release was lower from the SLN patch (66.6% versus 88.01%). No human-skin permeation or pharmacokinetic study was conducted.	[127]
NLCs	Resveratrol	Excised rat skin; resveratrol-loaded SLNs as the direct comparator. NLCs were smaller, had 18% higher entrapment efficiency, and produced greater total and dermal drug accumulation; neither carrier yielded detectable receptor-phase drug. The result supports local dermal targeting rather than transdermal delivery.	[128]
Phytosomes	Quercetin	Excised rabbit skin; olive-oil/surfactant-free formulation and quercetin control as comparators. Permeation increased approximately 1.3- and 1.9-fold, respectively. The effects of phospholipid complexation, olive oil, and surfactant cannot be separated from the formulation-level comparison.	[129]
Pharmacosomes	Simvastatin	In vitro dissolution and transdermal-patch characterization. The optimized simvastatin–phospholipid formulation showed 86.88% release and a particle size of 151.6 nm. Because no matched skin-flux, systemic pharmacokinetic, or pharmacodynamic comparison was reported, transdermal superiority was not demonstrated.	[130]
Fibroblast-derived small EVs	Tacrolimus	Porcine ear skin; free tacrolimus and commercial ointment as comparators. Tacrolimus-loaded EVs produced approximately 25% greater dermal recovery than free drug but lower receptor-phase flux, indicating dermal targeting and controlled release. The ex vivo and cell-based experiments used three replicates and lacked in vivo efficacy confirmation.	[131]
TRVs	Large peptide and two hyaluronic-acid species	Ex vivo human skin; optimized liposomes or simple gel as comparators. Peptide delivery increased approximately two- to fivefold, and penetration of the two hyaluronic-acid species increased approximately three- to thirteenfold. The study relied principally on fluorescence and histological cosmetic endpoints and did not establish therapeutic bioavailability.	[132]

Note: DMBA, 7,12-dimethylbenz[a]anthracene; ER_flux_, flux enhancement ratio; EVs, extracellular vesicles; NLCs, nanostructured lipid carriers; SC, stratum corneum; SLNs, solid lipid nanoparticles; SPF, sun protection factor; TPA, 12-O-tetradecanoylphorbol-13-acetate; TRVs, tiered-release vesicles. At least one case study is provided for every delivery system discussed in Section 6.2; additional examples are included for selected established vesicular systems. Numerical results should not be compared directly across studies because the applied doses, skin species, receptor media, exposure conditions, and analytical endpoints differed.

### 6.3. Physical Enhancement Technologies

**Figure 7:**
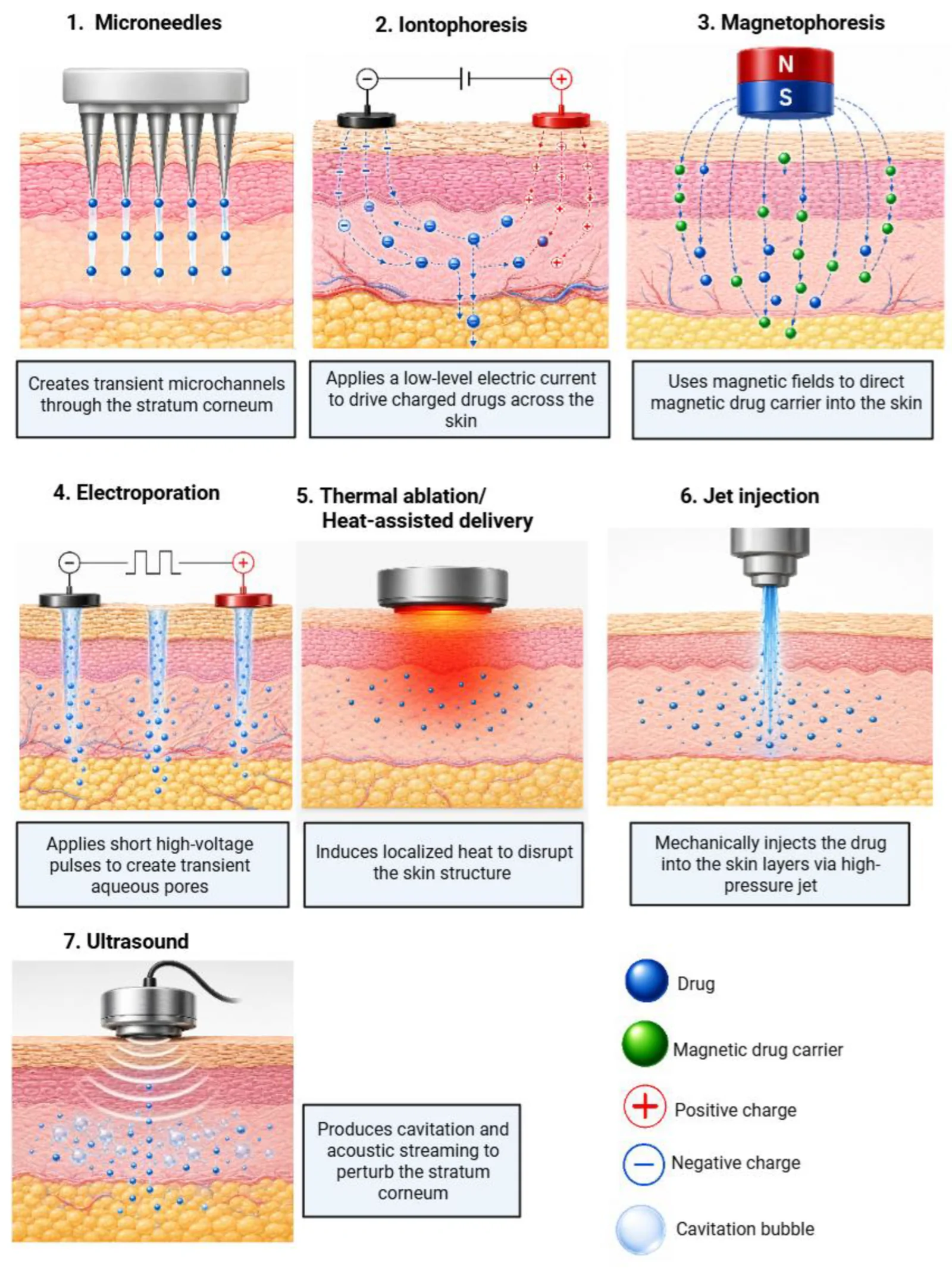
Integrated schematic representation of physical enhancement-based transdermal and intradermal drug delivery strategies. The figure summarizes the major physical methods used to enhance skin permeability, including microneedles, iontophoresis, magnetophoresis, electroporation, thermal ablation/heat-assisted delivery, jet injection, and ultrasound. These techniques enhance delivery by generating transient microchannels, applying electrical or magnetic driving forces, creating temporary aqueous pores, inducing localized thermal disruption, mechanically injecting drug into skin layers, or producing cavitation-mediated stratum corneum perturbation.

Different physical enhancement techniques—including MNs, iontophoresis, magnetophoresis, electroporation, thermal ablation (heat-assisted delivery), jet injection, and ultrasound—are employed to enhance drug transport across the skin (Figure 7). These approaches are particularly effective for hydrophilic small molecules, peptides, proteins, and biologics that exhibit limited passive permeation due to their size and polarity. By transiently disrupting or bypassing the SC, physical techniques enable direct access to the viable epidermis and dermis while minimizing systemic invasiveness [138]. Unlike chemical and vesicular approaches, these techniques rely on external energy or mechanical forces to modulate the skin barrier.

#### 6.3.1. Microneedle-Based Enhancement

**Figure 8:**
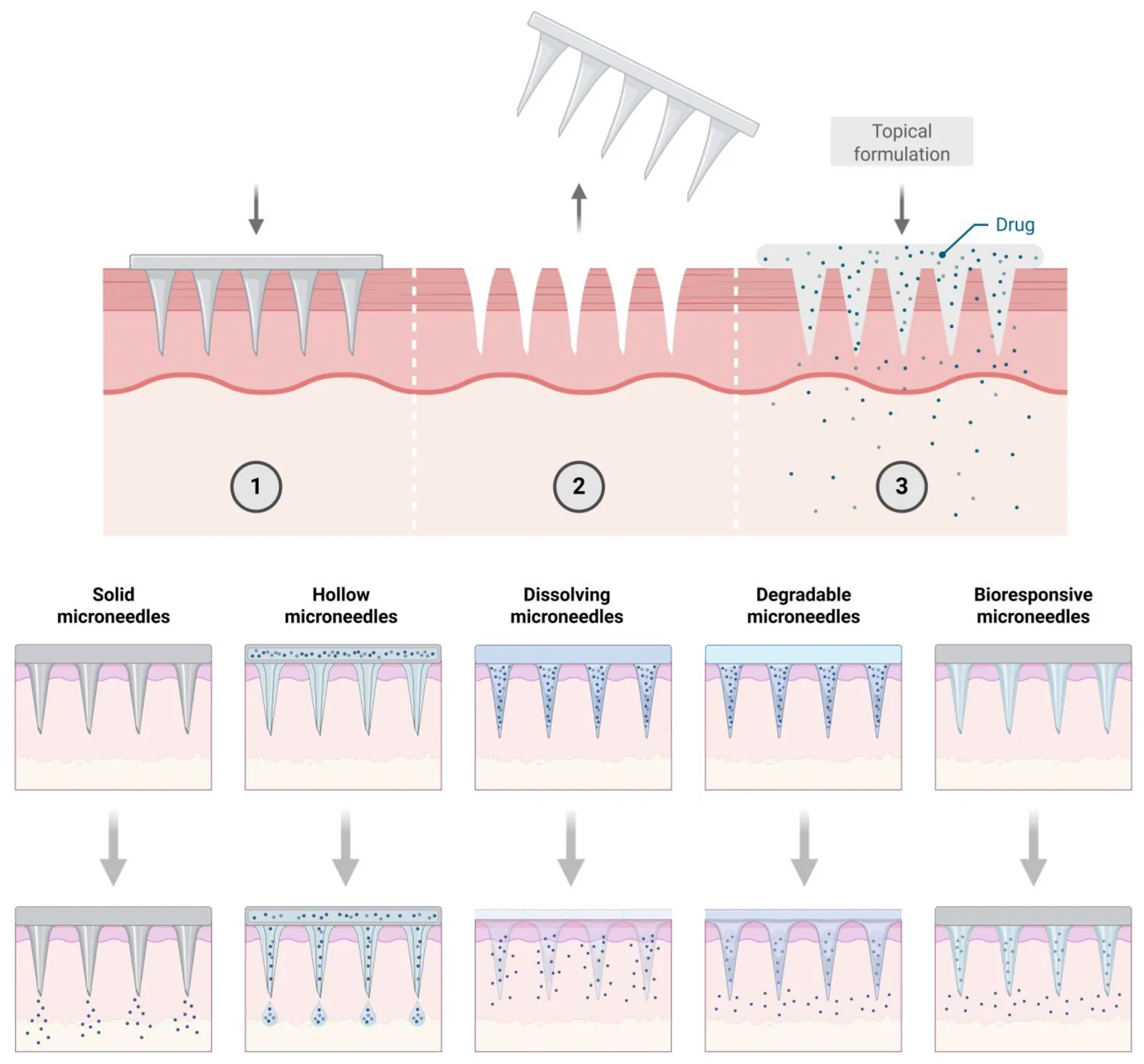
Microneedle-mediated enhancement of topical and transdermal drug delivery. The upper panel illustrates microneedle insertion across the stratum corneum, transient microchannel formation after array removal, and subsequent drug delivery from a topically applied formulation. The lower panel summarizes representative microneedle architectures and their principal drug release patterns. Two recent nanocarrier-integrated microneedle applications are discussed in the accompanying text and summarized in Table 4.

MNs enhance topical and transdermal drug delivery by creating transient microchannels across the SC, thereby providing minimally invasive access to the viable epidermis and dermis (Figure 8). Unlike conventional topical formulations that depend on passive diffusion through the intact SC, MNs physically bypass the principal skin barrier and facilitate the delivery of poorly permeable small molecules, peptides, proteins, vaccines, antibodies, and other biologics [139,140]. Depending on the intended therapeutic objective, MN-mediated delivery can be directed toward local drug deposition within the skin or systemic absorption through the dermal microcirculation.

Recent studies have demonstrated the potential of combining MN-mediated barrier bypass with nanocarrier-based drug delivery. In a 2026 study, benidipine-loaded nanotransfersomes were incorporated into rapidly dissolving MNs. The resulting formulation produced an ex vivo transdermal flux of 5.23 ± 0.64 μg/cm^2^/h compared with 1.64 ± 0.32 μg/cm^2^/h for benidipine-loaded MNs without nanotransfersomes, corresponding to an enhancement ratio of 3.19 ± 0.20. In rats, the hybrid system increased the relative bioavailability by approximately 1.35-fold compared with oral benidipine tablets and 2.49-fold compared with benidipine-loaded MNs without nanotransfersomes [141]. This study demonstrates how the combination of a nanocarrier and MN-mediated barrier disruption can improve both skin permeation and systemic exposure. In another 2026 study, hyaluronic acid-based MNs containing SLNs loaded with 3-acetyl-11-keto-β-boswellic acid (AKBA) were developed for the transdermal treatment of rheumatoid arthritis. The AKBA-loaded MN system produced approximately 4.8-fold higher drug permeation and 3.4-fold higher dermal delivery than an AKBA cream. It also generated higher C_max_ and AUC_0–24 h_ values than orally administered AKBA and improved therapeutic outcomes in an experimental rheumatoid arthritis model [142]. This case illustrates the potential of MNs to improve the cutaneous delivery of poorly soluble compounds while increasing local therapeutic exposure.

These two studies indicate that MN performance depends not only on the creation of microchannels, but also on drug loading, carrier composition, needle geometry, mechanical strength, insertion consistency, dissolution behavior, and the intended site of drug action. Combining MNs with lipid or vesicular nanocarriers may further improve drug solubilization, stability, controlled release, and tissue retention. However, both representative studies remain preclinical, and their results cannot yet be directly extrapolated to human clinical performance. Clinical translation requires reproducible insertion across variable human skin, accurate dose delivery, adequate drug-loading capacity, formulation and device stability, scalable manufacturing, appropriate sterilization, repeated use safety, patient usability, and clear regulatory classification as a drug, device, or combination product [143]. Therefore, future MN development should evaluate not only permeation enhancement, but also human skin performance, manufacturing robustness, dose reproducibility, safety, and clinically relevant therapeutic outcomes. Additional representative applications and their principal outcomes are summarized in Table 4.

**Table 4:** Representative microneedle-assisted skin delivery studies and their major outcomes.

Microneedle Details	Drug	Outcomes	Reference
Solid microneedle roller (173 ± 26.94 µm microchannels)	Potassium chloride (KCl)	Microneedle pretreatment increased the mean KCl flux from 0.637 ± 0.02 to 6.33 ± 18.70 mg/cm^2^/h, corresponding to an approximately 9.9-fold increase.	[144]
3D-printing-assisted bilayer dissolving microneedles (DMNs)	Flurbiprofen	Star-type DMNs achieved 100% penetration and the highest cumulative permeation, 86.9 ± 9.9% in 12 h, compared with pyramidal 77.8 ± 9.0% and conical 64.4 ± 10.2%. The non-microneedle film showed a 2.23 h lag time, whereas all DMN designs showed no clear lag phase; the star-type design produced an approximately 8.5-fold enhancement versus control film.	[145]
Rapidly dissolving hyaluronic acid microneedles	Benidipine hydrochloride	Benidipine rapidly dissolving hyaluronic acid microneedles (BEN-DMNs) almost completely dissolved in rat skin within 8 min, 96.79 ± 1.65%. Ex vivo flux increased to 5.23 ± 0.64 µg/cm^2^/h versus 1.64 ± 0.32 µg/cm^2^/h for BEN-DMNs, giving an enhancement ratio of 3.19 ± 0.20. Permeability coefficient increased to 0.0884 ± 0.0162 cm/h. Relative bioavailability increased 1.35-fold versus oral tablets and 2.49-fold versus BEN-DMNs.	[141]
Lipid nanoparticle-loaded hyaluronic acid dissolving microneedles (LNP-DMNs)	3-Acetyl-11-keto-β-boswellic acid (AKBA)	AKBA-LNP-DMNs produced 4.8-fold higher drug permeation and 3.4-fold higher dermal delivery than AKBA cream. Pharmacokinetic exposure, including C_max_ and AUC_0–24 h_, was higher than oral AKBA. In the arthritis model, the system reduced paw volume, paw thickness, and inflammatory cytokines.	[142]
Hyaluronic acid/aminoclay (Ac/HA10) nanocomplex dissolving microneedles	Semaglutide (SEMA)	Sema-AC/HA10 microneedles reached approximately 400 µm penetration depth in pig cadaver skin. Compared with free drug MNs, aminoclay nanocomplex MNs increased systemic exposure, with 1.74-fold higher C_max_ and 2.74-fold higher AUC for Sema-AC/HA33 versus Sema/HA33. Sema-AC/HA10 produced an efficacy comparable to subcutaneous semaglutide; after 30 days, fasting glucose and glycated hemoglobin A1c (HbA1c) decreased to approximately 62% and 65% of the initial values.	[146]
Dissolved bubble microneedle patch (DBMNP)	Dipotassium glycyrrhizinate (DPG), PIONIN, and salicylic acid	Bubble microneedle patch co-delivered hydrophilic DPG, hydrophobic PIONIN, and salicylic acid with spatially separated loading. Needles almost completely dissolved within 5 min after insertion. Drug-loaded DBMNPs reduced ear swelling, nearly eliminated P. acnes colonies, decreased pro-inflammatory IL-6, increased anti-inflammatory IL-10, and outperformed topical drug solution because of improved transdermal delivery.	[147]
Microneedle patches	Naltrexone	Microneedle pretreatment increased the permeability of naltrexone hydrochloride across guinea pig and human skin in vitro. Permeability across microneedle-treated human skin reached 7.0 × 10^−5^ cm/h, while the lag time in guinea pig skin decreased approximately tenfold. In a separate first-in-human study, microneedle pretreatment enabled measurable plasma naltrexone exposure for approximately 48–72 h.	[148,149]
Silicon microneedle	5-aminolevulinic acid or 5-aminolevulinic acid methyl ester	Drug-induced production of the photosensitizer protoporphyrin IX was greater after delivery combined with skin pretreatment using microneedles than without microneedle treatment in both a human study and one in rats.	[150,151]
Microneedle patches	Phenylephrine (PE)	For rats pretreated with microneedles, topical application of 30% phenylephrine gel rapidly increased the mean resting anal sphincter pressure from 7 ± 2 cmH_2_O to a peak value of 43 ± 17 cmH_2_O after 1 h, which was significantly greater than in rats receiving PE gel without microneedle pretreatment.	[152]
Solid silicon microneedle	Dyclonine	Analysis of the primary endpoint—the time required to achieve a significant degree of pain reduction (13 mm on a 100 mm scale)—revealed that the designed microneedle significantly decreased the time to pain reduction.	[153]
Microneedle roller	Aminolevulenic acid	Clinical improvement at 6 months was judged to be greater than 50% compared to baseline photography in 90% of patients.	[154]
Auxiliary permeable microneedles	Azelaic acid and matrine	The 24 h cumulative permeation of the acidic and alkaline model drugs azelaic acid and matrine reached 51.73 ± 2.61% and 54.02 ± 2.85%, respectively—approximately 10-fold enhancement in their transdermal permeability compared to their free forms.	[155]
Dissolving microarray patches	Ivermectin	The drug underwent quicker deposition and penetrated the skin to a depth of 650 μm from microarray patches fabricated using two-layer casting methods with a surfactant (tween 80 or soluplus) and ivermectin.	[156]

#### 6.3.2. Energy-Assisted Enhancement Technologies

**Figure 9:**
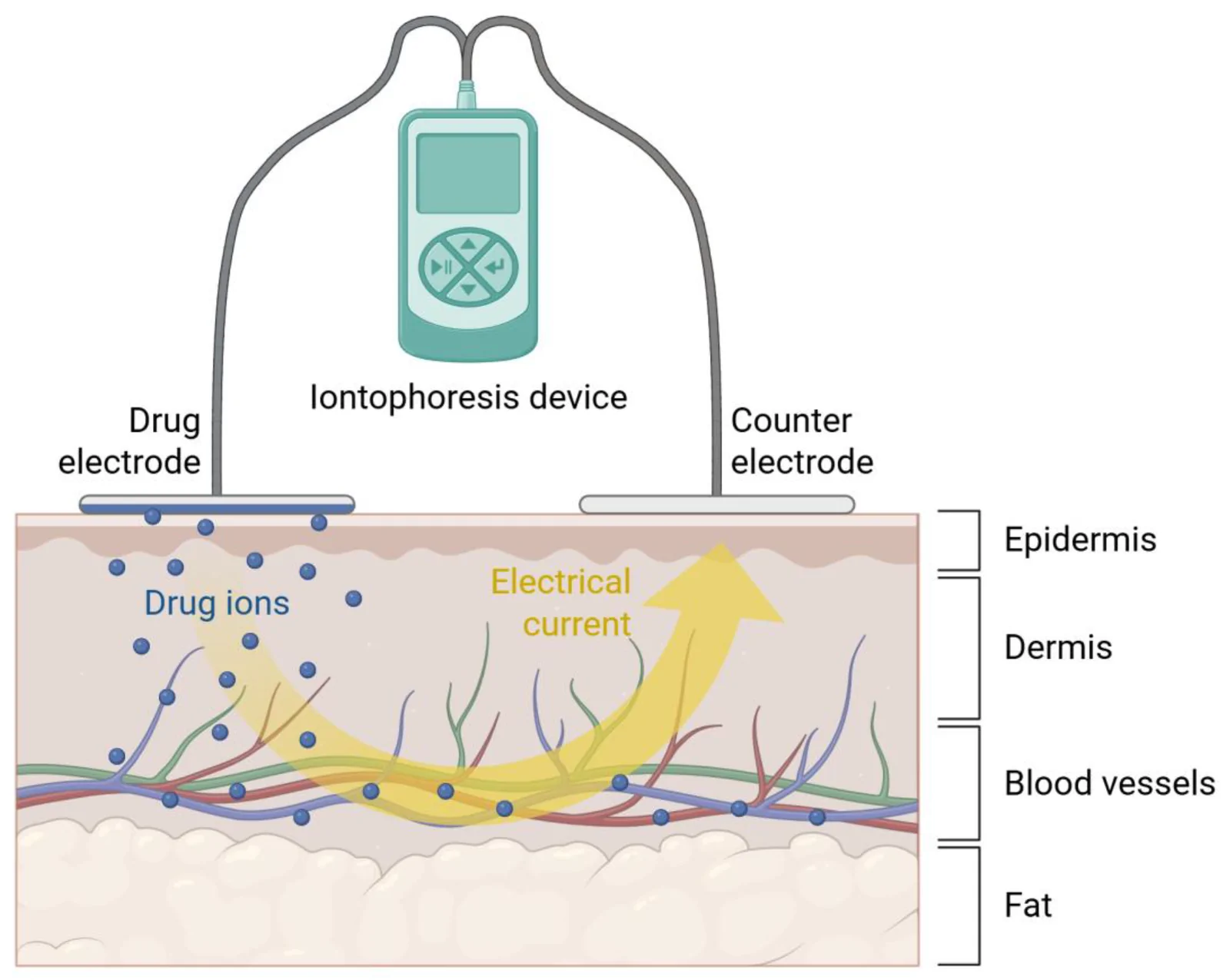
Principle of iontophoresis-mediated transdermal drug delivery. Schematic illustration of an iontophoresis system showing a drug-containing electrode and a counter electrode connected to a current-generating device. Application of a low-intensity electrical current drives charged drug molecules from the donor electrode across the epidermis and dermis via electrorepulsion and electroosmosis, facilitating their transport toward deeper tissues and the dermal microvasculature. Underlying subcutaneous fat is depicted for anatomical context.

Iontophoresis uses a low-intensity direct current, generally 0.1–0.5 mA/cm^2^, to enhance drug transport across the skin. Charged molecules are transported primarily by electrorepulsion, with cationic drugs delivered from the anode and anionic drugs from the cathode, whereas electroosmotic solvent flow through the negatively charged skin matrix can facilitate the transport of neutral or weakly charged compounds (Figure 9). At clinically relevant current densities, disruption of the SC is generally limited and reversible [157,158]. Because drug input can be adjusted by controlling current density and treatment duration, iontophoresis offers more direct dose modulation than passive diffusion, although the relationship between current and flux remains dependent on drug charge, competing ions, formulation conductivity, and skin resistance. Applications include rapid dermal delivery of lidocaine and enhanced delivery of anti-inflammatory drugs such as dexamethasone and ketoprofen [159,160,161], as well as increased local retention of compounds such as tramadol [162] (Table 5). Preclinical studies have further reported iontophoretic delivery of small interfering ribonucleic acid (siRNA) and insulin, indicating potential applicability to selected macromolecules [163,164]. Nevertheless, delivery efficiency decreases with increasing molecular size and is strongly affected by ionization, electrical mobility, and formulation stability. Device dependence, electrode variability, skin irritation or discomfort at higher current densities, and electrochemical degradation of the formulation remain important translational limitations.

Magnetophoresis applies an external magnetic field to increase skin transport without directly ionizing the drug or substantially disrupting the SC. Proposed mechanisms collectively described as magnetokinesis include magnetically induced solvent convection and weak movement of diamagnetic solutes under a magnetic field gradient; follicular and sweat duct pathways may also contribute to transport [176]. The magnitude and direction of enhancement depend on field strength, gradient, frequency, orientation, and drug and vehicle properties. A rotating magnetic field at 50 Hz increased cumulative naproxen permeation across porcine skin from 267.57 ± 41.74 to 1461.40 ± 256.15 μg/cm^2^, corresponding to an approximately 5.5-fold increase [177]. Magnetophoretic enhancement has also been reported for lidocaine, diclofenac, 5-aminolevulinic acid, and naltrexone [176,178]. In another study, cumulative ketoprofen permeation after 8 h increased from 161.88 ± 42.39 μg in the control to 609.62 ± 92.45 μg under a positive static magnetic field, while oscillating and rotating fields also increased permeation or skin accumulation [179]. These findings suggest that magnetic field configuration may influence whether a system favors systemic transport or local dermal retention. However, most evidence remains preclinical, the magnetic driving force is relatively weak compared with electrically assisted methods, and the underlying transport mechanisms have not been established consistently across drugs and experimental models.

Electroporation uses short, high-voltage pulses to transiently generate aqueous pathways within the SC. Drug transport occurs through passive diffusion across the newly formed pathways together with electrophoretic and electroosmotic contributions [180]. Reported operating conditions commonly range from approximately 100 to 1500 V, with pulse durations from microseconds to milliseconds, although the optimal conditions depend on electrode geometry, treated area, skin properties, and payload characteristics. When appropriately controlled, barrier disruption is reversible and skin recovery generally occurs within hours [181,182]. Electroporation is particularly relevant to hydrophilic and charged payloads, including peptides, proteins, deoxyribonucleic acid (DNA), and siRNA, that are otherwise excluded by the SC (Table 6). Its performance is governed by pulse voltage, number, duration and spacing, as well as drug molecular weight, charge, and intrinsic diffusivity. Combination with MNs can shorten the transport distance, improve electric field distribution, and reduce the voltage required for delivery, whereas integration with chemical enhancers, vesicular carriers, or hydrogel reservoirs may improve loading and maintain contact with the treated skin [183]. Despite its broad payload compatibility, electroporation requires specialized devices and precise parameter control, and excessive electrical exposure may cause discomfort, erythema, irritation, or tissue injury. Translation therefore depends on reproducible pore formation, controlled dose delivery, barrier recovery, and acceptable repeated use safety.

Thermally assisted delivery encompasses ablative and non-ablative approaches that transiently reduce SC resistance. Short, localized high-temperature exposures can vaporize SC water and create micropores while limiting thermal injury to deeper viable tissue [192]. This localized disruption facilitates the transport of hydrophilic drugs, peptides, proteins, and other poorly permeable molecules [193,194]. Ablative fractional lasers, including Er systems, generate spatially controlled microchannels in the SC or superficial epidermis and may be combined with nanocarriers to increase intradermal deposition [193,195,196]. Radiofrequency microporation produces localized ionic heating and has been used to enhance the delivery of hydrophilic compounds and proteins, including growth factors [197,198]. In contrast, non-ablative controlled heating increases drug diffusion, skin permeability, and local blood flow without intentionally removing the SC. Controlled heating has, for example, increased nicotine absorption from transdermal patches under clinically acceptable conditions [195]. Representative applications are summarized in Table 7. The delivery outcome depends on temperature, exposure duration, treated area, energy source, and microchannel depth; insufficient exposure may produce variable enhancement, whereas excessive heating may cause pain, erythema, burns, or irreversible tissue damage. Accurate thermal control and reproducible device–skin contact are therefore essential.

Ultrasound-mediated delivery, also known as sonophoresis or phonophoresis, transiently alters SC structure through frequency-dependent mechanical and thermal effects. Low-frequency ultrasound, typically approximately 20–100 kHz, generally produces the greatest permeability enhancement because inertial cavitation generates localized stress, disrupts SC lipid organization, and forms transient aqueous pathways. These effects can increase the transport of both small molecules and macromolecules, including proteins [208]. Higher-frequency ultrasound generally produces less extensive barrier disruption and acts predominantly through acoustic streaming, localized heating, and convective transport. Delivery efficiency is influenced by frequency, intensity, duty cycle, exposure duration, coupling medium, and skin hydration. Representative applications are summarized in Table 8. Combination with chemical enhancers or ethanol-containing formulations may improve drug solubilization and SC lipid perturbation while ultrasound promotes convective transport. Ultrasound has also been investigated for transcutaneous immunization, rapid local anesthesia, and the enhanced delivery of hydrophilic or high-molecular-weight compounds. However, specialized equipment, variable coupling conditions, treatment-related heating, discomfort, and interindividual differences in skin response continue to limit reproducibility. Parameter optimization must therefore balance permeability enhancement against barrier recovery and tissue tolerability [208].

Collectively, energy-assisted technologies can expand topical and transdermal delivery beyond the physicochemical range accessible through passive diffusion, but their mechanisms, payload suitability, and translational readiness differ substantially. Iontophoresis provides current-controlled transport primarily for charged compounds; magnetophoresis remains a largely experimental field-driven approach; electroporation enables rapid aqueous-pathway formation for hydrophilic molecules and macromolecules; thermal systems create spatially controlled barrier defects; and ultrasound combines cavitation, mechanical effects, and convective transport. Clinical development should therefore be based on absolute delivered dose, device reproducibility, barrier recovery, repeated use safety, patient acceptability, and therapeutic advantage rather than on the experimental enhancement ratio alone.

#### 6.3.3. Jet Injection-Based Delivery

Jet injectors enable needle-free drug administration by delivering a high-velocity liquid or powder stream across the SC into intradermal, subcutaneous, or intramuscular tissue. Unlike conventional needle-based injection, these systems rely on high jet velocity and micron-scale nozzle diameters to penetrate the skin and deposit drugs directly into targeted tissue compartments [217]. Drug delivery via jet injection generally occurs through two sequential stages: an initial puncture phase, in which the jet creates a transient microchannel in the skin, followed by a dispersion phase, in which the formulation spreads within the underlying tissue. The efficiency and reproducibility of jet injection are governed by both device-related and formulation-related parameters. Jet velocity, driving pressure, nozzle diameter, nozzle–skin distance, injection volume, and application angle influence penetration depth and deposition geometry, whereas formulation properties such as viscosity, surface tension, particle size, and rheological behavior affect jet stability and tissue dispersion [218,219]. These parameters collectively determine whether the formulation remains within the dermis, reaches the subcutaneous compartment, or penetrates deeper tissues. Representative factors affecting penetration depth and drug distribution are summarized in Table 9.

Jet injectors are typically powered by spring or compressed gas mechanisms and have been widely investigated for the delivery of vaccines, insulin, and other macromolecules. In addition to liquid systems, gas-driven platforms have been adapted for powder delivery, allowing the controlled deposition of micron-sized particles into the dermis [224]. Advances in device design, including disposable or single-use cartridges, have reduced cross-contamination risks and improved safety profiles. Clinical studies have reported immunogenic responses comparable to those achieved with conventional needle-based injections, although transient local reactions such as erythema, swelling, bruising, or discomfort may occur [225,226]. Emerging laser-actuated jet injectors provide more precise control over jet dynamics by using cavitation-driven microjets. This approach enables rapid and localized delivery while potentially reducing mechanical variability and preserving the stability of sensitive biomolecules [227,228]. Such systems may be particularly useful for biologics or vaccines that require controlled deposition depth and minimal formulation stress. Overall, jet injectors provide a rapid and effective physical enhancement strategy for needle-free transdermal and intradermal delivery, particularly for vaccines, insulin, biologics, and other macromolecules. However, broader clinical application remains limited by device complexity, cost, variability in penetration depth, dose–volume constraints, potential discomfort, and the need for precise matching between device parameters and formulation properties. Therefore, the optimization of jet dynamics, nozzle design, formulation rheology, and administration protocol is essential for achieving reproducible and clinically acceptable delivery.

## 7. Comparative Analysis of Enhancement Strategies

**Figure 10:**
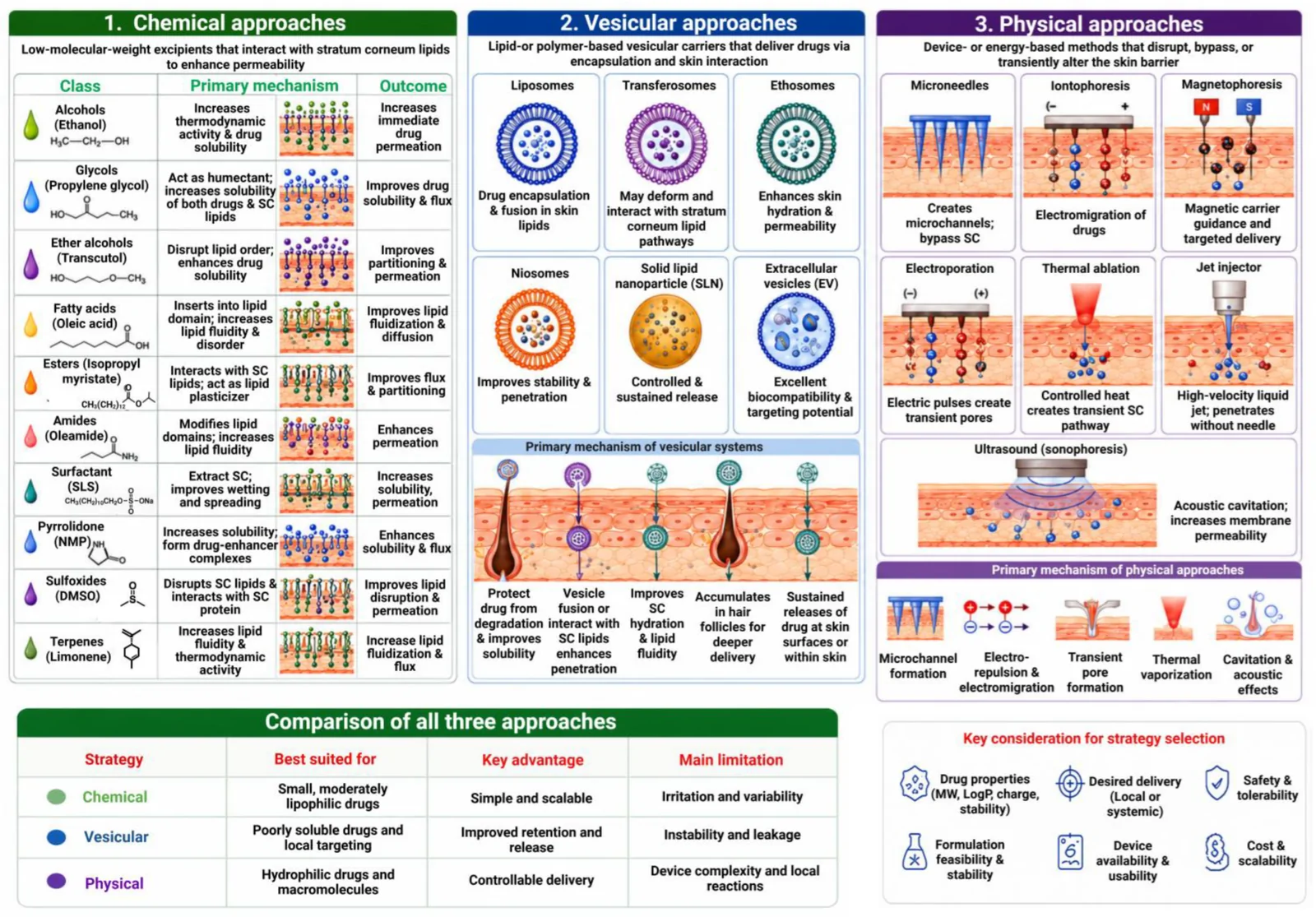
Comparative overview of chemical enhancers, vesicular systems, and physical enhancement technologies for topical and transdermal drug delivery. The figure illustrates their representative mechanisms and summarizes suitable drug characteristics, key advantages, major limitations, and practical considerations for strategy selection.

CPEs, vesicular systems, supersaturation-based formulations, and physical enhancement technologies improve skin delivery through fundamentally different mechanisms. CPEs alter the barrier properties of the SC by disrupting or fluidizing intercellular lipids, modifying keratin–water interactions, and increasing drug partitioning into the skin. Vesicular systems primarily improve drug solubilization, release, local retention, and follicular deposition, while deformable vesicles may additionally interact with or penetrate through SC lipid pathways depending on their composition. In contrast, supersaturation-based formulations increase the thermodynamic activity and chemical potential of a drug at the surface of the skin without necessarily disrupting the SC. Physical technologies use mechanical, electrical, thermal, acoustic, or high-velocity forces to create or drive transport across transient pathways in the barrier. Figure 10 summarizes the principal mechanisms of the chemical, vesicular, and physical approaches. Supersaturation-based systems, which increase thermodynamic activity without primarily altering the skin barrier, are discussed separately in the text. A practical strategy selection framework based on drug properties, target tissue, and delivery requirements is provided in Table 10. Across these platforms, several findings are consistently supported. CPEs generally increase the delivery of small molecules by modifying SC lipids, drug partitioning, or thermodynamic activity, although the magnitude of enhancement varies substantially with drug properties, enhancer concentration, vehicle composition, and skin model. Supersaturated formulations consistently provide a greater transient driving force for permeation, but this advantage is maintained only when crystallization is sufficiently delayed. Vesicular and lipid-based carriers reproducibly improve drug solubilization, skin surface residence, and cutaneous deposition, whereas physical approaches such as MNs, iontophoresis, electroporation, ultrasound, and thermal ablation consistently reduce or bypass the diffusional resistance of the SC under appropriately controlled conditions.

Important controversies and conflicting findings nevertheless remain. For vesicular systems, increased drug deposition is frequently reported, but evidence that intact vesicles penetrate deeply through the SC remains inconsistent. Depending on vesicle composition, deformability, labeling method, and skin model, the observed benefit may result from intact vesicle transport, follicular accumulation, lipid exchange with the SC, or release of the drug at the skin surface. Similarly, chemical enhancers and supersaturated systems often produce large increases in vitro or ex vivo, whereas the magnitude of enhancement is generally smaller or more variable in human skin because of differences in barrier integrity, hydration, application dose, solvent evaporation, and crystallization behavior. For physical technologies, barrier bypass and increased delivery are consistently demonstrated experimentally, but clinical outcomes remain inconsistent when device-only effects, occlusion, tissue remodeling, or formulation effects are not separated using appropriate controls. Thus, the principal cross-platform conclusion is not that one strategy is universally superior, but that experimental enhancement is highly context-dependent. The most reliable evidence is obtained when increased delivery is reproduced in human skin and linked to target tissue exposure, pharmacokinetics, clinical benefit, barrier recovery, and appropriate vehicle- or device-controlled comparisons.

CPEs are particularly suitable for small molecules that possess moderate lipophilicity but exhibit insufficient passive flux. They are relatively inexpensive, readily incorporated into conventional dosage forms, and generally compatible with established manufacturing processes [6,104]. Their effectiveness, however, depends strongly on enhancer concentration, drug solubility, ionization, and drug–vehicle–skin partitioning. Excessive or prolonged barrier perturbation may increase TEWL, irritation, sensitization, and formulation instability. CPEs therefore offer a practical approach for conventional topical and transdermal products but remain less effective for highly hydrophilic compounds and macromolecules. Supersaturation-based systems increase skin permeation by raising the free drug activity and concentration gradient at the formulation–skin interface [9,10]. They are particularly useful in evaporating vehicles and film-forming systems, in which solvent loss after application can transiently increase the saturation ratio. Unlike CPEs, their primary effect is to increase the thermodynamic driving force rather than directly disrupt the skin barrier. Their performance is nevertheless limited by nucleation and crystallization, which can rapidly reduce the dissolved drug fraction and produce variable delivery. Effective supersaturated formulations therefore require controlled solvent evaporation and appropriate crystallization inhibitors.

Vesicular systems, including liposomes, niosomes, transfersomes, and ethosomes, can accommodate drugs with different solubility profiles and improve local delivery through controlled release, SC lipid interactions, hydration, and follicular deposition [229]. However, improved skin delivery should not be interpreted as evidence that intact vesicles routinely penetrate into the deeper skin. Depending on carrier composition and deformability, their principal contribution may instead involve improved drug solubilization, release at the skin surface, lipid exchange with the SC, or accumulation within hair follicles. Vesicular systems are therefore most useful when solubilization, local retention, follicular targeting, or sustained release is required. Their translation remains limited by drug leakage, aggregation, physical instability, sterilization challenges, scale-up complexity, and batch-to-batch variability [230]. Physical enhancement technologies differ substantially in both mechanism and suitable payload. MNs create discrete microchannels, with delivery governed by needle geometry, insertion depth, drug loading, and dissolution or infusion kinetics. Iontophoresis primarily drives charged and polar molecules by electrorepulsion and electroosmosis, and is controlled by current density, treatment duration, and drug charge. Electroporation forms transient aqueous pores through short high-voltage pulses, whereas ultrasound enhances permeability through cavitation and lipid disorganization governed by frequency, intensity, and exposure time. Thermal or laser ablation removes localized regions of the SC according to the applied energy and ablation depth, while jet injection uses a high-velocity liquid stream to bypass the barrier, with delivery determined by jet pressure, nozzle geometry, and dose volume [231]. These technologies can enable the delivery of hydrophilic drugs, peptides, proteins, vaccines, and nucleic acids, but their performance and safety cannot be generalized across all devices. Their major limitations include device and formulation complexity, dose-loading constraints, cost, the need for operator or patient training, pain, erythema, and procedure-dependent variability.

Strategy selection should therefore begin with the drug, target tissue, required dose, and intended local or systemic exposure rather than with the nominal enhancement ratio. Small molecules with moderate lipophilicity may be delivered using passive formulations, CPEs, or supersaturation-based systems. When insufficient solubility, stability, or local retention limits performance, lipid-based or vesicular carriers may be appropriate, provided that adequate free-drug activity is maintained. Hydrophilic or ionized small molecules may benefit from iontophoresis, whereas peptides, proteins, nucleic acids, and other macromolecules typically require active approaches such as MNs, electroporation, jet injection, ultrasound, or appropriately designed combination systems. Follicular, nail, scar, and hyperkeratotic targets additionally require site-specific retention or controlled barrier modification. The principal selection criteria, representative applications, and limitations of these approaches are summarized in Table 10. Overall, no single enhancement strategy is universally superior. Strategy selection should be guided by the dominant delivery barrier and should balance drug properties, target compartment, required exposure, safety, manufacturability, patient acceptability, and regulatory feasibility. When solubility, SC transport, and target-site retention are simultaneously limiting, combination approaches may be justified, provided that each component has a defined and experimentally distinguishable function (Table 11). Table 12 extends this comparison by distinguishing consistently supported findings from unresolved or conflicting evidence and by relating each strategy to its predominant level of translational support. Together, these analyses provide the basis for the evidence hierarchy and clinical translation discussed in Section 8.

**Table 10:** Practical strategy-selection framework for skin-permeability enhancement based on drug properties, target tissue, and delivery requirements [6,9,10,104,230,231,232].

Delivery Challenge, Drug Property, or Target Tissue	Potentially Suitable Enhancement Strategy	Selection Rationale/Key Control Parameter	Major Limitation
Small, moderately lipophilic molecules, generally MW < 500 Da and LogP 1–4, with insufficient but potentially correctable passive flux and a low daily dose requirement	CPEs, supersaturation-based systems, or film-forming systems	Enhancer type and concentration, saturation ratio, solvent evaporation, and drug–vehicle–skin partitioning	Irritation, increased TEWL, crystallization, and variable enhancement
Poorly water-soluble small molecules	Nanoemulsions, lipid-based carriers, vesicular systems, or co-solvent-based supersaturated formulations	Drug solubility and free-drug activity, carrier composition, droplet or vesicle size, release rate, and crystallization control	Precipitation, physical instability, drug leakage, and reduced thermodynamic activity caused by excessive solubilization
Highly lipophilic drugs with excessive SC retention and insufficient onward diffusion	Vehicles optimized for drug–vehicle–skin partitioning; controlled-release, lipid-based, or vesicular systems	Drug–vehicle affinity, SC-to-viable-skin partitioning, vehicle polarity, and release rate	Persistent SC retention, limited deeper-tissue or systemic flux, and formulation-dependent performance
Hydrophilic or ionized small molecules with poor passive permeability	Iontophoresis; electroporation; CPE-assisted delivery as an adjunctive approach	Drug charge and ionization, formulation pH, current density and duration, pulse voltage, and competing ions	Device dependence, skin irritation, electrochemical instability, and limited deliverable dose
Drugs requiring prolonged epidermal or dermal retention	Hydrogels, film-forming systems, occlusive vehicles, nanoparticles, or vesicular carriers	Rheology, film formation, occlusivity, local residence time, drug-release rate, and skin deposition	Variable release, burst release, post-application crystallization, and inconsistent applied dose
Follicular or pilosebaceous targeting	Low-viscosity solutions or sprays, nanoemulsions, deformable vesicles, or polymeric nanoparticles	Particle or droplet size, formulation viscosity, affinity for sebum, follicular deposition, and local release	Variable follicular uptake, limited follicular loading, and dependence on anatomical site and follicular condition
Delivery through the nail plate or hyperkeratotic tissue	Hydration or keratolytic pretreatment, iontophoresis, fractional laser, or controlled thermal ablation	Barrier thickness, hydration state, keratolytic concentration, current density, and applied energy	Slow diffusion, prolonged treatment, local irritation, discomfort, and procedure-dependent variability
Peptides, proteins, vaccines, or other macromolecules requiring intradermal or systemic delivery	Dissolving, hollow, or hydrogel-forming microneedles; jet injection; selected ultrasound- or electroporation-assisted systems	Drug loading, biomolecule stability, needle geometry and insertion depth, jet pressure, and device operating conditions	Limited loading capacity, biomolecule instability, incomplete delivery, device complexity, and manufacturing challenges
Nucleic acids, siRNA, plasmid DNA, or other payloads requiring intracellular delivery	Microneedle-assisted nanoparticle delivery, electroporation, or other carrier–device combination systems	Cargo protection, particle size and charge, pulse conditions, cellular uptake, and endosomal escape	Enzymatic degradation, intracellular delivery barriers, formulation complexity, and limited clinical translation
Rapid intradermal or emergency administration	Hollow microneedles, jet injection, or active wearable delivery systems	Dose volume, flow rate, injection pressure, penetration depth, and dose reproducibility	Device cost, user training, discomfort, dose–volume limitations, and administration variability
Localized delivery into scars, plaques, or other treatment-resistant lesions	Microneedle pretreatment, fractional laser, thermal ablation, or localized iontophoresis	Treatment depth, energy or current setting, lesion thickness, local drug retention, and tissue tolerability	Pain, erythema, procedure-dependent variability, and risk of excessive local tissue injury
Multiple concurrent barriers, such as poor solubility combined with high molecular weight, insufficient retention, or resistant target tissue	Rational combination of chemical, vesicular, supersaturation-based, and physical approaches	Mechanistic complementarity, formulation–device compatibility, sequence of application, and cumulative safety	Increased formulation and device complexity, regulatory burden, cost, reproducibility concerns, and cumulative irritation

**Table 11:** Representative recent combination approaches in topical and transdermal delivery.

Combination Approach	Drug/Active Payload	Experimental Condition	Key Outcomes	Reference
Chemical enhancer + controlled polymeric patch	Methotrexate for psoriasis	Methotrexate was loaded into an ethyl cellulose:hydroxypropyl methylcellulose patch, and eucalyptus oil, N-methyl-2-pyrrolidone, Tween 80, and oleic acid were compared as penetration enhancers.	The optimized 10% oleic acid patch reduced stratum corneum lipid melting transition by 13.5 °C, increased permeability 9.8-fold versus control, and improved pharmacokinetics with C_max_ 183.29 ng/mL, t_1/2_ 22.19 h, AUC 3123.68 ng·h/mL, and mean residence time (MRT) 26.73 h.	[233]
Vesicular + chemical enhancer	Loxoprofen for transdermal nonsteroidal anti-inflammatory drug (NSAID) delivery	Optimized ethosomes contained 1% loxoprofen, 1% egg-yolk lecithin, 30% ethanol, and 5% propylene glycol; release and permeation were compared with hydroethanolic solution using excised rat skin.	Optimized ethosomes showed 164.2 ± 19 nm vesicle size, PDI 0.280 ± 0.028, +45.1 ± 4.5 mV zeta potential, and 96.8 ± 0.43% entrapment. Ex vivo flux increased from 0.0118 to 0.0698%/cm^2^/min, and permeability coefficient increased from 0.0007 to 0.0041 cm/min, corresponding to approximately 7-fold higher flux, 6-fold higher permeability, and Papp enhancement ratio 5.85.	[234]
Supersaturation-based film-forming system + volatile solvent/plasticizer	Caffeine, testosterone, and triiodothyronine models for transdermal/local delivery	A Eudragit^®^ E PO film-forming solution using isopropanol/ethanol and plasticizers was designed to increase thermodynamic activity after solvent evaporation; pig skin Franz diffusion and skin retention studies were performed.	The optimized film formed within about 5 min. For caffeine, the PEG-containing system at 100 µL produced flux of 3.65 ± 1.91 µg/cm^2^/h and Q24 50.12 ± 11.63%, higher than TBC- or triacetin-containing systems. For triiodothyronine, the authors associated lower applied volume with faster evaporation, supersaturation, and improved skin retention.	[96]
Nanocrystalline drug reservoir + physical microneedle array	Imiquimod for dermal immunomodulation	Nanocrystalline imiquimod was incorporated into fast-dissolving poly(vinyl alcohol) microneedle arrays and compared with semisolid nanocrystalline preparations using porcine ear skin	Microneedle arrays reduced the applied imiquimod dose by 93% compared with semisolid formulations while maintaining 24 h permeation even when skin contact time was <1 h. In Franz diffusion testing, IMINeedle delivered 9 ± 1.13% of dose versus 0.28 ± 0.07% for IMIGel and 0.4 ± 0.10% for IMISol+.	[235]
Ionic liquid + iontophoresis	Apremilast, aripiprazole, indomethacin	Five choline-based ionic liquids were synthesized and combined with iontophoresis to improve delivery of sparingly soluble model drugs.	Choline cinnamate, [Cho][Cin], markedly improved transdermal delivery of the tested poorly soluble drugs. The combined ionic liquid–iontophoresis system further enhanced weak base drug permeation, supporting its use for poorly soluble and ionizable molecules.	[174]
Paper battery-powered iontophoresis + microneedle patch	Triamcinolone acetonide	Hypertrophic scar tissue model; paper battery-powered iontophoretic microneedle patch was evaluated for active scar drug delivery.	The system delivered 90.19% of triamcinolone acetonide into hypertrophic scar tissue in vitro and reduced profibrotic markers, including transforming growth factor beta 1 (TGF-β1) and collagen I, indicating improved antifibrotic efficacy.	[236]
Hydrogel microneedle + iontophoresis + wearable self-aid device	Epinephrine	Wearable microneedle chip with conductive drug hydrogel, iontophoretic electrodes, and spring-driven insertion; tested in vitro and in a piglet hemorrhagic shock model.	In vitro delivery rate was 0.02642–0.1059 mg/h/cm^2^. In vivo, the device reversed life-threatening shock in a piglet model, showing potential for emergency self-administration.	[237]
Self-propelled hollow microneedle system + catalytic chemical pressure generation	Levonorgestrel	Battery-free bionic microneedle system using Pt nanoparticle/H_2_O_2_ catalytic oxygen generation; optimized at 15% H_2_O_2_, 2.5 µL, 3 min; evaluated in vitro and in rats.	The platform retained >90% liquid over 15 days and maintained levonorgestrel concentration changes below 10%. In rats, plasma levonorgestrel remained above the therapeutic threshold after on-demand dosing, with no obvious local skin inflammation.	[238]
Dual-pump piezoelectric micropump + hollow microneedles	Insulin	Wearable transdermal device integrating a dual-pump valveless piezoelectric micropump with a 7 × 7 hollow microneedle array; insulin dose 0.5 IU/kg tested in diabetic mice.	At 40 V and 600 Hz, the micropump achieved 1.61 mL/min flow. In diabetic mice, blood glucose decreased to near-normal levels within approximately 1 h, demonstrating precise active insulin delivery.	[239]
Dissolving bubble microneedle + multidrug compartmentalized delivery	Dipotassium glycyrrhizinate, salicylic acid, PIONIN/quaternium-73	Hyaluronic-acid dissolved bubble microneedle patch; 10 × 10 array, 500–550 µm height; drug-loaded compartments tested in porcine skin and *Cutibacterium acnes*-induced mouse ear acne model.	Microneedles inserted ~350 µm into porcine skin and nearly dissolved within 120 s. Salicylic acid release reached ~95% by 6 h, while dipotassium glycyrrhizinate and PIONIN showed slower release. In vivo, the system reduced ear swelling, lowered IL-6, increased IL-10, and nearly eliminated *C. acnes*.	[147]
Size-switchable responsive nanoparticles + microneedles	Methotrexate prodrug, DMTX	Hyaluronic-acid-decorated HPD6 nanoparticles delivered through microneedles for psoriasis; particles were designed to respond to mildly acidic and H_2_O_2_-rich psoriatic microenvironments.	Nanoparticles switched from ~196 nm to ~26 nm under pathological stimuli. Reported release reached 94.8% at pH 5.5 and 85.8% with 1 mM H_2_O_2_ at 48 h. Microneedle delivery prolonged skin retention and reduced epidermal thickening and inflammatory cell infiltration in psoriatic mice.	[240]
Calcium phosphate siRNA nanoparticles + polysaccharide microneedles	Slc31a1 siRNA + *Bletilla striata* polysaccharide	MC903-induced atopic dermatitis (AD)-like mouse model; microneedle-mediated transdermal gene delivery targeting cuproptosis–pyroptosis crosstalk.	The platform achieved efficient transdermal gene silencing and reduced skin inflammation, epidermal hyperplasia, pruritus, and T-helper 2/T-helper 17 (Th2/Th17) immune responses, supporting a dual drug–gene immunomodulatory strategy.	[241]
Core–shell microneedle + antimicrobial peptide + engineered exosomes + near-infrared (NIR) photothermal therapy	Pleurocidin (PDA) + CoQ10-engineered mesenchymal stem cell (MSC) exosomes	*Staphylococcus aureus*-infected diabetic mouse wound model; gelatin methacryloyl (GelMA)/hyaluronic acid methacrylate (HAMA) core–shell microneedles with PDA nanoparticles and NIR irradiation.	Pleurocidin showed rapid release, reaching 81.69 ± 3.01% within 8 h, whereas engineered exosomes were released gradually over 18 days. With NIR treatment, bacterial survival decreased to 1.71 ± 0.36% for S. aureus and 1.86 ± 0.17% for *E. coli*, while wound healing was accelerated through antimicrobial, antioxidant, anti-inflammatory, and pro-angiogenic effects.	[242]
Reactive oxygen species (ROS)-responsive core–shell microneedle + ultrasound-activated sonodynamic gas therapy	L-arginine-modified copper–cysteine nanoparticles + luteolin	Methicillin-resistant *Staphylococcus aureus* (MRSA)-infected diabetic rat wound model; ultrasound conditions included 1 W/cm^2^, 1 MHz, 8 min.	Ultrasound-triggered nanoparticles generated ROS and nitric oxide for antibacterial therapy, while luteolin was released under ROS-rich conditions to suppress inflammation and oxidative injury. MRSA survival decreased to 4.39 ± 1.87%, and encapsulated microneedles reduced MRSA and *E. coli* survival to approximately 4–5%.	[243]
Suction-assisted microneedle + membrane-cloaked mesoporous polydopamine nanoparticles	Metformin	Androgenetic alopecia model; short microneedles combined with suction-assisted insertion, H_2_O_2_-responsive mesoporous polydopamine nanoparticles, and fusion membranes derived from M2 macrophages and dermal papilla cells.	The system improved follicle-targeted delivery, reduced inflammatory and oxidative stress signals, and promoted hair-growth regulation with minimally painful delivery.	[244]
Microneedle codelivery + plant extracellular vesicles + drug nanoparticles	Minoxidil nanoparticles + *Platycladus orientalis*-derived extracellular vesicles	Androgenetic alopecia treatment model; microneedles used to codeliver minoxidil nanoparticles and plant-derived extracellular vesicles.	The combined system enhanced hair regrowth by improving minoxidil deposition and restoring the local follicular microenvironment. Reported safety observations indicated no evident skin irritation or systemic toxicity.	[245]

**Table 12:** Evidence consistency, remaining uncertainty, and translational proximity of major topical and transdermal delivery strategies.

Strategy	Consistently Supported Findings	Predominant Evidence Level	Remaining Uncertainty or Conflicting Findings	Translational Interpretation
Conventional vehicles and passive patches	Drug release, free-drug activity, vehicle–SC partitioning, adhesion, and wear conditions jointly determine delivery. Successful passive systemic delivery is consistently restricted mainly to small, potent, moderately lipophilic drugs with low daily dose requirements [5,6,246,247,248,249,250,251,252,253,254,255,256,257,258].	E4–E6 for selected marketed products; E1–E3 for mechanistic optimization	Molecular weight and LogP alone do not predict success. Product approval establishes efficacy and safety of the complete drug product but does not isolate the vehicle contribution or demonstrate increased intrinsic SC permeability.	Clinically established for carefully selected drug–product combinations but not broadly generalizable to hydrophilic, high-dose, or macromolecular payloads.
Supersaturated and evaporative systems	Solvent evaporation can increase drug saturation and thermodynamic activity, producing a transient increase in skin partitioning and flux while the drug remains molecularly dissolved. Crystallization reduces the dissolved fraction and eliminates much of this advantage [9,10,11,12,43,44,45,46,47,48,49,50,51,52,53,54,55,56,57,58].	E1–E2 predominant; selected product-level E4–E6 evidence	The magnitude and duration of useful supersaturation vary with evaporation rate, residual solvent composition, drug loading, skin conditions, and nucleation kinetics. Antinucleants and solubilizers may delay precipitation but can also reduce free-drug activity through excessive stabilization or solubilization.	Translation requires characterization of the transformed residual film, crystallization induction time, drug state, and delivery under clinically realistic application conditions rather than evaluation of the initial formulation alone.
Chemical permeation enhancers	CPEs can increase small-molecule delivery by modifying SC lipid order, lipid–protein interactions, drug solubility, or vehicle-to-skin partitioning. Their effects are consistently concentration-, drug-, vehicle-, and skin-model-dependent [56,59,60,61,62,63,64,65,66,67,68,69,70,71,72,73,74,75,76,77,78,79,80,81,82,83,84,85,86,87,88,89,90,91,92,93,94,95,96,97,98,99,100,101,102,103,104,105,106,107,108,109,110,111,112,113,114,115].	E1–E2 predominant; selected E3–E4 and product-level E6 evidence	Enhancer rankings and enhancement ratios vary markedly across drugs and models. Large fold increases may reflect a low control flux or excessive barrier disruption. Stronger enhancement does not necessarily provide better human delivery or acceptable repeated-use tolerability. Synergy between enhancers is often claimed without matched single-enhancer controls.	Most suitable for small molecules with potentially correctable passive flux. Development should be based on absolute delivered dose, barrier recovery, human skin reproducibility, irritation, and final-product stability rather than enhancement ratio alone.
Vesicular and lipid-based carriers	These systems reproducibly improve drug solubilization, surface residence, controlled release, and cutaneous or follicular deposition compared with simple solution or conventional vehicle controls. Lipid exchange, hydration, partial occlusion, and surface drug release can contribute to delivery [116,117,118,119,120,121,122,123,124,125,126,127,128,129,130,131,132,133,134,135,136,137,229,230].	E1–E3 predominant; E4–E6 evidence remains sparse	Evidence that intact vesicles routinely cross the intact SC and penetrate deeply remains inconsistent. Apparent penetration depends on carrier composition, deformability, labeling method, imaging resolution, comparator selection, and skin model. Increased deposition does not establish intact-carrier transport or clinical superiority.	Most credible as formulation platforms for solubilization, local retention, follicular targeting, or sustained release. Human skin validation, carrier fate, long-term stability, drug leakage, scale-up, and batch reproducibility remain major requirements.
Microneedle-based delivery	MNs reproducibly create transient microchannels and bypass the diffusional resistance of the SC. Increased delivery of poorly permeable small molecules, peptides, proteins, and carrier-associated payloads is consistently demonstrated. Selected controlled human studies support improved clinical outcomes in specific applications [139,140,141,142,143,144,145,146,147,148,149,150,151,152,153,154,259,260,261,262].	Broad E1–E3 evidence; selected E5 human evidence	Delivered dose varies with needle geometry, mechanical strength, insertion force, skin site, dissolution or infusion behavior, and patient application. Some clinical outcomes may include contributions from occlusion, tissue injury, radiofrequency, or the co-administered formulation. Long-term repeated-use evidence remains limited.	Among the more clinically advanced barrier bypass approaches, particularly for local treatment and selected macromolecules. Translation depends on dose reproducibility, scalable manufacturing, sterilization, usability, and drug–device combination-product regulation.
Iontophoresis	Low electrical current consistently enhances transport of charged and hydrophilic compounds through electrorepulsion and electroosmosis. Drug input can be modulated through current density and treatment duration, providing greater control than passive diffusion [155,156,157,158,159,160,161,162,163,164,165,166,167,168,169,170,171,172,173].	E1–E3 predominant; selected E4–E5 evidence	Flux is not determined by current alone and varies with molecular charge, competing ions, formulation conductivity, electrode design, skin resistance, and treatment duration. Delivery efficiency generally decreases with increasing molecular size. Irritation and electrochemical instability may limit exposure.	Most appropriate when charged or hydrophilic drugs require controlled and relatively rapid delivery. Clinical value must justify device dependence and demonstrate reproducible dosing, electrode safety, and patient acceptability.
Electroporation	Short high-voltage pulses consistently create transient aqueous pathways and increase transport of hydrophilic molecules, peptides, proteins, and nucleic acid-based payloads under controlled experimental conditions [178,179,180,181,182,183,184,185,186,187,188,189].	E1–E3 predominant; limited E4–E5 evidence	Pore number, distribution, lifetime, and recovery vary with voltage, pulse duration, pulse number, electrode geometry, and skin properties. Increased delivery may be accompanied by discomfort, erythema, or tissue injury, and results from different devices cannot be directly generalized.	Promising for payloads excluded by passive diffusion, but broader translation requires standardized devices, validated pulse parameters, barrier recovery data, and a demonstrable clinical advantage over MNs or injection.
Thermal, laser, and radiofrequency-assisted delivery	Controlled heating or fractional ablation can reproducibly reduce SC resistance or create microchannels. Human studies support increased delivery or improved clinical outcomes in selected local applications [190,191,192,193,194,195,196,197,198,199,200,201,202,203,204,205,261,263,264].	E1–E4 evidence with selected E5 clinical studies	Clinical improvement cannot always be attributed to enhanced drug transport because laser, heat, and radiofrequency may independently alter blood flow, inflammation, or tissue remodeling. Energy dose, penetration depth, device type, operator technique, and post-treatment formulation differ substantially among studies.	Relatively close to clinical implementation for procedure-based local treatment. Delivery-specific efficacy should be separated from the direct therapeutic effect of the device using laser-only, drug-only, vehicle, and combination controls.
Ultrasound-mediated delivery	Low-frequency ultrasound generally produces greater enhancement than high-frequency ultrasound through cavitation, SC lipid disruption, and formation of transient aqueous pathways. Increased delivery of hydrophilic molecules and selected macromolecules is repeatedly reported [206,207,208,209,210,211,212,213,214].	E1–E3 predominant; selected E5 functional or clinical evidence	Reported enhancement ratios vary widely with frequency, intensity, duty cycle, exposure time, coupling medium, hydration, and use of chemical enhancers. Heating, coupling variability, barrier recovery, and delivered-dose reproducibility are not consistently evaluated.	Translational use requires calibrated devices, standardized treatment and coupling conditions, and human pharmacokinetic or tissue delivery evidence demonstrating a clinically meaningful dose.
Magnetophoresis	Static, oscillating, or rotating magnetic fields have increased permeation or altered skin retention of selected drugs in experimental studies without intentional SC ablation [174,175,176,177].	E1–E3; robust E4–E6 evidence is lacking	The driving force is relatively weak, and results vary with field strength, gradient, orientation, frequency, drug properties, and formulation. Some field conditions increase permeation, whereas others primarily increase skin retention, and the relative contributions of convection, follicular transport, and altered partitioning remain incompletely resolved.	Remains largely experimental. Advancement requires mechanistic validation, standardized field parameters, reproducible human skin data, and evidence that the benefit justifies an external device.
Jet injection	High-velocity liquid or powder jets reproducibly bypass the SC and deposit payloads directly into intradermal or deeper tissue compartments. Human studies support the feasibility of needle-free administration for selected drugs and vaccines [215,216,217,218,219,220,221,222,223,224,225,226].	E2–E5, depending on device and intended tissue depth	Penetration depth and dispersion vary with pressure, nozzle geometry, nozzle-to-skin distance, application angle, dose volume, viscosity, and tissue mechanics. Pain, bruising, splash-back, incomplete dosing, and cross-contamination remain device-specific concerns. Much of the clinical evidence concerns intradermal or intramuscular injection rather than transdermal permeation.	Clinically more mature as a needle-free injection technology than as a conventional topical or transdermal platform. Development requires accurate control of deposition depth, dose recovery, cartridge design, and administration reproducibility.
Combination and responsive systems	Combining complementary mechanisms can increase delivery beyond that achieved by an individual formulation or device, particularly when one component improves solubilization and another bypasses or perturbs the SC [231,232,233,234,235,236,237,238,239,240,241,242,243,244,245,259,260,261,262,263,264].	E1–E3 predominant; selected E5 evidence	True synergy is frequently uncertain because many studies lack factorial designs, matched single-component controls, or direct measurement of tissue exposure. Device-only effects, occlusion, tissue remodeling, and formulation effects may confound clinical outcomes. Greater complexity may also reduce stability, manufacturability, and regulatory clarity.	Combination systems should advance only when each component has a defined function and its incremental contribution is demonstrated using appropriate controls. Additional complexity must produce a clinically meaningful benefit rather than a larger experimental enhancement ratio alone.

Note: Evidence-level definitions: E1, in vitro release, permeation, reconstructed skin, or synthetic membrane studies; E2, ex vivo animal or human skin studies; E3, in vivo animal pharmacokinetic, tissue distribution, efficacy, or safety studies; E4, human pharmacokinetic, dermatokinetic, tissue deposition, or mechanistic studies; E5, controlled clinical studies in humans; E6, regulatory approval or established commercial use.

## 8. Clinical Translation and Recent Case Studies

### 8.1. Evidence Hierarchy and Marketed Products

Clinical translation in topical and transdermal drug delivery should be interpreted according to the level of supporting evidence. In ascending order of translational proximity, the evidence considered in this review is from in vitro release and permeation studies using synthetic membranes or reconstructed skin, ex vivo animal or human skin studies, in vivo animal pharmacokinetic or efficacy studies, human pharmacokinetic or dermatokinetic studies, controlled clinical trials, and regulatory approval or established product use. Each level supports a different conclusion. In vitro and ex vivo studies can identify release, partitioning, barrier interaction, and permeation mechanisms but cannot independently establish human therapeutic efficacy. Animal studies can provide systemic exposure, tissue distribution, efficacy, and preliminary safety data, but species-dependent differences in skin structure limit direct clinical extrapolation. Human pharmacokinetic, tissue deposition, or mechanistic studies provide more direct evidence of delivery, whereas controlled clinical trials establish product-level efficacy and safety. Regulatory approval represents successful product-level translation but does not necessarily identify the contribution of the vehicle or demonstrate that intrinsic SC permeability has been increased.

Clinical translation is a more rigorous indicator of transdermal feasibility than the in vitro enhancement ratio alone. Although chemical enhancers, vesicular systems, gels, patches, and lipid-based vehicles can improve drug solubilization, SC partitioning, and diffusion, successful passive systemic transdermal delivery remains largely restricted to molecules with favorable physicochemical and pharmacological profiles. Most clinically successful passive transdermal drugs are relatively small and sufficiently lipophilic to partition into the SC but not so lipophilic that they are irreversibly retained in skin lipids. They are also pharmacologically potent enough to require only low daily doses. This principle is consistent with the well-known “500 Dalton rule”, which emphasizes that passive transdermal systems mainly favor low-molecular-weight, moderately lipophilic, and low-dose drugs [5,246]. The established passive products and recent formulation- and device-enabled clinical cases summarized in Table 13 illustrate the distinct requirements for local cutaneous and systemic transdermal translation. Nicotine (162.23 Da, LogP ~1.2), fentanyl (336.5 Da, LogP ~4.0), estradiol (272.4 Da, LogP ~4.0), clonidine (230.09 Da, LogP ~1.6), rivastigmine (250.34 Da, LogP ~2.3), testosterone (288.4 Da, LogP ~3.3), nitroglycerin (227.09 Da, LogP ~1.6), and lidocaine (234.34 Da, LogP ~2.3) all fall below the 500 Da threshold. These products occupy a relatively narrow physicochemical space: they are small molecules with low-to-moderate or formulation-manageable lipophilicity, enabling partitioning into the SC while still allowing release into the viable skin or systemic circulation.

However, molecular weight and LogP alone do not guarantee successful translation. Fentanyl and estradiol are clinically feasible largely because they are highly potent and require low daily doses, whereas nicotine, clonidine, rivastigmine, and nitroglycerin rely on controlled patch release to maintain therapeutic exposure over extended periods. Several marketed products also have important limitations, including heat-related dose dumping and interindividual variability for fentanyl, delayed onset and rebound hypertension for clonidine, adhesion problems and local irritation for rivastigmine, nitrate tolerance for nitroglycerin, large-area application and secondary-transfer risk for testosterone gel, and primarily local rather than systemic delivery for lidocaine patches. Therefore, marketed transdermal products should be regarded as carefully selected examples of suitable drug–formulation matching rather than evidence that passive skin delivery is broadly applicable. Accordingly, drugs with molecular weights above 500 Da, very low or very high LogP, high dose requirements, strong hydrophilicity, or macromolecular structures—such as peptides, proteins, nucleic acids, vaccines, and many hydrophilic drugs—generally require advanced formulation or device-assisted approaches. These may include supersaturating systems, optimized chemical enhancers, lipid or vesicular carriers, microneedles, iontophoresis, electroporation, ultrasound, thermal ablation, or hybrid systems designed to overcome the intrinsic limitations of passive diffusion across the SC. These benchmarks provide the clinical context for interpreting the recently approved topical products and device-assisted approaches discussed below.

### 8.2. Recent Clinical Translation of Topical Products

Recent approvals and indication expansions of topical immunomodulators demonstrate that potent small molecules can achieve clinically effective local pharmacology when combined with suitable vehicles. Ruxolitinib cream 1.5% has been approved for AD and nonsegmental vitiligo, whereas tapinarof cream 1% has been approved for plaque psoriasis and AD [247,248]. These products achieve localized Janus kinase 1/2 (JAK1/2) or aryl hydrocarbon receptor (AhR) modulation without deliberate physical disruption of the SC. The roflumilast product family further illustrates how adaptation of the dosage form can extend the clinical utility of a single active compound. Roflumilast creams at different strengths have been approved for plaque psoriasis and AD, whereas roflumilast foam 0.3% has been approved for seborrheic dermatitis and scalp and body psoriasis [249,250]. Human pharmacokinetic studies showed approximately 1.5% topical bioavailability for roflumilast cream and substantially higher cutaneous than plasma concentrations, providing direct support for localized skin exposure [251]. In the 2025 ARRECTOR trial, roflumilast foam achieved week-8 Scalp Investigator Global Assessment success in 66.4% of patients compared with 27.8% receiving vehicle foam [252]. However, because the foam was not directly compared with a cream or ointment, the trial establishes the efficacy of the foam product but not the superiority of foam as a dosage form. Additional approvals extend topical treatment to difficult-to-treat sites and pediatric populations. Delgocitinib cream received EU marketing authorization in 2024 for moderate-to-severe chronic hand eczema in adults for whom topical corticosteroids are inadequate or inappropriate. In the DELTA 1 and DELTA 2 trials, treatment success was achieved in 19.7% and 29.1% of delgocitinib-treated patients, respectively, compared with 9.9% and 6.9% of vehicle-treated patients [253,254]. Difamilast ointment 1% was subsequently approved by the FDA in 2026 for mild-to-moderate AD in patients 2 years of age and older. Across three vehicle-controlled studies, week-4 Investigator Global Assessment success ranged from 21% to 47% with difamilast and from 3% to 18% with the vehicle [255]. These controlled clinical data support the efficacy of locally acting JAK and phosphodiesterase-4 (PDE-4) inhibitors as complete topical drug products. Nevertheless, they do not establish that the cream or ointment independently increases skin permeability because cutaneous drug concentrations, tissue distribution, and direct permeation were not compared with alternative vehicles.

Site-adapted products represent a related but distinct translational pathway. Clascoterone cream provides local androgen receptor antagonism for acne, although follicular drug deposition has not been directly quantified [256]. Sofpironium topical gel, approved in 2024 for primary axillary hyperhidrosis, enables the anatomically restricted anticholinergic treatment of eccrine sweating [257]. Berdazimer topical gel, also approved in 2024, generates and releases nitric oxide locally for molluscum contagiosum [258]. Its principal formulation innovation is controlled generation of the active agent at individual lesions rather than the enhancement of passive SC permeability. Collectively, these cases show that successful cutaneous translation may result from anatomical targeting, controlled local generation or release, reduced systemic exposure, and patient-compatible application rather than increased intrinsic SC permeability.

### 8.3. Human Evidence for Physical Enhancement and Remaining Gaps

Physical barrier bypass systems can provide a more direct mechanistic link between enhanced transport and clinical outcome when appropriate controls are included. In a 2025 randomized split-body study, 22 patients contributed 132 psoriatic lesions, of whom 18 patients with 108 lesions completed the 2-week treatment. Improvement in modified Psoriasis Area and Severity Index scores was 80.4% with the dissolving MN patch compared with 64.6% using a no-needle patch and 55.5% using ointment alone. Supporting in vitro studies showed an approximately 2.1-fold increase in delivery relative to ointment alone, while ex vivo analysis confirmed microchannel formation [259]. Because the drug was applied before patch placement and a no-needle patch was included as a control, the study linked microneedle-generated microchannels, increased experimental delivery, and improved clinical response more directly than studies evaluating clinical efficacy alone. However, the delivery measurement itself remained based on in vitro and ex vivo evidence rather than human tissue pharmacokinetics. Recent studies have also combined MN-mediated barrier disruption with topical treatment or external activation. In a randomized split-face trial, microneedling followed by a vitamin C/E/ferulic acid serum produced marked or near-total improvement in 89.3% of treated sides compared with 7.1% of sides receiving microneedling with the placebo serum [260]. In a controlled split-neck trial, fractional microneedle radiofrequency combined with the same serum reduced wrinkle severity by 29.9%, compared with 18.0% using radiofrequency microneedling alone [261]. These contralateral designs support an additional clinical contribution from the topical formulation, although intradermal antioxidant concentrations were not measured, and the procedures themselves can induce tissue remodeling. A 2026 wearable near-infrared-responsive MN patch produced approximately 14.1% improvement in crow’s feet wrinkles with near-infrared actuation, compared with 1.9% using the non-actuated patch [262]. This pilot study provides preliminary human evidence for externally controlled microneedle dissolution, but its small population and short duration limit broader conclusions regarding long-term efficacy, safety, and reproducibility.

Laser-assisted topical delivery has similarly been evaluated in dense scar tissue. Fractional CO_2_ laser produces microthermal channels that can facilitate the immediate topical application of triamcinolone acetonide, 5-fluorouracil, or vitamin C. Clinical studies published in 2025 reported improvements in scar height, pliability, pigmentation, vascularity, and modified Manchester scores [263,264]. However, fractional laser treatment can independently induce collagen remodeling, and one study did not include separate laser-only and topical-only control groups. These findings support the clinical feasibility of combined laser and topical treatment but do not fully distinguish enhanced drug transport from the direct therapeutic effects of the laser procedure.

Accordingly, the conclusions supported by human clinical evidence are that appropriately formulated potent small molecules can provide effective local cutaneous therapy, site-adapted dosage forms can improve practical treatment and limit systemic exposure, and selected MN- or laser-assisted approaches can improve clinical outcomes in specific applications. Human pharmacokinetic evidence additionally supports localized exposure for certain products such as roflumilast cream and topical finasteride spray. However, evidence for direct permeability enhancement in humans remains more limited and requires pharmacokinetic, dermatokinetic, tissue deposition, or mechanistic measurements with appropriate vehicle or device controls. In contrast, generalized conclusions concerning chemical enhancer mechanisms, comparative superiority of vesicular or lipid-based carriers, intact vesicle penetration, supersaturation-driven delivery, extracellular vesicle transport, nucleic acid delivery, and most responsive or multi-component platforms continue to rely predominantly on in vitro, ex vivo, or animal studies. These findings support mechanistic plausibility and further development but should not be interpreted as proof of clinical superiority. Representative passive products, recent topical approvals, and device-assisted clinical studies are compared in Table 13 according to their regulatory or clinical status, quantitative evidence, delivery-specific significance, and remaining limitations.

## 9. Future Perspectives

Future development of topical and transdermal drug delivery should be considered based on translational readiness rather than technological novelty alone. Conventional passive formulations will remain most appropriate for potent, stable, and moderately lipophilic small molecules, whereas peptides, proteins, nucleic acids, vaccines, and other macromolecular therapeutics will generally require physical barrier bypass or combination delivery systems. Emerging technologies can therefore be broadly positioned as near-term platforms with human evidence, intermediate-stage systems supported mainly by preclinical or prototype studies, and largely experimental disease-responsive or digitally controlled systems. Among the emerging approaches, MN-assisted delivery and fractional laser-assisted topical delivery appear closest to broader clinical implementation. Dissolving MN patches, conventional microneedling, and fractional MN or laser procedures have already been evaluated in controlled human studies for psoriasis, photoaging, and scar treatment [259,260,261,262,263,264]. These platforms are based on a directly verifiable mechanism—creation of transient microchannels across the SC—and can be evaluated using established endpoints such as insertion reproducibility, drug delivery, skin recovery, irritation, pharmacokinetics, and clinical response. Nevertheless, their broader implementation still requires standardized application procedures, reliable dose loading and delivery, scalable manufacturing, long-term stability, repeated use safety, and larger clinical trials. Fractional laser-assisted delivery additionally requires separation of the therapeutic effect of the laser itself from the contribution of enhanced topical drug transport.

Wearable and electronically controlled delivery devices occupy an intermediate translational position. Digitally triggered microneedle patches, iontophoretic hollow MN systems, and osmotic MN devices have demonstrated programmable, on-demand, or sustained delivery in laboratory and animal models [274,275,276,277,278]. For example, electrically triggered MN systems can provide spatially and temporally controlled release, whereas combined iontophoresis–microneedle systems can increase the delivery of charged molecules and macromolecules [274,275]. A wearable osmotic MN patch has also demonstrated prolonged drug delivery in rodents and canines, together with preliminary human tolerability [276]. However, most of these systems have not yet demonstrated reproducible therapeutic dosing and efficacy in adequately powered human trials. Their translation will depend on the simplification of device architecture, consistent insertion and actuation, power reliability, accurate dose control, acceptable wearability, and clinically meaningful advantages over conventional patches, injections, or existing drug delivery devices. Artificial intelligence-assisted formulation development may be implemented earlier than patient-facing smart delivery systems because it can be incorporated into existing pharmaceutical development workflows. Machine learning and Bayesian optimization approaches can integrate drug physicochemical properties, excipient composition, skin permeation data, rheology, stability, and safety constraints to guide formulation selection [59,279,280,281,282]. In one topical ibuprofen study, Bayesian optimization increased measured permeation flux from 11.28 ± 0.35 to 14.15 ± 0.77 μg/cm^2^/h after four optimization iterations [281]. This demonstrates potential for reducing empirical screening, but it remains a result of formulation development rather than evidence of clinical benefit. The usefulness of such models will depend on standardized input data, external validation, transparent model performance, and prospective confirmation. Variability in skin source, membrane model, dose, receptor medium, experimental duration, and reported endpoints currently limits the transferability of many skin permeation datasets [280,282].

In contrast, bioresponsive and disease-triggered delivery systems remain largely experimental. Platforms responsive to glucose, ROS, acidic pH, hypoxia, matrix metalloproteinases, cathepsins, bacterial hyaluronidase, or inflammatory enzymes offer the possibility of preferential drug release within diseased skin [274,283,284,285,286,287,288,289]. Preclinical studies have demonstrated enzyme-responsive or ROS-responsive drug release in models of psoriasis, infected wounds, and diabetic wounds. However, clinically relevant biomarker thresholds, spatial variability within lesions, extracellular accessibility of the trigger, response specificity, degradation products, and repeated-use safety remain insufficiently established. Multi-input or “logic-gated” systems introduce additional uncertainty because variation in one trigger may produce incomplete release, whereas excessive responsiveness may result in burst release or local toxicity. These platforms should therefore be considered proof-of-concept technologies until their activation thresholds, dose–release relationships, and therapeutic advantages are validated in human skin and clinical studies. Precision dermatology and personalized transdermal therapy also remain developmental rather than clinically established delivery strategies. Patient- and lesion-specific differences in age, anatomical site, SC thickness, hydration, sebum production, microbiome composition, inflammatory endotype, protease activity, oxidative stress, and prior treatment can influence drug permeation and tolerability [290,291,292]. These variables could eventually guide the selection of vehicle composition, enhancer intensity, local retention strategy, or physical barrier bypass methods. Diagnostic patches, superficial skin sampling, transcriptomic profiling, and biomarker-based treatment selection may support this approach. However, digital twin models and molecularly personalized formulation selection currently lack standardized datasets, prospective validation, and evidence that they improve treatment outcomes compared with conventional clinical assessment.

The principal regulatory challenges differ among these technology groups. MN, iontophoretic, laser-assisted, and electronically controlled systems may be regulated as drug–device combination products, requiring definition of the primary mode of action and coordinated evaluation of both formulation and device performance. Critical requirements include dose uniformity, needle or device integrity, insertion reproducibility, release kinetics, sterility or microbial control, stability, electrical and mechanical safety, and failure mode testing. Connected or software-controlled systems additionally require software validation, cybersecurity, data integrity, power supply reliability, and human factor assessment. Nanocarrier and biologically derived systems require the detailed control of particle or vesicle identity, composition, drug loading, release, aggregation, impurities, potency, immunogenicity, storage stability, and batch-to-batch consistency. For responsive platforms, regulators will also require reproducible activation thresholds, predictable off-state leakage, safe degradation products, and protection against unintended activation. In all cases, clinical development must demonstrate not only enhanced delivery, but also a meaningful therapeutic or practical advantage over an appropriate conventional formulation or device comparator. Accordingly, the most productive near-term direction is likely to be the refinement of technologies that already have a clear mechanism, reproducible manufacturing pathways, and preliminary human evidence, particularly simplified MN-assisted and device-assisted delivery systems. AI-guided formulation optimization may support this process if it is based on standardized and externally validated data. Bioresponsive, extracellular-vesicle-based, nucleic acid, multi-component, and closed loop digital systems offer important longer-term possibilities but require substantial mechanistic, manufacturing, safety, and clinical validation. Future progress should therefore be judged by advancement through the translational evidence hierarchy—from controlled in vitro and ex vivo studies to human pharmacokinetics, clinical efficacy, repeated-use safety, manufacturing validation, and regulatory feasibility—rather than by technological complexity alone.

## 10. Conclusions

The central conclusion of this review is that successful topical and transdermal drug delivery cannot be achieved simply by selecting the most potent permeation enhancer or the most advanced carrier. Delivery performance results from the coordinated interaction between drug physicochemical properties, therapeutic target, skin barrier condition, vehicle composition, excipient function, and formulation changes after application. In particular, evaporation, hydration, drug partitioning, supersaturation, and crystallization can substantially alter the effective driving force for permeation. Formulations intended for local skin targeting and those intended for systemic delivery must therefore be designed and evaluated using different performance criteria. No single enhancement strategy is universally superior. CPEs are comparatively simple and scalable but may exhibit drug-dependent efficacy, irritation, and interindividual variability. Vesicular and lipid-based systems can improve drug solubilization, skin deposition, and controlled release, although much of the supporting evidence remains limited to in vitro, ex vivo, or animal studies, and physical instability and manufacturing reproducibility remain important concerns. Supersaturated and vehicle-transforming formulations can increase thermodynamic activity but require the effective control of drug precipitation during storage and after application. Physical enhancement technologies can expand delivery to peptides, proteins, and other macromolecules, but their advantages must be balanced against dose-loading limitations, device reliability, patient usability, production cost, and drug-device regulatory requirements. Consequently, formulation performance should not be judged solely by fold increases in permeation. Absolute delivered dose, target tissue exposure, systemic absorption when intended, barrier recovery, repeated use safety, and evidence from human skin or clinical studies are more meaningful indicators of translational potential.

Future development should therefore prioritize the reduction in the gap between laboratory permeation enhancement and clinically reproducible drug delivery. This requires standardized and physiologically relevant test conditions, quantitative assessment of post-application vehicle transformation, human-skin validation, repeated-use tolerability studies, long-term stability testing, identification of critical quality attributes, and scalable manufacturing processes. Regulatory strategy and patient use considerations should also be incorporated at an early stage rather than addressed after formulation optimization. Emerging materials, responsive systems, wearable devices, and computational formulation tools may support this process, but their value will depend on whether they solve clearly defined clinical and manufacturing problems. Ultimately, the most successful topical or transdermal system will not be the formulation that produces the highest experimental permeability, but the one that delivers a reproducible therapeutic dose to the intended site while maintaining reversible barrier modulation, acceptable safety, manufacturability, stability, and practical usability.

## Figures and Tables

**Table 1:** Characteristics of major cutaneous permeation pathways.

Pathway	Typical Drug/Carrier Type	Approximate Molecular Weight or Particle Size Range	Reference
Intercellular (lipid)	Lipophilic small molecules with appreciable stratum corneum lipid partitioning	Best-supported passive range: <500 Da	[8,34]
Transcellular	Very small hydrophilic or amphiphilic molecules; mainly discussed for model permeants	No universally accepted exact molecular weight (MW) cut-off; best described as limited to very small molecules	[26,28,35]
Transfollicular (hair follicle)	Nanoparticles, vesicles, nanostructured lipid carriers, microspheres, and other particulate carriers for follicular reservoir targeting	Studied particle diameters: 122–1000 nm; deepest penetration in porcine follicles at 643–646 nm; optimal microsphere size in terminal human follicles ~1.5 µm	[36,37]
Sweat gland route	Ions and very small hydrophilic solutes; appendageal shunt route	No validated universal passive molecular weight cut-off; best supported for ions/very small hydrophilic solutes	[38,39,40]
Sebaceous/pilosebaceous	Small lipophilic molecules for follicle/sebum targeting; follicle-targeted nanoparticulate carriers	For freely diffusing small drugs: <500 Da. For carrier-based targeting, the relevant metric is particle diameter, with demonstrated sizes of 228 nm, 365 nm, and ~300 nm	[8,41,42]

**Table 2:** Comparative summary of major chemical permeation enhancers.

Mechanistic Class	Representative Enhancers	Primary Mechanism	Representative Enhancement Example	Translational Limitation	Reference
Solvent and thermodynamic activity modifiers	Ethanol, PG, DEGEE	Increased drug solubility, supersaturation, partitioning	Bimatoprost flux ↑4.6-fold; Itraconazole deposition ↑2024%	Evaporation-induced precipitation	[64,115]
Lipid fluidizers and disruptors	Oleic acid, IPM, Terpenes	Lipid disordering and fluidization	Olanzapine flux ↑3.3-fold	Irritation, concentration dependence	[80,81]
Protein/lipid interaction enhancers	DMSO, NMP	Keratin and lipid modification	Ibuprofen flux ↑16-fold	Irritation and toxicity concerns	[70,97,98]
Surfactant-based enhancers	SLS, Tween 80	Wetting, lipid extraction, micellization	Diazepam flux ↑9.8-fold	Barrier disruption	[94]
Multifunctional enhancers	DEGEE, TPGS	Solubilization + partitioning + retention	Formulation-dependent skin-permeation enhancement in Transcutol^®^- or TPGS/HPMC-containing systems	Formulation complexity	[51,52]

**Table 5:** Representative applications of iontophoresis in skin delivery, experimental conditions, and major outcomes [157].

Drug	Animal/Membrane Model Used	Experimental Conditions	Results	Reference
Buprenorphine	Human epidermal membrane	In vitro: Franz (vertical) diffusion cell; current: 0.5 mA/cm^2^; time: 4 h	8-fold higher delivery by anode compared to cathode	[165]
Diclofenac	Rabbit skin	In vitro: Current: 0.2 and 0.5 mA/cm^2^; time: 6 h; effect of current studied	Plasma concentration achieved in 1 h; delivery proportional to current (371 ± 141 µg/L at 0.5 mA/cm^2^ vs. 132 ± 62 µg/L at 0.2 mA/cm^2^)	[166]
Leuprolide (LHRH agonist)	Human epidermal skin	In vitro: Buffers pH 4.5 and 7.2; current: 0.5–2.3 mA/cm^2^	Permeation doubled at pH 7.2 compared to pH 4.5	[167]
Nalbuphine (Nb) and prodrugs	Intact skin, stratum corneum stripped skin, delipidized skin, Wistar rat skin	In vitro: Effect of prodrug lipophilicity on passive and iontophoretic permeation studied	Highest enhancement for Nb; decreased with increasing lipophilicity	[168]
Piroxicam	Human (ventral forearm)	In vivo: Two glass chambers; current: 0.3 mA/cm^2^ via phoresor II; tape-stripped SC	10-fold increase in permeation	[169]
Rotigotine	Human stratum corneum	In vitro: Side-by-side diffusion cell; studied drug concentration and co-ions (TEA, TBA); current: 0.05 mA/cm^2^	Flux increased with concentration; TEA increased flux, TBA showed no effect	[170]
Salbutamol	Artificial membrane (non-rate-limiting)	In vitro: Drug release from liquid crystalline vehicle studied	Enhanced flux observed	[171]
Thiocolchicoside	Rabbit and human skin	In vitro: Glass Franz diffusion cell	Enhanced flux compared to passive delivery	[172]
Timolol maleate (TM)	Rat, rabbit, guinea pig, mouse, and human skin	In vitro: Valia–Chien side-by-side diffusion cell; species comparison	Highest transport in human skin; lowest in rabbit	[173]
Apremilast, aripiprazole, indomethacin	Excised rat skin	In vitro: Ionic liquid–iontophoresis system; drug 4 mg in 4 mL ionic liquid/control vehicle; receptor phosphate-buffered saline (PBS) pH 6.8; 32 ± 0.5 °C; choline–cinnamate ionic liquid, [Cho][Cin], 20%; current 0.1–0.3 mA; negative-current direction favored	Negative current increased permeation/skin retention compared with positive current: apremilast ~3.2-/3.4-fold, aripiprazole ~5.0-/3.0-fold, indomethacin ~3.0-/1.9-fold. IL–iontophoresis increased apremilast permeation up to 16-fold, aripiprazole up to 14-fold, while indomethacin showed weaker synergy	[174]
Lidocaine	Rat skin	Smartphone-controlled iontophoresis-driven fiber-based microneedle patch; current adjustable 0–2 mA; in vitro groups included 0.5, 1, 1.5, and 2 mA for 6 h; selected in vivo condition 1 mA because electrode contact area was ~2 cm^2^ and acceptable current was ≤0.5 mA/cm^2^	LIDO/MN/1 mA iontophoresis for 10 min increased permeation to 895.7 ± 138.9 μg/cm^2^, versus 502.4 ± 37.05 μg/cm^2^ for MN alone and 17.51 ± 0.91 μg/cm^2^ passive control. Complete nociceptive blockade occurred within 5 min and analgesia persisted > 6 h; sustained mode-maintained analgesia for 20 h	[161]
Niacinamide	Ex vivo porcine ear skin	Self-powered Mg–MoO_3_ galvanic iontophoretic–electrochromic patch; 3.3 wt% niacinamide; electrochromic gauge used as charge-correlated dose indicator; daily treatment for 7 days; passive controls used 3.3 wt% and 20 wt% gels for 30 min	Ex vivo delivered dose correlated linearly with electrochromic propagation distance (r = 0.966); dose increased from 209 ± 7 μg at ~3 mm to 408 ± 68 μg at ~10 mm. In psoriatic mice, epidermal thickness approached healthy tissue values (~21–25 μm) and inflammatory morphology improved without systemic toxicity	[175]

**Table 6:** Topical delivery of drugs and macromolecules into skin using electroporation.

Drug/Molecules	Model	Outcomes	Reference
Vitamin C	In vitro human skin	Application of exponential pulses (60–100 V, 2.7–30 ms) facilitated drug transport; cream formulations and suspensions showed greater enhancement (193% greater for cream and 17.03% higher for suspension) of penetration across skin layers than in a no-pulse model.	[184]
Lidocaine	In vivo human skin	All concentrations of lidocaine produced significant changes from baseline in two or three efficacy measures.	[185]
Cyclosporine A	In vitro hairless rat skin	Use of single-pulse mode at a field strength of 200 V/cm and a 10 ms pulse interval resulted in the delivery of 87 ng of cyclosporine A per 0.87 cm^2^ of rat skin. This was a significant increase, by a factor of 8.5, in the delivery of cyclosporine A when compared to passive diffusion.	[186]
Phosphorothioate oligodeoxynucleotide (PS)	In vitro hairless rat skin	A few minutes after pulsing, fluorescein isothiocyanate-labeled PS was detected in the nuclei of keratinocytes at greater rates than in free form.	[187]
Myristylated peptide	In vivo mouse skin	The electroporation route elicited higher responses to myristylated peptide than normal intradermal needle immunization.	[188]
Reporter gene (pEGFP-N1) coding for Green Fluorescent Protein (GFP)	In vivo mouse skin	The plasmid penetrated the epidermis within minutes after electroporation and entered the keratinocyte cytoplasm within hours.	[189]
siRNA, including Cy3/Cy5-labeled siRNA and therapeutic FOXM1 siRNA	Mouse skin, porcine skin, human breast model simulation, and triple-negative breast cancer mouse model	A flexible noninvasive electroporation system enabled nucleic acid delivery into skin/subcutaneous tissue, with siRNA reaching approximately 500 µm in mouse skin and up to approximately 1 mm in porcine skin/human breast model simulations. FOXM1 siRNA delivery reduced tumor growth by approximately two thirds, decreased programmed death-ligand 1 (PD-L1) expression by approximately three quarters, and suppressed detectable lung metastasis in the tumor model.	[190]
Hyaluronic acid, ibuprofen, and diclofenac sodium	Animal and human skin	A wearable electroporation patch with serpentine interdigitated electrodes enhanced skin penetration of model hydrophilic and small-molecule drugs by approximately 2–3-fold compared with control treatment. The patch design localized the electric field near the skin surface and reduced deep-tissue voltage by >50%, with cytotoxicity and histology supporting biocompatibility.	[191]

**Table 7:** Representative effects of thermal ablation and heat-assisted approaches in topical and transdermal drug delivery.

Drug	Model	Outcomes	Reference
Testosterone	Applied to skin of healthy adult volunteers	Heat plus patch resulted in a mean maximum serum testosterone concentration of 939 ng/dL versus 635 ng/dL (patch only).	[199]
Methyl salicylate	Applied to skin of healthy adult volunteers	Absorption of methyl salicylate was increased more than threefold in subjects exercising in heat relative to controls.	[200]
Lidocaine	In vitro experiments on silicone membrane and human epidermis	The apparent lidocaine diffusion coefficient through a silicone membrane increased from 6.52 to 8.43 × 10^−4^ over the 32–45 °C temperature range; it increased from 7.74 × 10^−5^ cm^2^/h to 4.8 × 10^−4^ cm^2^/h in the human epidermis.	[201]
Methyl paraben (MP), butyl paraben (BP), and caffeine (CF)	In vitro experiments on artificial membranes and human epidermis	With an increase in temperature from 23 °C to 45 °C, the permeability concentration was increased by 1.74-, 1.48-, and 1.79-fold for MP, BP, and CF, respectively.	[202]
Nitroglycerin patch	Applied to skin of healthy adult volunteers	After 10 min of heating, the median (Walsh) plasma nitroglycerin level increased from 3.1 to 7.6 nmol/L.	[203]
Saturated solutions of parathion	In vitro experiments in porcine skin	An increase in temperature from 37 °C to 41 °C resulted in the enhanced permeation of parathion.	[204]
Triamcinolone acetonide	Applied to healthy female volunteers	Fractional Er:YAG pretreatment increased the permeability of triamcinolone acetonide from the solution/suspension by approximately 5-fold and enhanced its distribution within the stratum corneum.	[205]
Hyaluronic acid	Healthy male and female volunteers	Laser pretreatment increased penetration to 82.1 ± 7.1 µm with CO_2_ laser and 81.6 ± 11.5 µm with thulium laser, which was 3.5-fold higher without laser treatments	[206]
Insulin, methotrexate, rhodamine B, and dextrans	Mouse skin and in vivo diabetic and psoriasis mouse models	Mild photothermal treatment increased penetration of polydopamine nanoparticles measuring 2.4–406.9 nm by 5.5–7.1-fold. Dermal fluorescence increased 28.9-fold for rhodamine B, 24.6-fold for 5 kDa dextran, and 10.7-fold for 20 kDa dextran relative to nonheated controls. Heat-assisted transdermal insulin reduced blood glucose by 75%, while polydopamine nanoparticle–methotrexate co-delivery significantly improved psoriasis lesions.	[207]

**Table 8:** Summary of ultrasound applications for enhanced skin delivery and major outcomes.

Drug	Animal/Membrane Model Used	Outcomes	Reference
Salicylic acid and sucrose	Human cadaver skin (in vitro), ultrasound at 20 kHz	Up to ~1000-fold increase	[209]
Lidocaine (local anesthetic)	Clinical (human)	Reduced onset time, 60 min → 5 min (~12× faster functional delivery), as well as reduced pain	[210]
Mannitol (hydrophilic probe)	Pig skin (in vitro) + SLS (20 kHz, 10 W/cm^2^, 10% pulsed mode over 90 min)	~200-fold increase (vs. native skin)	[211]
Glycerol and dextran (4–150 kDa)	Pig skin (in vitro) with microneedle + US	~3-fold increase for glycerol and ~2-fold for dextran vs. single method	[212,213]
Calcein and bovine serum albumin (BSA) (≈66 kDa protein)	Pig skin (in vitro)	~3-fold increase	[208]
Insulin (~6 kDa)	Human skin (in vitro), animals (in vivo), 20 kHz, 62.6 to 225 mW/cm^2^, 100 ms pulses applied every second for 1 h, 100 U/mL insulin	Therapeutically relevant delivery; glucose reduction equivalent to subcutaneous injection (100 mU–1 U)	[214,215]
Interferon-γ (17 kDa)	Human cadaver skin, 20 kHz, 100 ms pulses applied every second for 4 h, 225 mW/cm^2^	Achieved therapeutic flux despite its higher molecular weight	[216]
Erythropoietin (48 kDa)	Human cadaver skin, 20 kHz, 100 ms pulses applied every second for 4 h, 225 mW/cm^2^	Achieved therapeutically relevant delivery across human cadaver skin	[216]

**Table 9:** Summary of applications of jet injectors for skin delivery and major outcomes.

Drug/Model	Experimental Condition	Outcomes	Reference
Minoxidil (hydrophilic)	Nebulized jet stream (20 shots, 30 s) vs. passive (0.5–3 h) in porcine skin	Jet stream application led to ~2-fold increase vs. passive (3 h), ~11-fold increase vs. passive (2 h), and ~2.6-fold increase (24 h cumulative release), with an indication of deeper penetration of the SC partition limitation of hydrophilic drugs.	[220]
FITC–dextran (macromolecule)	Nebulized jet stream vs. passive (0.5–2 h) in porcine skin	Nebulized jet stream enhanced macromolecule delivery by ~2-fold (0.5 h), 3-fold (1 h), and 4-fold (2 h).	[220]
Flavanones	Nebulized jet stream (10 shots) and passive (2 h) treatments in porcine skin	Flavanone permeation was 1.14-fold higher at 2 h.	[220]
Midazolam	Nebulized jet stream in humans	Intramuscular delivery of midazolam demonstrated similar bioequivalence but a significantly faster onset when the medication was delivered through the needle-free jet injector.	[221]
EMLA 5% cream (lidocaine 25 mg/g, prilocaine 25 mg/g)	Jet pressure, emanating from the nozzle, was scaled with voltage and varied between 2.21 MPa and 6.57 MPa when applied to patients	The findings revealed that Global Aesthetic Improvement Scale scores and patient satisfaction were significantly higher with the CureJet compared to the needle injection method.	[222]
Uridine-unmodified luciferase messenger ribonucleic acid (mRNA) and luciferase-encoding plasmid DNA	Jet injection in mouse and rat skin	Jet injection increased naked mRNA-mediated luciferase expression by approximately 100–200-fold in mice and 2000–3000-fold in rats compared with needle injection. mRNA expression was detectable within 0.5 h, peaked at 5–8 h.	[223]

**Table 13:** Clinical and commercial translation of representative topical and transdermal delivery systems.

Delivery Category/Product	Regulatory or Clinical Status	Indication/Target	Key Physicochemical, Dosing, or Clinical Evidence	Delivery-Specific Translational Significance and Remaining Limitation	Reference
A. Established passive cutaneous and transdermal products					
Nicotine transdermal patch	Established marketed product (E6)	Smoking cessation	MW 162.23 Da; LogP ~1.2. Step-down patches commonly deliver 21, 14, or 7 mg over 24 h.	Low molecular weight and modest lipophilicity permit sustained systemic input. Success nevertheless depends on controlled patch release and a moderate daily replacement dose rather than unrestricted skin permeability.	[265]
Fentanyl transdermal patch	Established marketed product (E6)	Severe persistent pain in opioid-tolerant patients	MW 336.5 Da; LogP ~4.0. A 72 h patch provides systemic input at a microgram-per-hour rate.	High potency and a low required dose make passive systemic delivery feasible. Lipophilicity promotes SC partitioning and depot formation, but heat exposure, dose dumping, accidental transfer, and interindividual variability present important safety risks.	[266]
Estradiol transdermal system	Established marketed product (E6)	Menopausal symptoms and selected osteoporosis-prevention use	MW 272.4 Da; LogP ~4.0. Once- or twice-weekly systems deliver approximately 0.025–0.1 mg/day.	Favorable molecular size, lipophilicity, and potency enable therapeutic systemic exposure at a low daily input while avoiding hepatic first-pass metabolism.	[267]
Clonidine transdermal patch	Established marketed product (E6)	Hypertension	MW 230.09 Da; LogP ~1.6. Weekly transdermal administration.	Small size, suitable amphiphilicity, and low-dose requirements support sustained systemic delivery. Limitations include delayed onset, rebound hypertension after abrupt withdrawal, adhesion problems, and local application site reactions.	[268]
Rivastigmine transdermal patch	Established marketed product (E6)	Dementia of Alzheimer’s type and Parkinson’s disease dementia	MW 250.34 Da; LogP ~2.3. Once-daily patch administration.	Sustained transdermal input can reduce peak-related gastrointestinal intolerance relative to oral capsules. Clinical performance remains dose-limited and depends on adhesion, skin tolerability, caregiver adherence, and correct patch replacement.	[269]
Testosterone transdermal gel	Established marketed product (E6)	Testosterone replacement therapy	MW 288.4 Da; LogP ~3.3. Hydroalcoholic gel applied over a relatively large skin area.	Steroidal lipophilicity facilitates skin partitioning, but the milligram-scale replacement dose requires high topical loading and large-area application. Secondary transfer to women or children remains a major safety concern.	[270]
Nitroglycerin transdermal patch	Established marketed product (E6)	Prevention of angina pectoris	MW 227.09 Da; LogP ~1.6. Sustained patch delivery; not intended for terminating acute attacks.	Small molecular size and high potency enable systemic delivery. Nitrate tolerance limits continuous use, requiring an appropriate nitrate-free interval.	[271]
Lidocaine 5% medicated patch	Established marketed product (E6)	Post-herpetic neuralgia	MW 234.34 Da; LogP ~2.3. Prolonged application of a high-dose adhesive patch to intact skin.	This is primarily a local dermal or superficial neural product rather than a systemic transdermal system. Its success reflects high local loading and prolonged contact and should not be interpreted as evidence of passive systemic delivery feasibility.	[272]
B. Approved formulation-enabled topical products					
Ruxolitinib cream 1.5% (Opzelura)	FDA approved for AD (2021) and nonsegmental vitiligo (2022); AD indication expanded to patients ≥2 years in 2025 (E6)	AD; nonsegmental vitiligo	TRuE-AD1/2: week-8 IGA success was 53.8% and 51.3% versus 15.1% and 7.6% with the vehicle. TRuE-V1/2: week-24 F-VASI75 response was 29.9% in both active groups versus 7.5% and 12.9% with the vehicle.	A solubilized oil-in-water cream enables localized JAK1/2 inhibition within defined dose and body surface area limits. The trials establish product-level efficacy but do not isolate a specific permeability-enhancing effect of the vehicle.	[247]
Tapinarof cream 1% (Vtama)	FDA approved for plaque psoriasis (2022) and AD in patients ≥2 years (2024) (E6)	Plaque psoriasis; AD	PSOARING 1/2: week-12 PGA response was 35.4% and 40.2% versus 6.0% and 6.3% with vehicle. ADORING 1/2: week-8 vIGA-AD success was 45.4% and 46.4% versus 13.9% and 18.0%.	The cream enables localized delivery of an AhR agonist across two inflammatory diseases. Vehicle-controlled efficacy establishes performance of the complete product, but the independent contribution of the vehicle to skin permeability was not quantified.	[248]
Roflumilast creams and foam (Zoryve)	FDA approved: cream 0.3% for plaque psoriasis (2022); foam 0.3% for seborrheic dermatitis (2023); cream 0.15% for AD (2024); foam 0.3% for scalp and body psoriasis (2025); cream 0.05% for AD in children aged 2–5 years (2025) (E6)	Plaque psoriasis, including intertriginous and scalp disease; AD; seborrheic dermatitis	DERMIS-1/2: week-8 IGA success was 42.4% and 37.5% versus 6.1% and 6.9%. INTEGUMENT-1/2: week-4 vIGA-AD success was 32.0% and 28.9% versus 15.2% and 12.0%. ARRECTOR: week-8 S-IGA success was 66.4% versus 27.8%.	The product family demonstrates dosage-form adaptation of a PDE-4 inhibitor to different diseases, ages, and anatomical sites. Foam facilitates application to hair-bearing areas, but clinical superiority over cream or ointment has not been established by direct comparison.	[249,250,251,252]
Delgocitinib cream 20 mg/g (Anzupgo)	EU marketing authorization in 2024; U.S. FDA approval in 2025 for adults only (E6)	Moderate-to-severe chronic hand eczema	IGA-CHE success was 19.7% versus 9.9% in DELTA 1 and 29.1% versus 6.9% in DELTA 2 for delgocitinib versus vehicle.	The cream delivers a pan-JAK inhibitor to fissured and hyperkeratotic hand lesions when topical corticosteroids are inadequate or inappropriate. Cutaneous concentrations and direct permeability enhancement were not measured.	[253,254,273]
Clascoterone cream 1% (Winlevi)	FDA approved in 2020 in patient ≥12 years (E6)	Acne vulgaris	In two phase III trials, week-12 IGA success was 18.4% and 20.3% versus 9.0% and 6.5% with vehicle; inflammatory and non-inflammatory lesion counts also decreased significantly.	Local androgen receptor antagonism provides an alternative to systemic antiandrogen treatment for a pilosebaceous disease. However, follicular deposition and pathway-specific delivery were not directly quantified.	[256]
Sofpironium topical gel 12.45% (Sofdra)	FDA approved in 2024 for ≥9 years old (E6)	Primary axillary hyperhidrosis	CARDIGAN 1/2: a ≥2-point HDSM-Ax-7 improvement at day 43 occurred in 49% and 64% versus 29% and 48% with vehicle. Median sweat-production changes were −128 versus −100 mg and −143 versus −134 mg per 5 min.	A metered gel provides the anatomically restricted anticholinergic treatment of axillary eccrine sweating. This represents site-specific topical pharmacology rather than the direct enhancement of intrinsic SC permeability; systemic exposure is reduced but not eliminated.	[257]
Berdazimer topical gel 10.3% (Zelsuvmi)	FDA approved in 2024 for all greater than 1 year (E6)	Molluscum contagiosum	Complete lesion clearance at week 12 was 32.4% versus 19.7% in one phase III trial and 30.0% versus 20.3% in another trial.	The two-component gel generates and releases nitric oxide locally at individual lesions. Its translational innovation is controlled active agent generation at the application site rather than enhancement of passive skin permeability.	[258]
Difamilast ointment 1% (Adquey)	FDA approved in February 2026 (E6)	Mild-to-moderate AD in patients ≥2 years	Week-4 IGA success in three vehicle-controlled trials was 21% versus 3%, 38% versus 13%, and 47% versus 18% for difamilast versus vehicle.	This approval demonstrates successful translation of another topical PDE-4 inhibitor in an ointment suitable for pediatric and adult AD. The trials establish product efficacy but do not distinguish the contribution of the ointment to permeability.	[255]
C. Device-assisted human delivery studies					
Hyaluronic acid-based dissolving MN patch applied after betamethasone/calcipotriol ointment	Randomized split-body clinical study published in 2025 (E5)	Hyperkeratotic psoriatic plaques	mPASI improvement was 80.4% with the MN patch, 64.6% with a no-needle patch, and 55.5% with ointment alone. In vitro delivery increased approximately 2.1-fold versus ointment alone.	MN-generated microchannels bypass the thickened SC. The no-needle control distinguishes microchannel-mediated delivery from occlusion, although delivery was not measured directly in human tissue and the study was small and short-term.	[259]
Microneedling or fractional MN radiofrequency combined with topical vitamin C/E/ferulic acid serum	Two randomized controlled split-face and split-neck clinical trials published in 2026 and 2025, respectively (E5)	Facial and neck photoaging	Conventional microneedling: marked or near-total GAIS improvement was 89.3% with antioxidant serum versus 7.1% with placebo. Fractional MN radiofrequency: wrinkle severity decreased by 29.9% versus 18.0%, and elasticity increased by 12.9% versus 2.3%.	Contralateral controls support an additional contribution from the topical formulation. However, intradermal antioxidant concentrations were not measured, and microneedling or radiofrequency can independently induce tissue remodeling.	[260,261]
Wearable NIR-responsive hyaluronic acid–gold nanorod MN patch	Randomized investigator-blinded pilot clinical study published in 2026 (E5)	Periorbital wrinkles	Under-eye roughness improved by 16.3% at week 4. Crow’s-feet improvement was approximately 14.1% with NIR actuation versus 1.9% with the non-actuated patch; no device-related adverse events were reported.	The system combines SC bypass with externally activated dissolution and local heating. The findings provide preliminary human evidence but require confirmation in larger, longer, and independently replicated studies.	[262]
Fractional CO_2_ laser-assisted topical triamcinolone acetonide, 5-fluorouracil, or vitamin C delivery	Randomized comparative and prospective clinical studies published in 2025 (E5)	Hypertrophic and atrophic scars	Laser-only and drug-combination groups improved scar severity, with treatment-specific changes in pliability, height, pigmentation, and vascularity. In a 97-patient study, modified Manchester scores decreased from 2.46 to 1.43 for atrophic scars and from 2.80 to 1.58 for hypertrophic scars.	Laser-generated microchannels permit immediate topical application to dense scar tissue. However, laser-induced remodeling independently improves scars, and incomplete control-group designs prevent definitive attribution of the clinical benefit to enhanced drug transport.	[263,264]

Note: Evidence levels follow the translational hierarchy defined in Table 12: E5, controlled human clinical evidence; E6, regulatory approval or established commercial use. Marketed product information in Part A was obtained from DailyMed (U.S. National Library of Medicine). Regulatory approval and clinical efficacy establish the successful translation of the complete drug product but do not necessarily demonstrate enhanced intrinsic SC permeability or distinguish the independent contribution of the vehicle. AD, atopic dermatitis; AhR, aryl hydrocarbon receptor; F-VASI75, ≥75% improvement in the Facial Vitiligo Area Scoring Index; GAIS, Global Aesthetic Improvement Scale; HDSM-Ax-7, 7-item Hyperhidrosis Disease Severity Measure–Axillary; IGA, Investigator Global Assessment; IGA-CHE, Investigator Global Assessment for Chronic Hand Eczema; JAK, Janus kinase; MN, microneedle; mPASI, modified Psoriasis Area and Severity Index; NIR, near-infrared; PDE-4, phosphodiesterase-4; PGA, Physician Global Assessment; SC, stratum corneum; S-IGA, Scalp Investigator Global Assessment; vIGA-AD, validated Investigator Global Assessment for Atopic Dermatitis.

## Data Availability

No new data were created or analyzed in this study. Data sharing is not applicable to this article.

## Supplementary Materials

The following supporting information can be downloaded at: https://www.mdpi.com/article/10.3390/pharmaceutics18091069/s1, Figure S1: Representative chemical structures of alcohol-, amide-, ester-, ether alcohol-, pyrrolidone-, and sulfoxide-based chemical permeation enhancers; Figure S2: Representative chemical structures of fatty acid-, glycol-, surfactant-, and terpene-based chemical permeation enhancers; Figure S3: Formulation platforms incorporating chemical permeation enhancers for topical and transdermal drug delivery; Figure S4: Representative vesicular carrier systems and their structural characteristics relevant to skin delivery.

## Abbreviations

The following abbreviations are used in this manuscript:

AD	Atopic dermatitis
AhR	Aryl hydrocarbon receptor
AKBA	3-Acetyl-11-keto-β-boswellic acid
AUC	Area under the concentration–time curve
AUC_0–24 h_	Area under the concentration–time curve from 0 to 24 h
BSA	Bovine serum albumin
CD/CDs	Cyclodextrin(s)
C_max_	Maximum plasma concentration
CMCs	Critical micelle concentrations
CPE/CPEs	Chemical permeation enhancer(s)
DBMNP/DBMNPs	Dissolved bubble microneedle patch(es)
DEGEE	Diethylene glycol monoethyl ether
DMN/DMNs	Dissolving microneedle(s)
DMSO	Dimethyl sulfoxide
DNA	Deoxyribonucleic acid
DPG	Dipotassium glycyrrhizinate
E1	In vitro release, permeation, reconstructed-skin, or synthetic-membrane evidence
E2	Ex vivo animal- or human-skin evidence
E3	In vivo animal pharmacokinetic, tissue-distribution, efficacy, or safety evidence
E4	Human pharmacokinetic, dermatokinetic, tissue-deposition, or mechanistic evidence
E5	Controlled clinical evidence in humans
E6	Regulatory approval or established commercial use
EMLA	Eutectic mixture of local anesthetics
ER_flux_	Flux enhancement ratio
Er:YAG	Erbium-doped yttrium aluminum garnet
EU	European Union
EV/EVs	Extracellular vesicle(s)
FDA	U.S. Food and Drug Administration
FITC	Fluorescein isothiocyanate
FOXM1	Forkhead box M1
GAIS	Global Aesthetic Improvement Scale
G-CSF	Granulocyte colony-stimulating factor
GelMA	Gelatin methacryloyl
GFP	Green fluorescent protein
HAMA	Hyaluronic acid methacrylate
HbA1c	Glycated hemoglobin A1c
HPC	Hydroxypropyl cellulose
HPMC	Hydroxypropyl methylcellulose
IFN-γ	Interferon gamma
IGA	Investigator Global Assessment
IGA-CHE	Investigator Global Assessment for chronic hand eczema
IPM	Isopropyl myristate
JAK	Janus kinase
LHRH	Luteinizing hormone-releasing hormone
logD	Logarithm of distribution coefficient
LogP	Logarithm of partition coefficient
L-Pro2	N-acetyl-L-proline dodecyl ester
MC	Methylcellulose
MCP-1	Monocyte chemoattractant protein-1
MN/MNs	Microneedle(s)
MP	Methyl paraben
mPASI	Modified Psoriasis Area and Severity Index
mRNA	Messenger RNA
MRT	Mean residence time
MSC	Mesenchymal stem cell(s)
MW	Molecular weight
NaCMC	Sodium carboxymethyl cellulose
NIR	Near-infrared
NMP	N-methyl-2-pyrrolidone
NSAID	Nonsteroidal anti-inflammatory drug
PBS	Phosphate-buffered saline
PD-L1	Programmed death-ligand 1
PEG/PEGs	Polyethylene glycol(s)
PG	Propylene glycol
PRISMA	Preferred Reporting Items for Systematic Reviews and Meta-Analyses
PVP	Polyvinylpyrrolidone
Q24	Cumulative amount permeated at 24 h
ROS	Reactive oxygen species
SC	Stratum corneum
SEMA	Semaglutide
sEV/sEVs	Small extracellular vesicle(s)
siRNA	Small interfering RNA
SLN/SLNs	Solid lipid nanoparticle(s)
SLS	Sodium lauryl sulfate
t_1/2_	Elimination half-life
TEWL	Transepidermal water loss
TGF-β1	Transforming growth factor beta 1
TPGS	D-α-tocopheryl polyethylene glycol 1000 succinate
TRV/TRVs	Tiered-release vesicle(s)
