# Supplementary material for: Skin Barrier-Informed Topical and Transdermal Drug Delivery: Excipient-Driven Strategies, Vehicle Transformation, and Translational Challenges
Source: Pharmaceutics. 2026 Aug 26;18(9):1069. doi: 10.3390/pharmaceutics18091069 (PMC13610917; doi:10.3390/pharmaceutics18091069)
Supplement: Supplementary file 1 [file pharmaceutics-18-01069-s001.zip › pharmaceutics-4473120-supplementary.pdf]

Supplemental Materials

Skin Barrier-Informed Topical and Transdermal Drug Delivery: Excipient-Driven Strategies, Vehicle Transformation, and Translational Challenges

Binaya Sapkota [1,†], Susmita Phuyal [1,†], Arjun Dhwoj Bamjan [1], Seung-Sik Cho [1,2], Jung-Hyun Shim [1,2], Jin Woo Park [1,2,*] and Laxman Subedi [1,3,*]

[1] Department of Biomedicine, Health & Life Convergence Sciences, BK21 Four, Biomedical and Healthcare Research Institute, Mokpo National University, Muan-gun, Jeonnam 58554, Republic of Korea; phr.binaya@gmail.com (B.S.); sushmitaphuyal54@gmail.com (S.P.); arjun.bamjan@gmail.com (A.D.B.); sjason1@naver.com (S.-S.C.); s1004jh@gmail.com (J.-H.S.); jwpark@mokpo.ac.kr (J.W.P.); laxmansubedi789@gmail.com (L.S.)

[2] College of Pharmacy and Natural Medicine Research Institute, Mokpo National University, Muan-gun, Jeonnam 58554, Republic of Korea

[3] Biomedicine Cutting Edge Formulation Technology Center, Mokpo National University, Muan-gun, Jeonnam 58554, Republic of Korea

* Correspondence: jwpark@mokpo.ac.kr (J.W.P.); laxmansubedi789@gmail.com (L.S.)

† These authors contributed equally to this work.

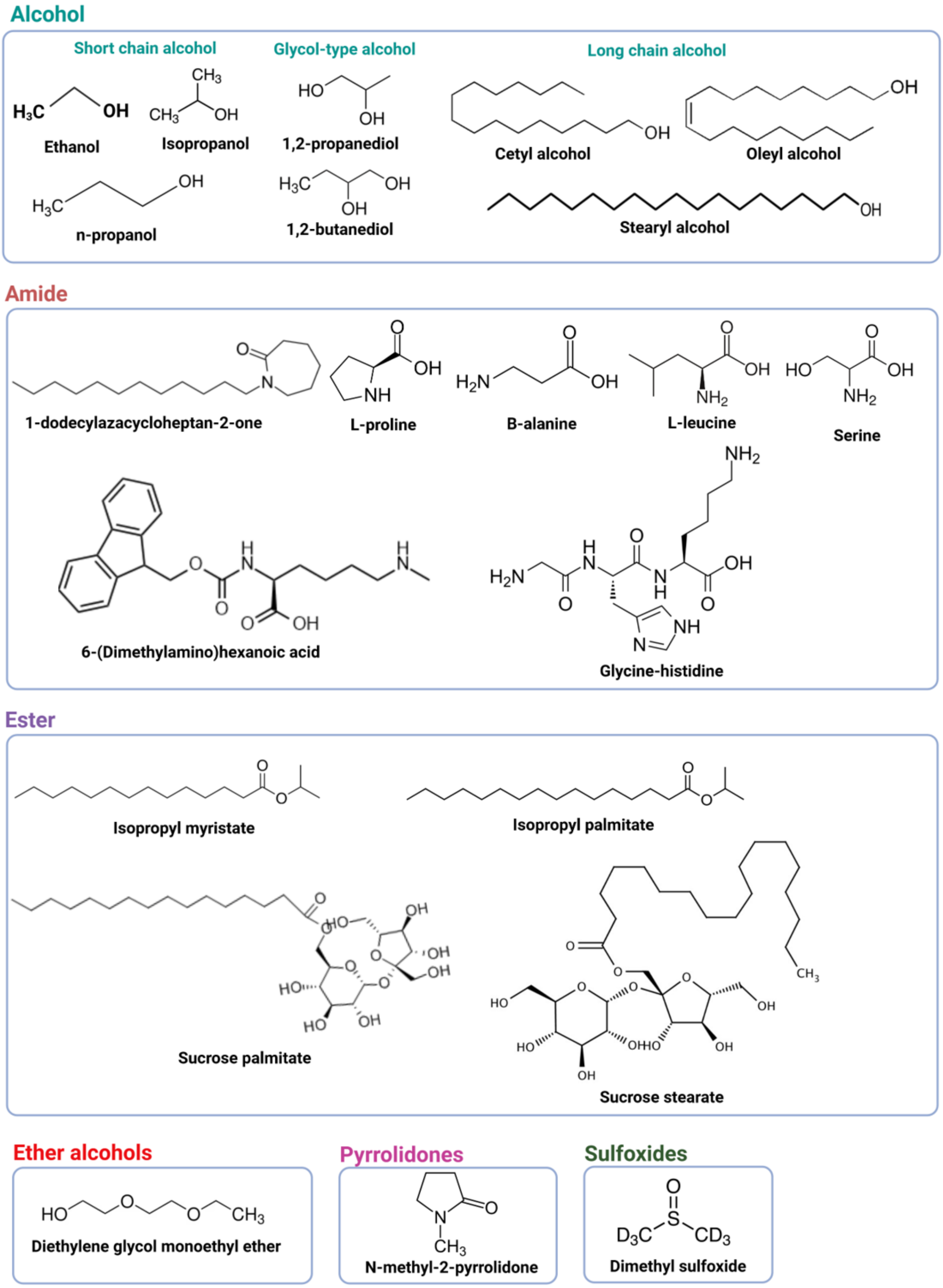

Figure S1. Chemical structures of representative chemical permeation enhancers (CPEs) from major classes alcohol, amide, esters, ether alcohols, pyrolidones, and sulfoxides. The structures illustrate key determinants of CPE activity based on alkyl-chain length, branching, saturation/unsaturation, and functional groups.

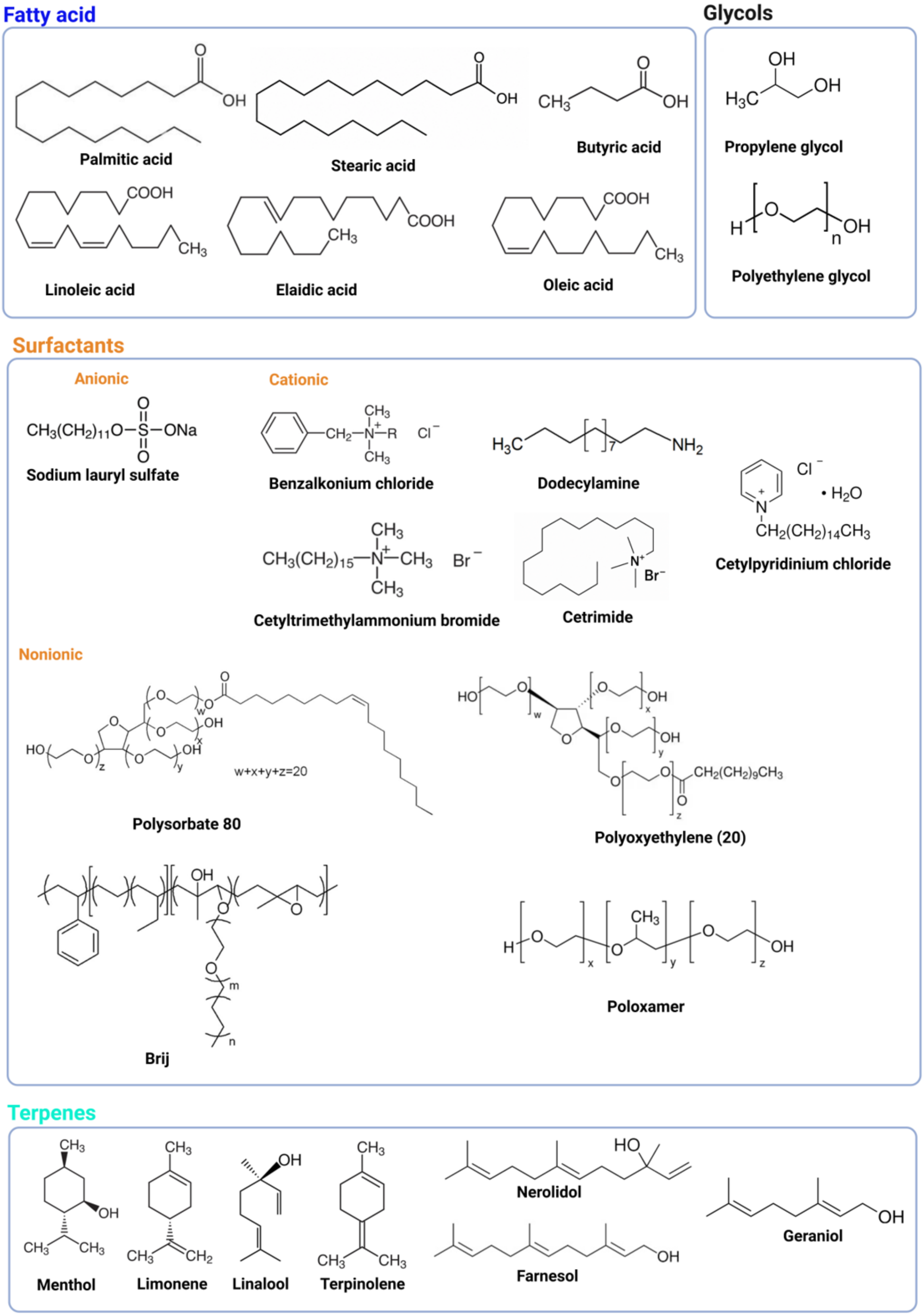

Figure S2. Chemical structures of representative chemical permeation enhancers (CPEs) from major classes fatty acids, glycols, surfactants, and terpenes. The structures illustrate key determinants of CPE activity based on alkyl-chain length, branching, saturation/unsaturation, and functional groups.

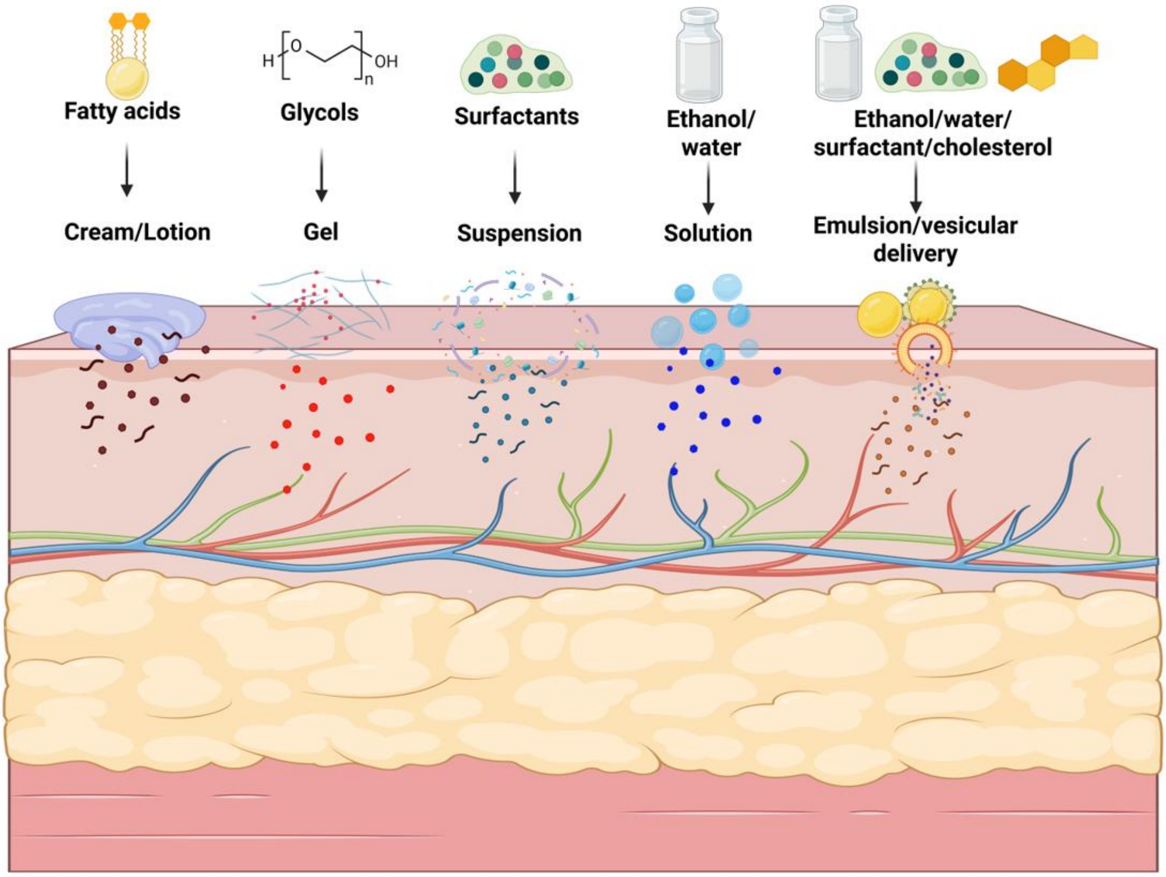

Figure S3. Formulation platforms for chemical permeation enhancers (CPE)-assisted topical and transdermal drug delivery. CPEs are incorporated into conventional vehicles, including creams/lotions, gels, suspensions, and solutions, as well as advanced systems such as emulsions, liposomes, ethosomes, transfersomes, and niosomes. Their permeation-enhancing effect depends on the overall formulation, where they improve drug solubilization, thermodynamic activity, stratum corneum partitioning, lipid modulation, and cutaneous or transdermal drug transport.

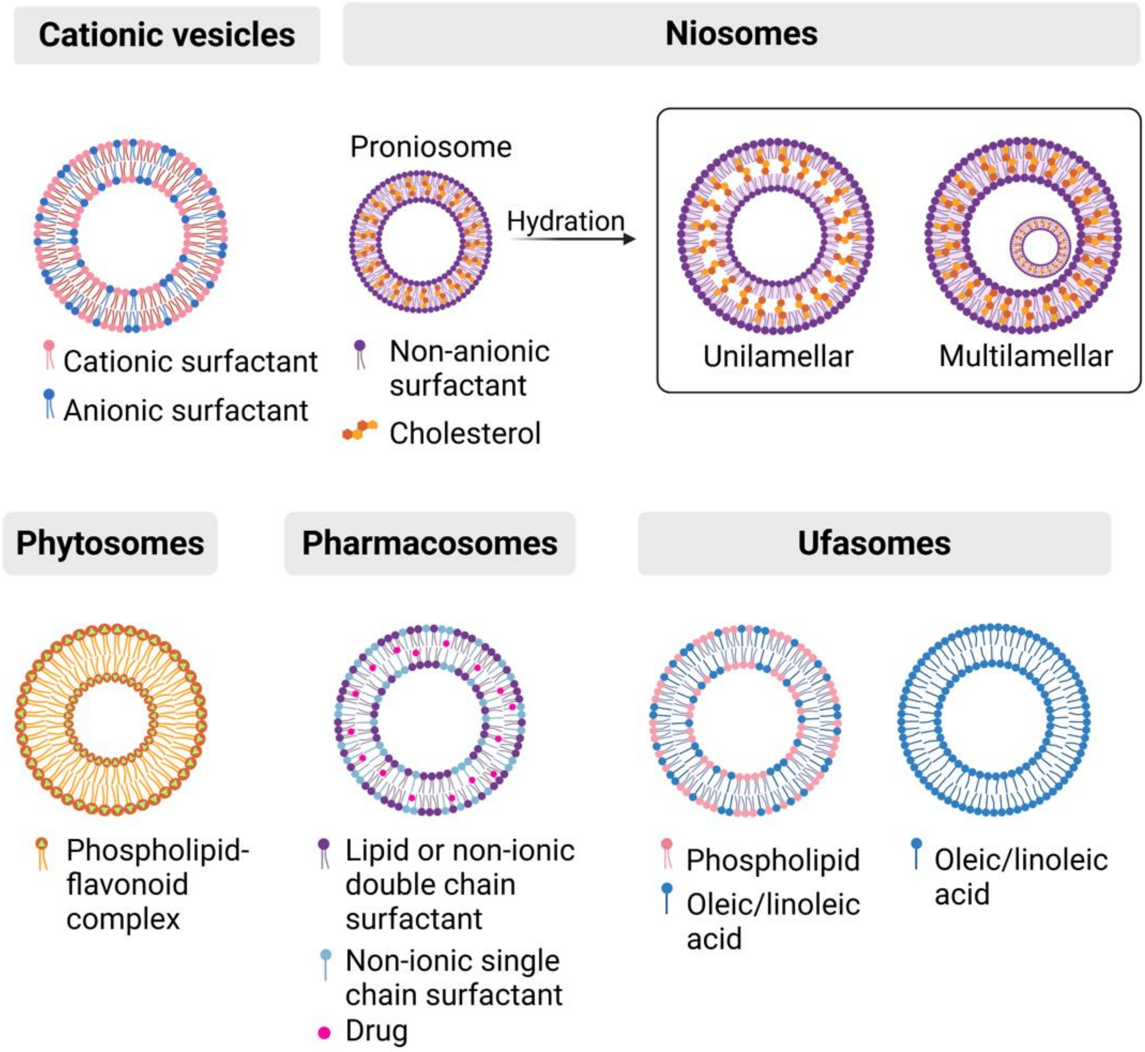

Figure S4. Representative vesicular systems for topical/transdermal delivery. The schematic shows cationic vesicles, niosomes, phytosomes, pharmacosomes, and ufasomes, highlighting their key structural components, charge characteristics, lamellarity, and lipid/surfactant organization that influence drug loading, skin interaction, and permeation performance.
